# SIRT6 promotes metastasis and relapse in HER2-positive breast cancer

**DOI:** 10.1038/s41598-023-49199-7

**Published:** 2023-12-12

**Authors:** Cristina Andreani, Caterina Bartolacci, Giuseppe Persico, Francesca Casciaro, Stefano Amatori, Mirco Fanelli, Marco Giorgio, Mirco Galié, Daniele Tomassoni, Junbiao Wang, Xiaoting Zhang, Gregory Bick, Roberto Coppari, Cristina Marchini, Augusto Amici

**Affiliations:** 1 Department of Biosciences and Veterinary Medicine, University of Camerino, 62032 Camerino, Italy; 2 Present Address: Department of Internal Medicine, University of Cincinnati, 45219 Cincinnati, OH USA; 3 Department of Experimental Oncology, IRCCS—European Institute of Oncology, Via Adamello 16, 20139 Milano, Italy; 4 Department of Biomedical Sciences, University of Padua, Via Ugo Bassi 58/B, 35131 Padua, Italy; 5 Molecular Pathology Laboratory “PaoLa”, Department of Biomolecular Sciences, University of Urbino Carlo Bo, 61032 Fano, Italy; 6 Department of Neuroscience, Biomedicine and Movement, Section of Anatomy and Histology, University of Verona, 37134 Verona, Italy; 7 Department of Cancer Biology, University of Cincinnati, 45219 Cincinnati, OH USA; 8 Department of Cell Physiology and Metabolism, University of Geneva, 1211 Geneva, Switzerland; 9 Diabetes Center of the Faculty of Medicine, University of Geneva, 1211 Geneva, Switzerland

**Keywords:** Breast cancer, Cancer models, Epigenetics, Gene regulation, Tumour biomarkers, Metastasis

## Abstract

The histone deacetylase sirtuin 6 (SIRT6) has been endowed with anti-cancer capabilities in many tumor types. Here, we investigate the impact of SIRT6-overexpression (SIRT6-OE) in Delta16HER2 mice, which are a *bona fide* model of HER2-positive breast cancer. After an initial delay in the tumor onset, SIRT6-OE induces a more aggressive phenotype of Delta16HER2 tumors promoting the formation of higher number of tumor foci and metastases than controls. This phenotype of SIRT6-OE tumors is associated with cancer stem cell (CSC)-like features and tumor dormancy, and low senescence and oxidative DNA damage. Accordingly, a sub-set of HER2-positive breast cancer patients with concurrent SIRT6-OE has a significant poorer relapse-free survival (RFS) probability than patients with low expression of SIRT6. ChIP-seq, RNA-seq and RT-PCR experiments indicate that SIRT6-OE represses the expression of the T-box transcription factor 3 (*Tbx3*) by deacetylation of H3K9ac. Accordingly, loss-of-function mutations of *TBX3* or low *TBX3* expression levels are predictive of poor prognosis in HER2-positive breast cancer patients. Our work indicates that high levels of SIRT6 are indicative of poor prognosis and high risk of metastasis in HER2-positive breast cancer and suggests further investigation of TBX3 as a downstream target of SIRT6 and co-marker of poor-prognosis. Our results point to a breast cancer subtype-specific effect of SIRT6 and warrant future studies dissecting the mechanisms of SIRT6 regulation in different breast cancer subtypes.

## Introduction

Among the mammalian Sir2 homologues, SIRT6 has drawn interest over the last decades, given its broad-spectrum contribution in multiple biological processes. In this scenario numerous studies on *Sirt6* null and transgenic models have endowed this sirtuin with both anti-aging^1–5^ and anti-cancer properties^6–13^. SIRT6 can act as a tumor suppressor intervening in DNA repair, genomic maintenance, cell metabolism and directly co-repressing several tumor-promoting genes. However, although SIRT6 is significantly down-regulated in several human cancers^6,14–17^, a more detailed analysis in different tumor types has rendered its role quite puzzling, suggesting that SIRT6 can exert also pro-tumor effects depending on the cell/tissue of origin or disease stage^18–21^.

Such double-faced behavior of SIRT6 is particularly evident in heterogeneous diseases like breast cancer, which is characterized by distinct tumor subtypes. In this regard, while SIRT6 up-regulation has been linked to paclitaxel/epirubicin resistance and increased in vitro invasion^22,23^, *SIRT6* overexpression (SIRT6-OE) in basal-like breast tumor models was shown to both suppress the cancer stem cell (CSC) properties^24^ and to enhance tumorigenesis via oxidative phosphorylation^25^.

On the other hand, even if SIRT6 loss has been reported to induce Trastuzumab-resistance in HER2-overexpressing breast cancer^26^, very little is known about the real role of SIRT6 in this breast cancer subtype that represents about 30% of all mammary tumor cases.

In this study, we sought to determine whether SIRT6-OE impacts mammary tumorigenesis in Delta16HER2 transgenic mice, which develop spontaneous aggressive mammary carcinomas with an early onset^27^ and a detectable expression of estrogen receptor (ER)^28^. Thus, Delta16HER2 transgenic mice are suitable to test anti-HER2 therapies and exhibit immunologic tolerance to human HER2 antigen, faithfully recapitulating what is encountered clinically^27–31^.

Delta16HER2 is a naturally occurring splice variant of HER2 which is commonly co-expressed with the wild type protein in 52 to 90% of HER2-overexpressing breast cancers^32–35^. Delta16HER2 has emerged as the HER2 oncoprotein variant responsible for transformation, enhanced tumorigenic potential and resistance to HER2-targeted therapies, including monoclonal antibodies and tyrosine kinase inhibitors^29,31,33,34,36,37^. Controversially, some authors reported that Delta16HER2 promotes the sensitivity to Trastuzumab in breast cancer models^32,38^. These discrepancies might be due to the use of xenografts in immunocompromised mice, that bypass the complexity and the influence of the immune system^32^ and/or by the potential contribution of anti-drug antibodies elicited by the humanized Trastuzumab in non-humanized mouse models^38^. On the contrary, the role of Delta16HER2 in driving the resistance to HER2- and EGFR-targeted therapies is supported not only by work done by our group using physiologically-relevant breast cancer models^31,36^, but also in HER2-positive gastric cancer patients and EGFR L858R/T790M-Positive Non-Small Cell Lung Cancer^39,40^.

*TBX*3 (*Tbx3* in mouse) is a member of the T-box transcription factor family which is widely conserved across species. TBX3 can act as gene co-repressor/co-activator by partially binding the palindromic sequence (T(G/C)ACACCTAGGTGTGAAATT) known as the T-element, or half sites within this sequence^41^.

In mice, the homozygous knockout of *Tbx3* completely ablates the development of mammary glands^42,43^. In humans, loss of function mutations in *TBX3* result in the Ulnar-Mammary Syndrome (UMS), an autosomal dominant condition characterized by mammary gland hypoplasia and other congenital anomalies. As for SIRT6, altered TBX3 levels may play different and opposite roles in cancer. Indeed, while large evidence suggests oncogenic roles for TBX3^41,42,44,45^, a few studies have indicated that it may also have tumor suppressor functions^46,47^, suggesting that TBX3 function may depend on cellular context-specific factors and on its protein partners/regulators. Indeed, TBX3 is known to interact with and to be regulated by several epigenetic modifiers, including Sirtuin 1 (SIRT1)^48–51^.

Here, we identified *TBX3* as a target downregulated by SIRT6 in both Delta16HER2- and HER2-positive breast cancer models, and we found that concomitant high *SIRT6* and low *TBX3* expression predicts poor prognosis in HER2-positive breast cancer patients.

## Results

### SIRT6 initially delays, but later promotes Delta16HER2-driven tumorigenesis

**Figure 1:**
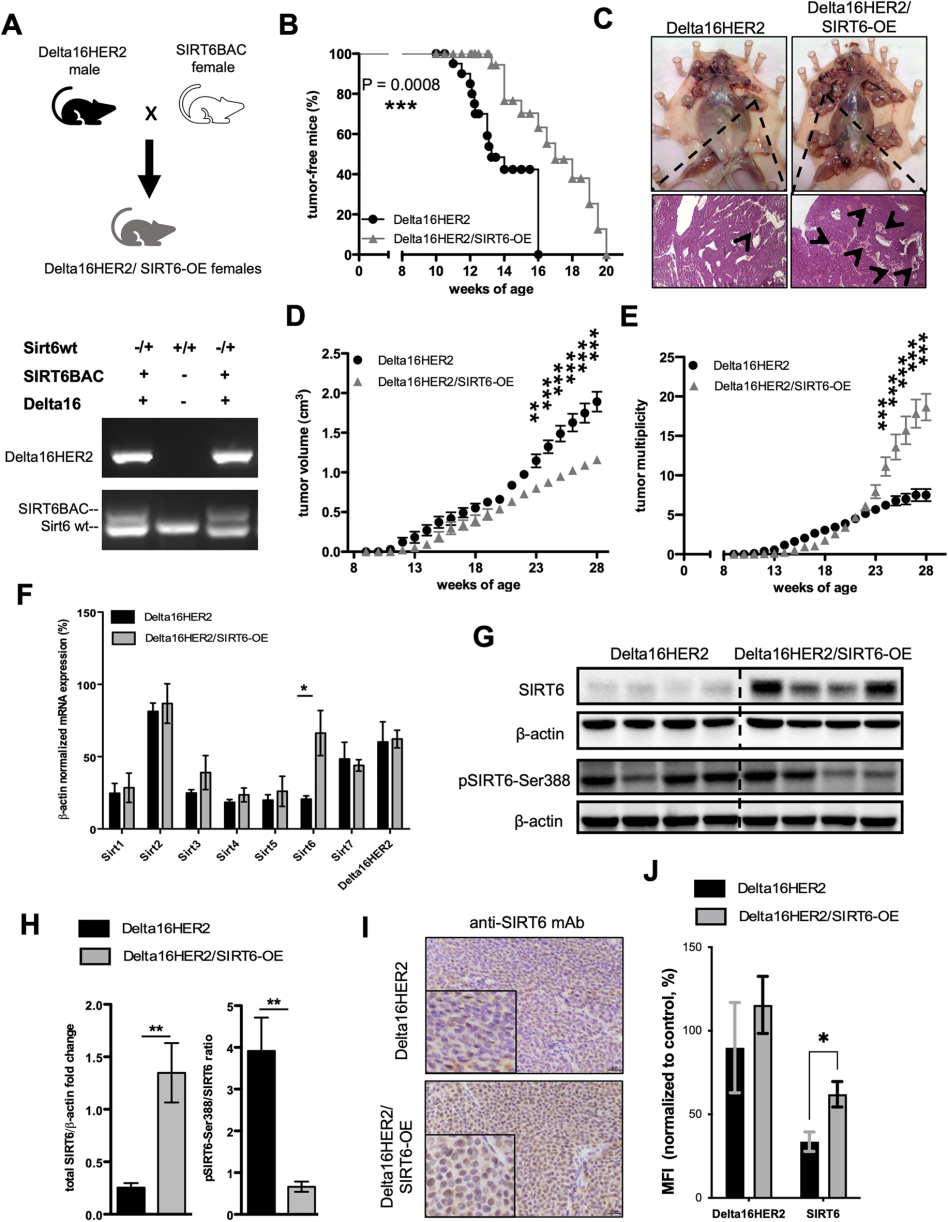
SIRT6 initially delays Delta16HER2-driven tumorigenesis, but then promotes a high tumor multiplicity. (**A**) Breeding scheme used to obtain Delta16HER2/SIRT6-OE female mice and a representative genotyping PCR. (**B**) Kaplan-Meir curves comparing the percentage of tumor-free mice between Delta16HER2 controls and Delta16HER2/SIRT6-OE mice (n = 17/group). (**C**) Representative *post mortem* pictures of Delta16HER2 and Delta16HER2/SIRT6-OE mice at 30 weeks of age (top panels), and relative H&E staining of the primary mammary tumor masses (bottom panels). Black arrows indicate tumor foci. (**D**,**E**) Tumor multiplicity and tumor growth curves of Delta16HER2 and Delta16HER2/SIRT6-OE mice (n = 17/group). (**F**) mRNA expression of *Sirtuin1-7(Sirt1-7)* and *Delta16HER2* normalized to *β-Actin* mRNA level in tumors of 30-week-old Delta16HER2 controls and Delta16HER2/SIRT6-OE mice (n = 4/group). (**G**) Representative images of IHC staining for SIRT6 (brown) in tumors of 30-week-old Delta16HER2 and Delta16HER2/SIRT6-OE mice, respectively. Delta16HER2/SIRT6-OE tumors show a strong SIRT6 level and mainly localized into the nucleus. Scale bar, 25 mm. (**H**,**I**) Western blot analysis and relative quantification of total SIRT6 and phosphorylated SIRT6 (pSIRT6-Ser388) normalized to β-Actin protein level. pSIRT6-Ser388/total SIRT6 represents the ratio of β-Actin-normalized phosphorylated protein over the total SIRT6 protein. (**J**) Flow cytometry staining for Delta16HER2 and SIRT6 of primary cells (passage 4 in vitro) derived from tumors of Delta16HER2 and Delta16HER2/SIRT6-OE mice at 30 weeks of age. MFI, Median Fluorescence Intensity. The experiment was carried out in triplicates. In (**B**) ***p = 0.0008 (Log-rank test); in (**D**,**E**) **p < 0.01, ***p < 0.001 (two-way ANOVA followed by Sidak’s multiple comparisons test); in (**F**,**H**,**J**) *p < 0.05, **p < 0.01 (two-tailed unpaired *t* test). Error bars represent SD. See also Fig. S1.

To determine whether SIRT6 affects Delta16HER2-dependent mammary carcinogenesis, we generated Delta16HER2/SIRT6-OE mice by breeding the Delta16HER2 breast cancer model with the functionally competent Sirt6BAC mice^52^ which have a two-fold SIRT6-OE (Fig. 1A). Interestingly, Delta16HER2/SIRT6-OE female mice exhibit a significantly delayed tumor onset when compared to Delta16HER2 littermates (Fig. 1B). However, starting at 20 weeks of age, Delta16HER2/SIRT6-OE group starts to suffer from an increasing number of tumor lesions, but smaller in size with respect to Delta16HER2 controls (Fig. 1C–E). Using quantitative real-time PCR (qRT-PCR) we demonstrated that SIRT6 is significantly over-expressed in Delta16HER2/SIRT6-OE tumors without perturbating the expression of other Sirtuins and Delta16HER2 (Fig. 1F). Accordingly, IHC and Western blot assays show that Delta16HER2/SIRT6-OE tumors have higher SIRT6 protein levels with respect to Delta16HER2 controls (Fig. 1G–I). Moreover, the low ratio of phosphorylated SIRT6 at Ser388 over total SIRT6 suggests that the over-expressed SIRT6 is mainly nuclear and active in Delta16HER2/SIRT6-OE tumors (Fig. 1H and I). Also, SIRT6 over-expression is maintained in Delta16HER2/SIRT6-OE primary tumor cell cultures (Fig. 1J).

To further confirm that the in vivo phenotype of Delta16HER2/SIRT6-OE mice was effectively due to SIRT6-OE, we generated mice possessing Sirt6BAC that are homozygous null for the endogenous *Sirt6* gene (Delta16HER2/SIRT6-OE/Sirt6^–/–^) (Fig. S1A). Notably, no significant differences were detected between Delta16HER2/SIRT6-OE/Sirt6^-/-^ and Delta16HER2 mice in terms of tumor-free survival (Fig. S1B), tumor multiplicity and volume (Fig. S1C–E), confirming that SIRT6-OE accounts for the phenotype of Delta16HER2/SIRT6-OE mice.

### SIRT6 promotes tumor cell migration, invasion and lung metastasis in Delta16HER2/SIRT6-OE mice

**Figure 2:**
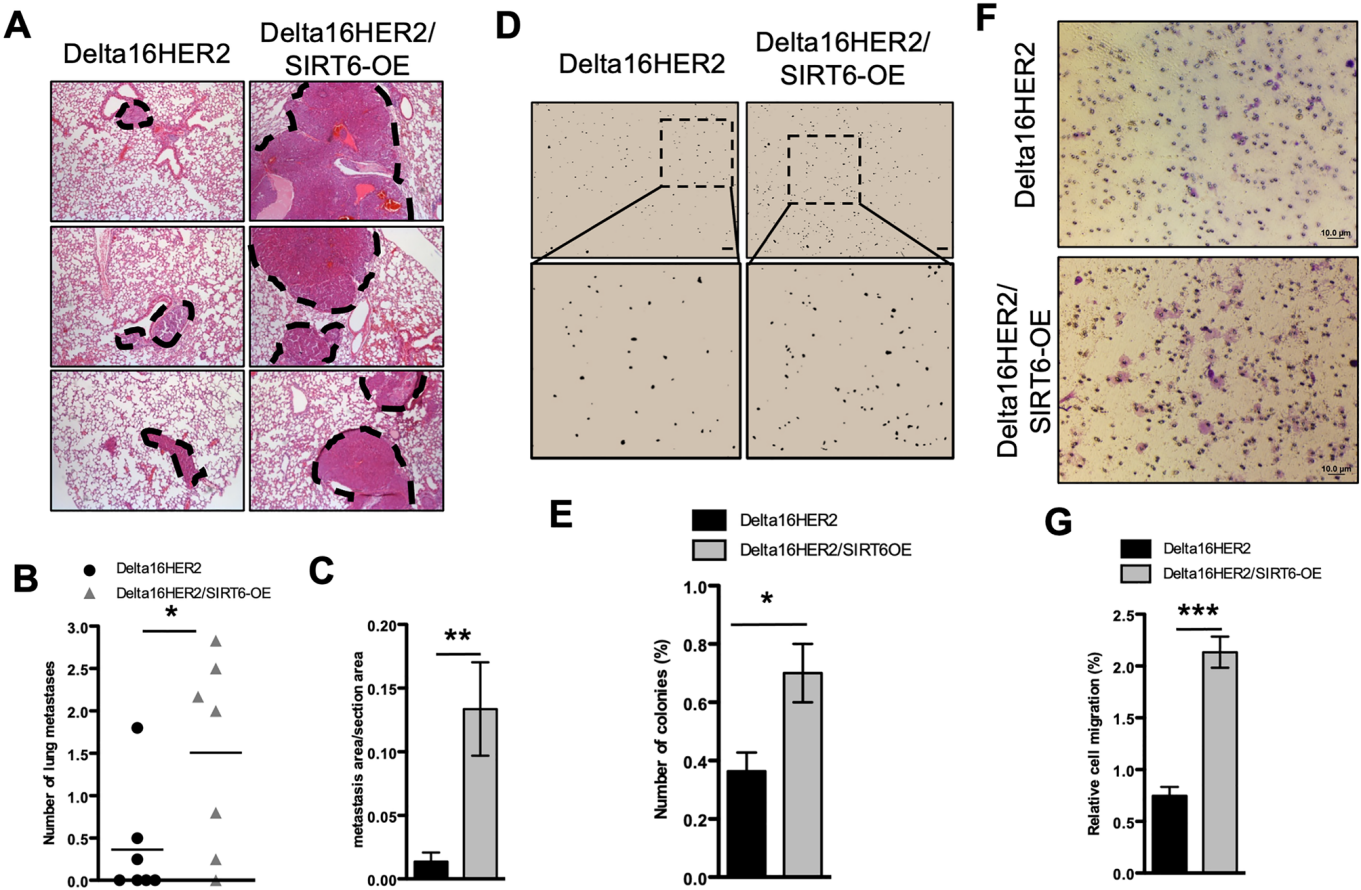
SIRT6 boosts tumor cell migration, invasion and lung metastasis in Delta16HER2/SIRT6-OE mice. (**A**) Representative pictures of H&E staining on lungs of Delta16HER2 and Delta16HER2/SIRT6-OE mice at 30 weeks of age (n = 7/group). Dashed black lines highlight the metastatic area in each picture. Quantification is represented as number of metastasis per mouse (**B**) and as metastasis area over total section area (**C**). Both number and area of metastasis were calculated as average over 2–6 consecutive tissue sections per mouse. (**D**) Soft agar assay performed on primary cells (passage 4 in vitro) derived from tumors of Delta16HER2 and Delta16HER2/SIRT6-OE mice at 30 weeks of age. Stereomicroscope images, 4X magnification (top) and 10X zoom (bottom). Number of colonies (**E**) were quantified using ImageJ software. Results are expressed as % of total number of seeded cells. The assay was performed in triplicates. (**F**,**G**) Transwell migration assay and quantification of Delta16HER2 and Delta16HER2/SIRT6-OE primary cells. Pore size 8.0 mm. Quantification is reported as percentage of migrating cells over the total number of seeded cells. In (**B**,**C**,**E**,**G**) *p < 0.05, **p < 0.01, ***p < 0.001 (two-tailed unpaired *t* test). Error bars represent SD.

The Delta16HER2 model is prone to develop lung metastases around 25 weeks of age^27^. To evaluate whether SIRT6-OE impacts metastasis formation, we analyzed lungs from 30-week-old mice. Strikingly, H&E staining of lungs shows that Delta16HER2/SIRT6-OE mice suffer from a higher number of metastases than Delta16HER2 counterparts (Fig. 2A and B). Moreover, a significant discrepancy in terms of metastatic area per section area has been found between the two groups, with Delta16HER2/SIRT6-OE mice having bigger lung metastatic lesions than controls (Fig. 2A and C). Such phenotype is recapitulated in vitro by primary cell cultures derived from both Delta16HER2/SIRT6-OE and Delta16HER2 tumors. Indeed, Delta16HER2/SIRT6-OE cells display an increased colony formation capability in soft agar (Fig. 2D–E) as well as an improved migratory capability through trans-well membranes (Fig. 2F and G).

### SIRT6 protects Delta16HER2 tumor cells from G2/M arrest, senescence and oxidative DNA damage

**Figure 3:**
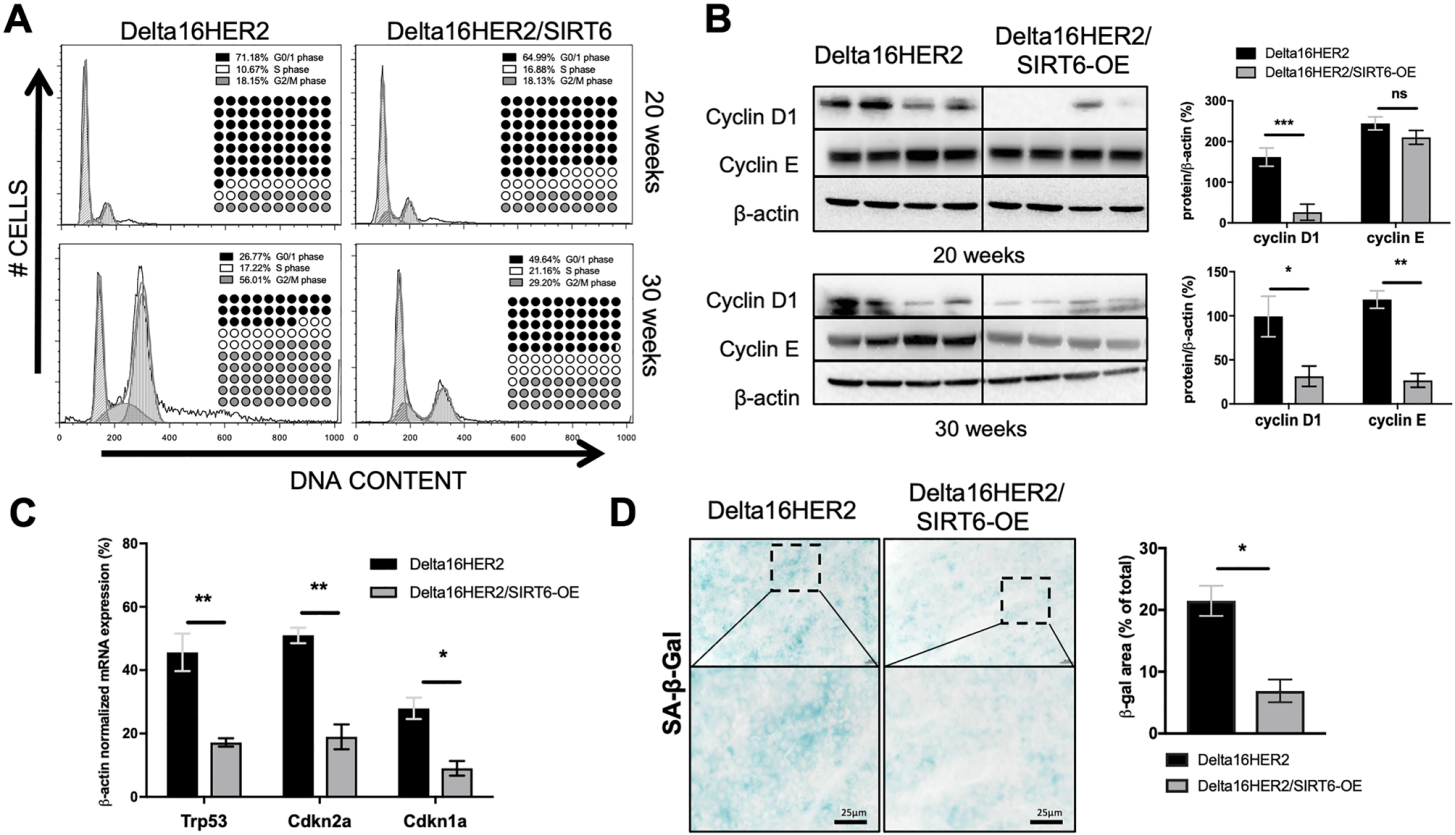
SIRT6 prevents tumor cells from Delta16HER2-induced G2/M arrest and senescence. (**A**) Ex vivo cell cycle analysis of cell suspensions derived from Delta16HER2 and Delta16HER2/SIRT6-OE tumors at 20 weeks of age (top panels) and at 30 weeks of age (bottom panels). DNA content was evaluated by flow cytometry using propidium iodide (PI) staining (n = 3) and then analyzed using Dean-Jett-Fox algorithm in FlowJo software. Percentages of cells in each cell cycle phase are summarized in each panel. (**B**) Immunoblot image and quantification of Cyclin D1 and Cyclin E levels normalized to β-Actin in tumors of either 20- (top) or 30-week-old (bottom) Delta16HER2 and Delta16HER2/SIRT6-OE mice (n = 4). (**C**) mRNA expression of *Trp53*, *Cdkn2a* and *Cdkn1a* genes normalized to *β-Actin* mRNA level in tumors of 30-week-old Delta16HER2 controls and Delta16HER2/SIRT6-OE mice (n = 4/group). (**D**) Detection of senescence-associated β-galactosidase (SA-β-Gal) activity in frozen tumors of 30-week-old Delta16HER2 and Delta16HER2/SIRT6-OE (n = 3). Quantification is expressed as % of SA-β-Gal positive area (blue) with respect to the total section area. Scale bar, 25 mm. In (**B**–**D**) ns p > 0.05, *p < 0.05, **p < 0.01, ***p < 0.001 (two-tailed unpaired *t* test). Error bars represent SD. See also Fig. S2.

To investigate SIRT6-induced effects on cell cycle progression, we carried out cell cycle analysis on primary cell cultures derived from both Delta16HER2 and Delta16HER2/SIRT6-OE tumors. Flow cytometry analysis revealed that Delta16HER2 and Delta16HER2/SIRT6-OE tumors collected at 20 weeks of age have similar cell cycle profiles (Fig. 3A**,** upper panels). However, at the final endpoint of 30 weeks of age, while Delta16HER2 cells exhibit a G2/M phase arrest (more than 50% of total population), Delta16HER2/SIRT6-OE tumors maintain the majority of cells in the G0/1 phase (Fig. 3A**,** lower panels).

These data were confirmed by western blot analysis of G1 and G1/S specific cyclins, cyclin D1 and cyclin E, respectively (Fig. 3B). As previously reported in other studies^53,54^, these cell cycle data indicate that SIRT6-OE is able to lower proliferation and mitotic rate, therefore preventing the G2/M accumulation often associated with senescence^55–57^. Accordingly, we found that Delta16HER2/SIRT6-OE tumors have a lower mRNA expression of senescence-associated genes such as *Trp53*, *Cdkn2a*, *Cdkn1a* than Delta16HER2 controls (Fig. 3C). Moreover, Delta16HER2/SIRT6-OE tumors exhibit lower senescence-associated β-galactosidase (SA-β-Gal) levels (Fig. 3D). Accordingly, Delta16HER2/SIRT6-OE tumors have also lower levels of the oxidative DNA damage marker, 8-Oxo-2’-deoxyguanosine (8-oxo-dG), when compared to controls (Fig. S2). This evidence indicates that SIRT6 protects cancer cells from the oxidative DNA damage during tumor progression.

### SIRT6 promotes stemness and self-renewal capacity of Delta16HER2 tumor cells

**Figure 4:**
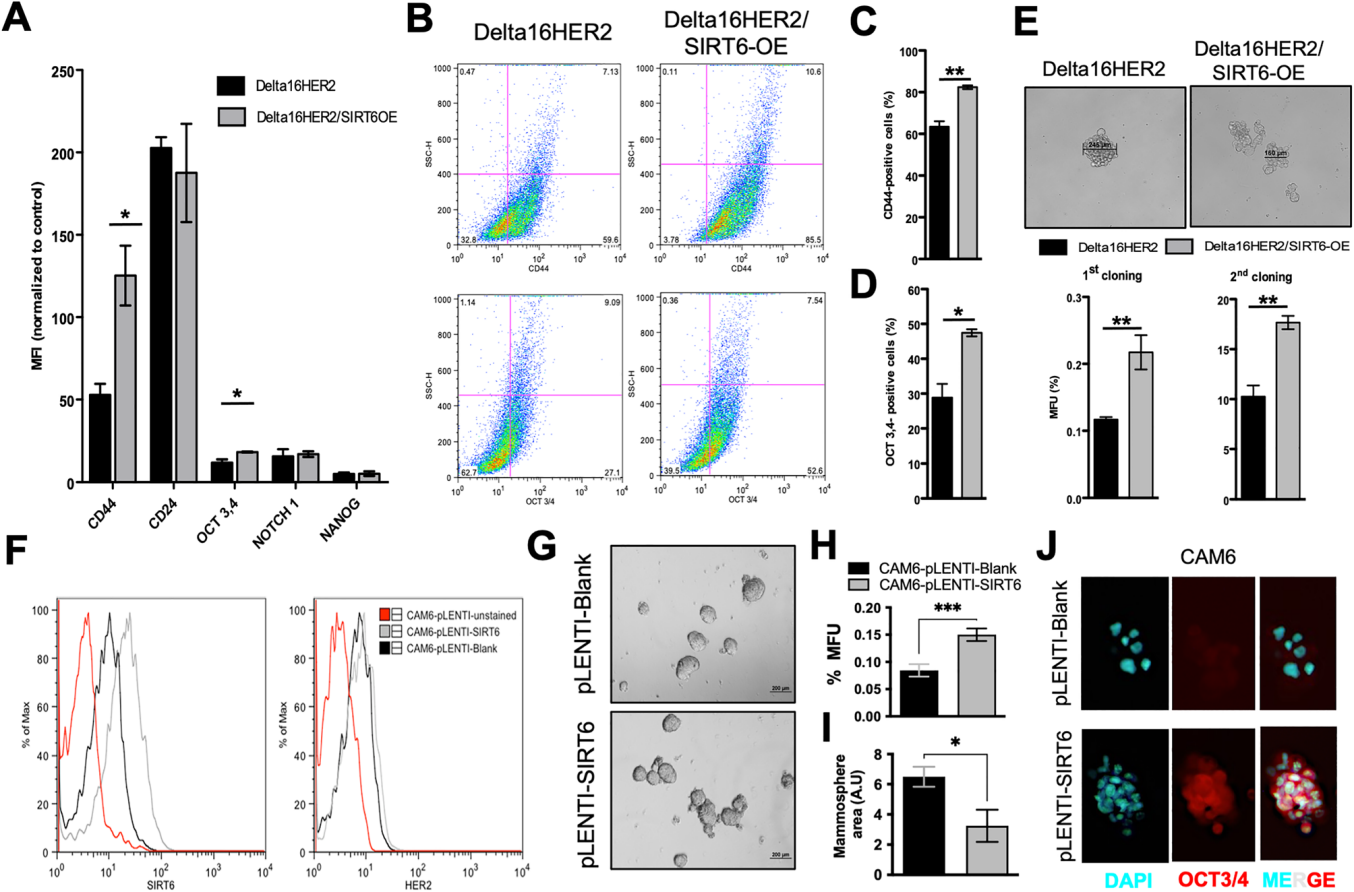
SIRT6 promotes stemness and self-renewal capacity of Delta16HER2 tumor cells. (**A**–**D**) Flow Cytometry analysis of CD44, CD24, OCT3/4, Notch1 and NANOG levels in primary tumor cells derived from 30-week-old Delta16HER2 and Delta16HER2/SIRT6-OE mice (n = 4). Quantification is expressed as median fluorescence intensity (MFI) (**A**) and as percentage of positive cells (**C** and **D**). (**E**) Representative images of mammosphere formation assay performed on primary tumor cells derived from 30-week-old Delta16HER2 and Delta16HER2/SIRT6-OE mice (n = 4, top panel). Quantification is represented for 2 serial cloning assays and is expressed as percentage of mammosphere forming units (MFU) with respect to the total number of seeded cells (bottom panel). See also Fig. S3. (**F**) Flow cytometry analysis of SIRT6 and HER2 expression in CAM6 cells stably transduced with pLENTI-Blank (empty vector) and pLENTI-SIRT6. (**G**) Mammosphere formation assay on CAM6-pLENTI-Blank and CAM6-pLENTI-SIRT6. Scale bar, 200 mm. (**H**,**I**) Quantification of mammosphere forming capacity (MFU %) and average sphere area of CAM6-pLENTI-Blank and CAM6-pLENTI-SIRT6 cells. Experiment was carried out in triplicate. (**J**) Immunofluorescence staining of OCT3/4 on CAM6-pLENTI-Blank and CAM6-pLENTI-SIRT6 mammospheres. DAPI is used for nuclei counterstaining. Magnification 40X. In (**A**,**C**–**E**,**H**,**I**) *p < 0.05, **p < 0.01, ***P < 0.001 (two-tailed unpaired t test). Error bars represent SEM.

The slow-cycling status exhibited by Delta16HER2/SIRT6-OE tumors at 30 weeks of age, prompted us to test whether this phenotype correlates with the acquisition of CSC-like features. Primary cells derived from Delta16HER2 and Delta16HER2/SIRT6-OE tumors were evaluated by flow cytometry for the expression of the CSC markers CD24, CD44, OCT3/4, NOCTH1 and NANOG. We observed a significant increase in CD44 and OCT3/4 levels in Delta16HER2/SIRT6-OE tumor bulk over Delta16HER2 both in terms of median fluorescence intensity (MFI) (Fig. 4A), and population frequency (Fig. 4B–D). Mammosphere assay confirmed that Delta16HER2/SIRT6-OE cells have higher self-renewal capacity and mammosphere formation efficiency than Delta16HER2 counterparts during two subsequent cloning procedures (Fig. 4E). Of note, these spheres recapitulate in vitro the features of Delta16HER2/SIRT6-OE tumors in vivo*,* in that they are smaller but more numerous than Delta16HER2 counterparts (Fig. S3).

The same results were achieved using the murine Delta16HER2-positive CAM6 cell line stably transfected with either empty or SIRT6 pLENTI vectors, CAM6-pLENTI-Blank and CAM6-pLENTI-SIRT6, respectively (Fig. 4F). As expected, CAM6-pLENTI-SIRT6 form more mammospheres than CAM6-pLENTI-Blank (Fig. 4G–I) consistently with high OCT3,4 expression (Fig. 4J).

### SIRT6 molds AKT, MAPK/ERK pathways redirecting Delta16HER2 cancer cells to dormancy

HER2 and Delta16HER2 promote tumorigenesis and proliferation via several downstream pathways. In particular, AKT, ERK1/2 and MAPK-p38 pathways are well-established hallmarks of proliferation and dormancy^58,59^. At 20 weeks when Delta16HER2/SIRT6-OE mice start to develop higher number of foci, an up-regulation of phospho-ERK1/2 (pERK) and down-regulation of MAPK-p38 pathways were concomitantly detected in Delta16HER2/SIRT6-OE tumors with respect to Delta16HER2 (Fig. S4A and B**,** and additional blots in Supplementary Information). By stimulating ERK1/2 activation and keeping low phospho-MAPK-p38 (pMAPK-p38) levels, Delta16HER2/SIRT6-OE tumor cells may acquire an initial proliferative advantage which later results in multiple tumor foci in vivo. Additionally, sustained AKT activation (pAKT) in Delta16HER2/SIRT6-OE tumors might synergize with ERK1/2 pathway thereby contributing to the tumorigenic switch at 20 weeks. By contrast, at 30 weeks of age, Delta16HER2/SIRT6-OE cancers displayed a completely opposite signaling, showing a high pMAPK-p38/pERK ratio which is a marker of higher tumor dormancy and quiescence (Fig. S4C–E, additional blots in Supplementary Information)^58,60,61^. Accordingly, also pAKT levels significantly decrease in Delta16HER2/SIRT6-OE at 30 weeks (Fig. S4C and D). As it regards Delta16HER2 tumors, they invariably displayed high ERK1/2 and AKT activation levels at both 20 and 30 weeks of age, which maintain constant proliferation rate over time. Moreover, MAPK-p38 pathway has been endowed with growth inhibitory properties, thus, its progressive abolishment might lead Delta16HER2 cancers first to grow and replicate quickly at early stages of tumorigenesis, while triggering senescence at later stages (Fig. S4A–D, additional blots in Supplementary Information). Interestingly, we found no significant changes in PI3K/mTOR pathway (Fig. S5). This observation suggests a disengagement of AKT cascade from PI3K in this mouse model, rather pointing toward a direct AKT regulation by SIRT6, as described in other works^26,53^.

Finally, SIRT6-OE did not significantly influence the HER2/SRC/STAT3 pathway which is the main signaling pathway downstream D16HER2 in our mouse model^27,29^ (Fig. S6).

### SIRT6 predicts poor relapse-free survival in a subset of HER2-positive breast cancer patients

To determine whether SIRT6-OE is relevant for HER2-positive breast cancer patients, we interrogated publicly available datasets using cBioPortal and GOBO databases.

**Figure 5:**
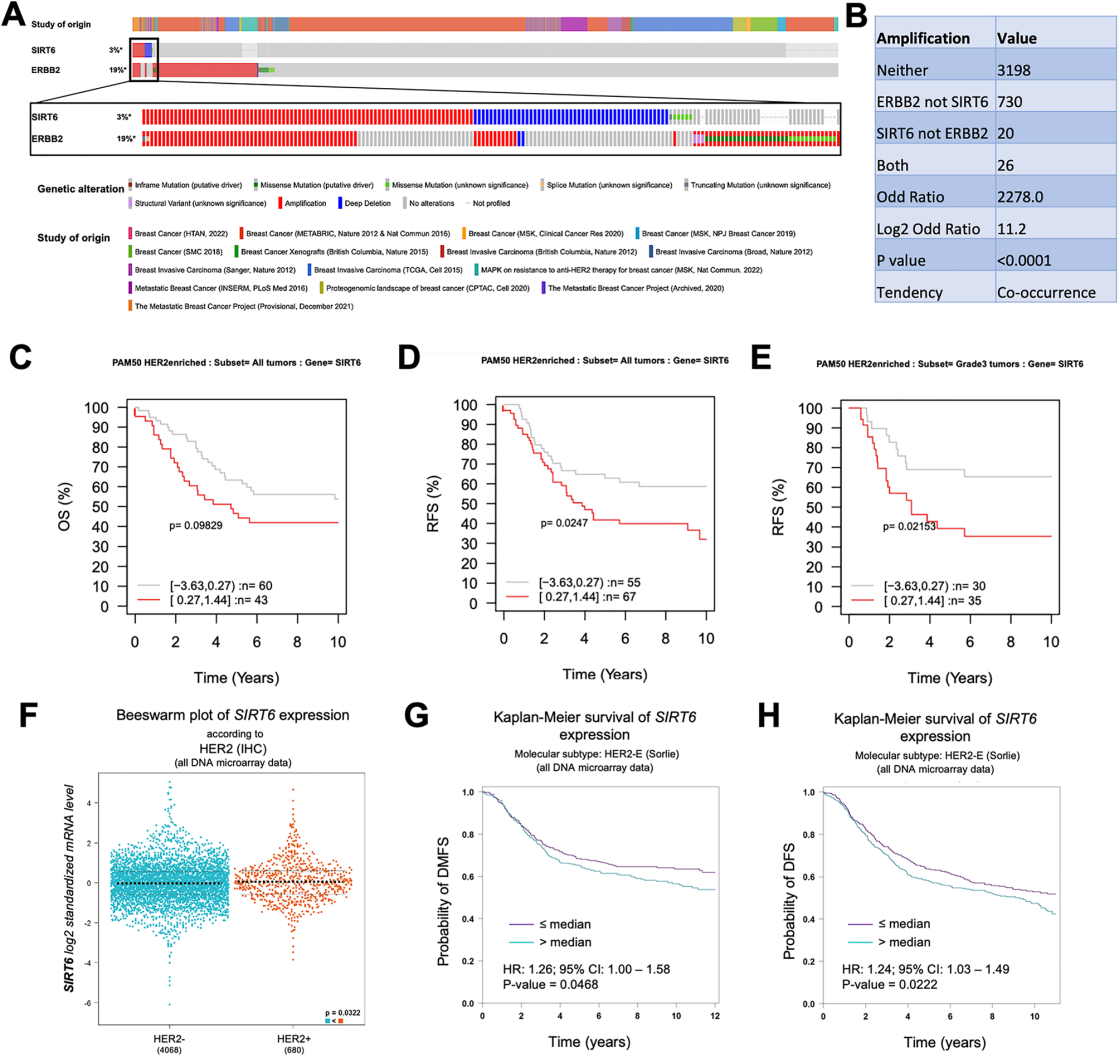
SIRT6 is amplified in a subset of HER2-positive breast cancer patients and correlates with poor relapse-free-survival. (**A**) cBioPortal data summarizing the type and frequency of alteration of *HER2* (*ERBB2*) and *SIRT6* genes in sequenced samples from publicly available invasive breast cancer studies (listed in the “study of origin” section). A total of 4860 and 4379 breast cancer patients were profiled for *ERBB2* and *SIRT6* alterations, respectively. (**B**) Co-occurrence/Mutual exclusivity analysis from cBioPortal indicating the number of patient samples that have amplification of HER2 (ERBB2) and SIRT6 alone, in both or neither genes. Odd ratio = neither*both/SIRT6 not ERBB2*ERBB2 not SIRT6. Log2 Odd Ratio > 1 indicates co-occurrence of HER2 and SIRT6 amplifications (p < 0.0001, Pearson correlation). (**C**–**E**) Kaplan–Meier plots from GOBO database using overall survival (**C**) and relapse-free survival (RFS) (**D**,**E**) as outcome in all HER2-enriched breast cancer (**D**) and in grade 3 HER2-enriched breast cancer (**E**). Data have been stratified into the two quantiles based on SIRT6 gene expression level (SIRT6_low, grey line and SIRT6_high, red line) using 10-year censoring as endpoint. (**F**) DNA microarray data from bc-GenExMiner showing SIRT6 expression in HER2- (n = 4068) and HER2 + (n = 680) breast cancer samples. (**G**,**H**) Kaplan–Meier plots from bc-GenExMiner using distant metastasis free survival (DMFS) and disease free survival (DFS) as outcomes in HER2-enriched breast cancer. Data have been stratified into the two quantiles based on SIRT6 gene expression level (SIRT6_low, purple line and SURT6_high, blue line). HR (hazard ratio) and 95% CI (confidence interval) are reported in the figure. In (**C**,**D**,**F**,**G**) *p = 0.02436, *p = 0.02143, *p = 0.0468, *p = 0.0222 (Log-rank test). In (**E**) *p = 0.0322 (Welch’s test). See also Figs. S7 and S8.

*SIRT6* was found to be altered in 138/4379 profiled patients. Out of these 138, 46 harbor *SIRT6* gene amplifications. On the other hand, *ERBB2 (HER2)* is altered in 908/4860 profiled patients, and 756 of the 908 altered have *ERBB2* gene amplification. Of note, a cohort of 26 invasive breast cancer patients, which represents about 18.8% and 3.4% of the total *SIRT6* and *ERBB2* gene amplifications, respectively, harbors a concomitant gene amplification of both *SIRT6* and *ERBB2* (Fig. 5A).

In general, *SIRT6* and *HER2* have a significant tendency to be concurrently amplified (p < 0.001, Fig. 5B).

However, the available amount of info about survival for these patients is limited in cBioPortal and does not allow for a proper comparison between *SIRT6* and *HER2* co-amplifications and *HER2* amplification only. To solve this issue, we derived Kaplan–Meier survival plots from GOBO database^62^, stratifying the patients according to *SIRT6* expression. High *SIRT6* expression (red line) correlates with a worse overall survival (OS) (although not significant, Log-rank test, p = 0.09829, Fig. 5C) and predicts a significantly poorer relapse-free survival (RFS) for patients with HER2-enriched tumors (Log-rank test, p = 0.0247, Fig. 5D).

Noteworthy, within the HER2-enriched tumors, patients with grade 3 tumors and high *SIRT6* expression (red line, n = 35) have a significant lower probability of RFS (Log-rank test, p = 0.02153) with respect to grade 3 tumors with low SIRT6 expression (grey line, n = 30, Fig. 5E). To confirm these results in a larger cohort of patients, we analyzed the DNA microarray data of 4748 breast cancer patients using the statistical miner bc-GenExMiner^63,64^. We found that HER2-positive breast cancers (n = 680) have a higher median *SIRT6* expression (p = 0.0322) than HER2-negative tumors (n = 4068, Fig. 5F). Moreover, within the HER2-enriched tumors, high *SIRT6* expression significantly correlates with lower probability of both distant metastasis-free survival (DMFS, n = 827, p = 0.0468) and disease-free survival (DFS, n = 1004, p = 0.0222) than tumors with low *SIRT6* (Fig. 5G and H).

These data validate our results obtained in Delta16HER2/SIRT6-OE mice indicating that SIRT6-OE predicts poor prognosis in HER2-positive breast cancer, with high risk of relapse and metastasis.

This outcome is highly specific for HER2-positive breast cancer, and it is independent of the estrogen receptor (ER) status. Indeed, high SIRT6 expression correlates with a worse prognosis in both ER + and ER- HER2-enriched breast cancer cases (Fig. S7A and B). In addition, *SIRT6* expression does not have any prognostic value for luminal A breast cancer patients (ER + , PR +) nor in terms of RFS or DMFS (Fig. S7C). Finally, since SIRT6-OE was reported to have anti-tumor effects in PI3K-dependent basal breast tumors^24^, we interrogated the same databases for alterations in *SIRT6* and *PIK3CA* genes and how *SIRT6* expression predicts the RFS of basal-like tumors (Fig. S8). *PIK3CA* is altered in 1847/4860 breast cancer patients. Within the *PIK3CA-*altered cases 1555 patients (about 84% of the total *PIK3CA* alterations) present either gene amplification or activating mutations of *PIK3CA* (Fig. S8A)*.* In this group, 8 patients present a concomitant deep deletion in *SIRT6.* Though at the limit of significance (p = 0.046), deep deletion of *SIRT6* tends to co-occur with *PIK3CA* amplification/activating mutations (Fig. S8B). Accordingly, Kaplan–Meier plots from GOBO show that basal-like breast cancer patients with high expression of *SIRT6* (red line, n = 70) have better prognosis in terms of RFS (Fig. S8C, Log-rank test, p = 0.04656) than patients expressing low levels of *SIRT6* (grey line, n = 73). The same trend, although not significant, is observed for DMFS (Fig. S8D, Log-rank test, p = 0.53321). This outcome indicates that SIRT6 has opposite effects in different breast cancer subtypes, in line with the previously reported anti-tumor effect of SIRT6-OE in PI3K-dependent basal tumors^24^.

### TBX3 is a SIRT6 target in Delta16HER2/SIRT6-OE tumors and HER2-positive breast cancer patients

SIRT6 can regulate gene expression and chromatin integrity via deacetylation of H3K9ac^9,16,65,66^. To gain mechanistic insights, we performed H3K9ac ChIP-seq and RNA-seq on tumors from Delta16HER2/SIRT6-OE and Delta16HER2 mice, harvested at 20 weeks of age. As the phenotypic changes start to occur during this timeframe, we reasoned that this endpoint would be the most informative.

**Figure 6:**
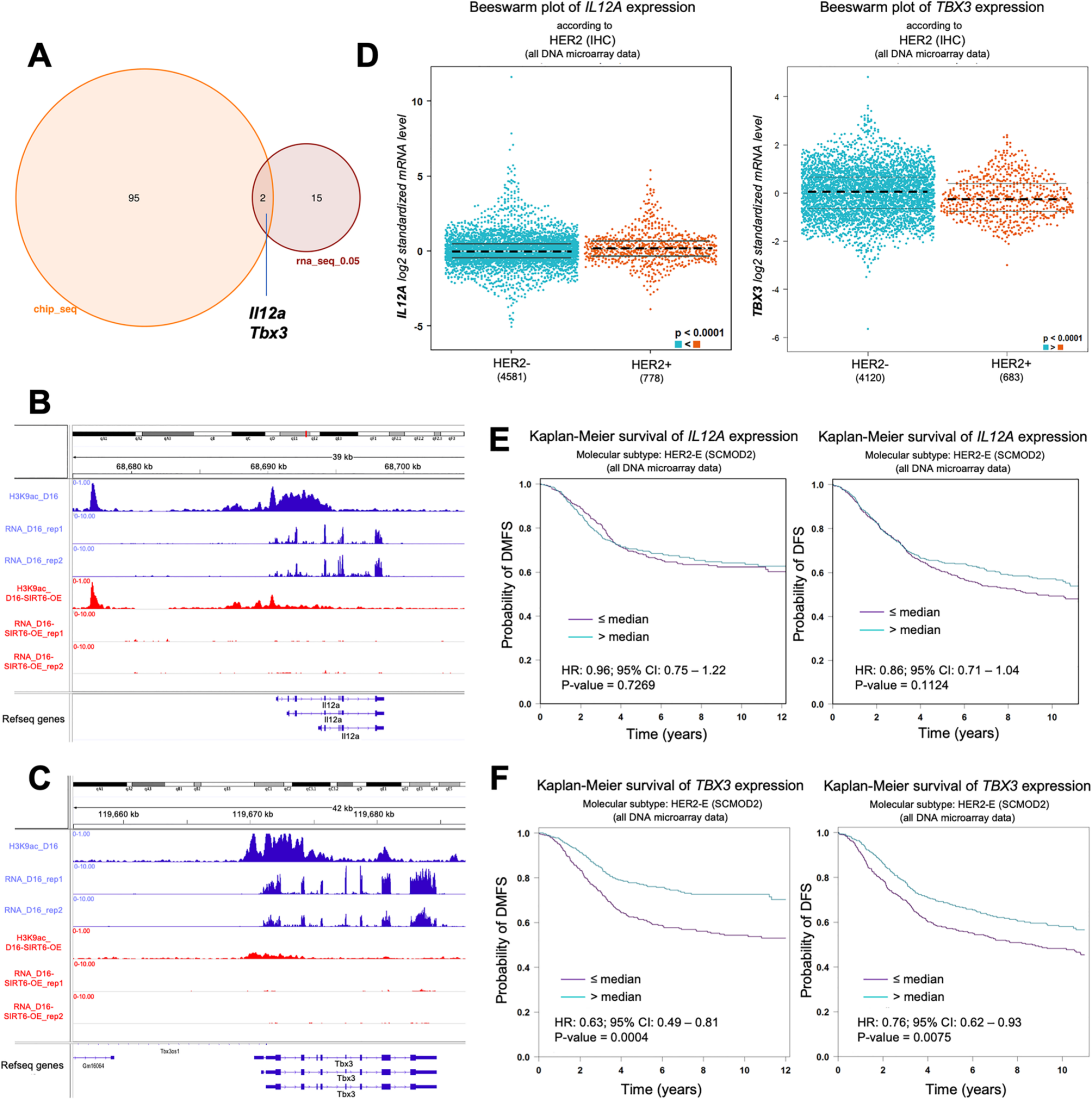
ChIP- and RNA-seq identify *TBX3* as a SIRT6 target and a prognostic marker in HER2 + breast cancer. (**A**) Venn diagram representing the genes that are differentially expressed and differentially bound by H3K9ac (cutoff FDR ≤ 0.05) in tumors from Delta16HER2/SIRT6-OE versus Delta16HER2 controls (20 weeks of age, n = 2 for RNA-seq and n = 2–3 for ChIP-seq). (**B**,**C**) ChIP and RNA-seq tracks showing H3K9ac level and expression for *Il12a* and *Tbx3* genes in Delta16HER2 (blue) versus Delta16HER2/SIRT6-OE (red) tumors (at 20 weeks of age). (**D**) Bee swarm plots computed in bc-GenExMiner showing the expression levels of *IL12A* and *TBX3* in HER2–/ + breast cancers. (**E**,**F**) Kaplan–Meier plots from bc-GenExMiner using distant metastasis free survival (DMFS) and disease free survival (DFS) as outcomes in HER2-enriched breast cancer. Data have been stratified into the two quantiles based on *IL12A* gene expression level ((**E**), IL12A_low, purple line and IL12A_high, blue line) and *TBX3* gene expression level ((**F**), TBX3_low, purple line and TBX3_high, blue line). HR (hazard ratio) and 95% CI (confidence interval) are reported in the figure. In (**D**) ****P < 0.0001 (Welch’s test). In (**E**,**F**) ns, p = 0.1124; ns, p = 0.7269; ***p = 0.0004, **p = 0.0075 (Log-rank test). See also Figs. S9 and S10.

We found 17 genes to be differentially expressed and 97 to be differentially bound by H3K9Ac between Delta16HER2/SIRT6-OE and Delta16HER2 tumors (Fig. 6A). We are cognizant that the small sample size (n = 2–3) might have contributed to this outcome and it’s a limitation of our study. However, the very low amount of genes that are differentially expressed and differentially bound by H3K9ac might also indicate that SIRT6-OE acts via H3K9Ac only on specific loci depending on the context and the availability of co-binding partners, as previously reported in other studies^67^. This explains also why SIRT6-OE does not induce a significant visible change in the total protein level of H3K9ac (Fig. S9). Although very few, the differentially expressed genes are involved in pathways and signatures that are consistent with the phenotypes we observed upon SIRT6-OE. For instance, upregulated genes significantly enriched for the binding of Yamanaka factors including OCT3,4 (*alias* POU5F1), and other stem cell regulators such as TCF-4 (*alias* TCF7L2) (Fig. S10A). In addition, the top significant hallmark for these genes is “epithelial to mesenchymal transition” (EMT) which is consistent with the increased metastasis and mobility/invasion of SIRT6-OE tumor cells (Fig. S10A and Fig. 2).

The downregulated genes in SIRT6-OE tumors significantly overlap with the proteomic signature upregulated in cells treated with entinostat, a histone deacetylase (HDAC) drug inhibitor used as breast cancer therapy^68^, confirming that this gene set is indeed regulated by histone acetylation in breast cancer (Fig. S10B). These genes are also predicted to be downregulated upon MAPK14 (*alias* MAPK-P38) knockdown (Fig. S10B), overlapping with the decrease of pMAPK-p38 signaling reported in Fig. S4A,B. By comparing the significant hits (with FDR ≤ 0.05) obtained via ChIP-seq and RNA-seq, we found that *Il12a* and *Tbx3* were the only two genes to be concomitantly less bound to H3K9ac and less expressed in Delta16HER2/SIRT6-OE than in the Delta16HER2 tumors (Fig. 6A–C). *Il12a* encodes for the p35 subunit of interleukin 12 (Il12), a cytokine involved in the generation of an inflammatory tumor microenvironment (TME) and is critical in eliciting a productive antitumor immune response by acting on T and NK cells^69,70^. IL12 has been proposed to act as a tumor suppressor, enhancing the efficacy of the immunotherapy in some cancers^71^. However, when we queried bc-GenExMiner, even though we found that *IL12A* is more expressed in HER2-positive tumors (n = 778) than in HER2-negative ones (n = 4581) (Fig. 6D), *IL12A* expression level is not a significant predictive marker for DFS or DMFS in HER-positive breast cancer patients (Fig. 6E, high *versus* low *IL12A* p = 0.1124 and p = 7269).

On the other hand, *TBX3,* which encodes for the T-box transcription factor 3, is significantly more expressed in HER2-positive (n = 4120) than in HER2-negative (n = 683) breast cancers (Fig. 6D) and lower *TBX3* expression is predictive of poor DMFS and DFS in HER2-positive breast cancer patients (Fig. 6F, high *versus* low *TBX3* p = 0.0004 and p = 0.0075). As shown for SIRT6-OE, *TBX3* low expression is predictive of poor prognosis specifically in HER2-positive breast cancer patients. Indeed, *TBX3* expression is significantly lower in basal-like and triple negative breast cancer (TNBC) than in the other subtypes (Fig. S11A). Also, higher expression of *TBX3* significantly correlates with better DMFS and DFS in basal-like breast cancer patients (Fig. S11B and C).

### SIRT6-OE induces loss of TBX3 in Delta16HER2-positive and HER2-positive breast cancer models

**Figure 7:**
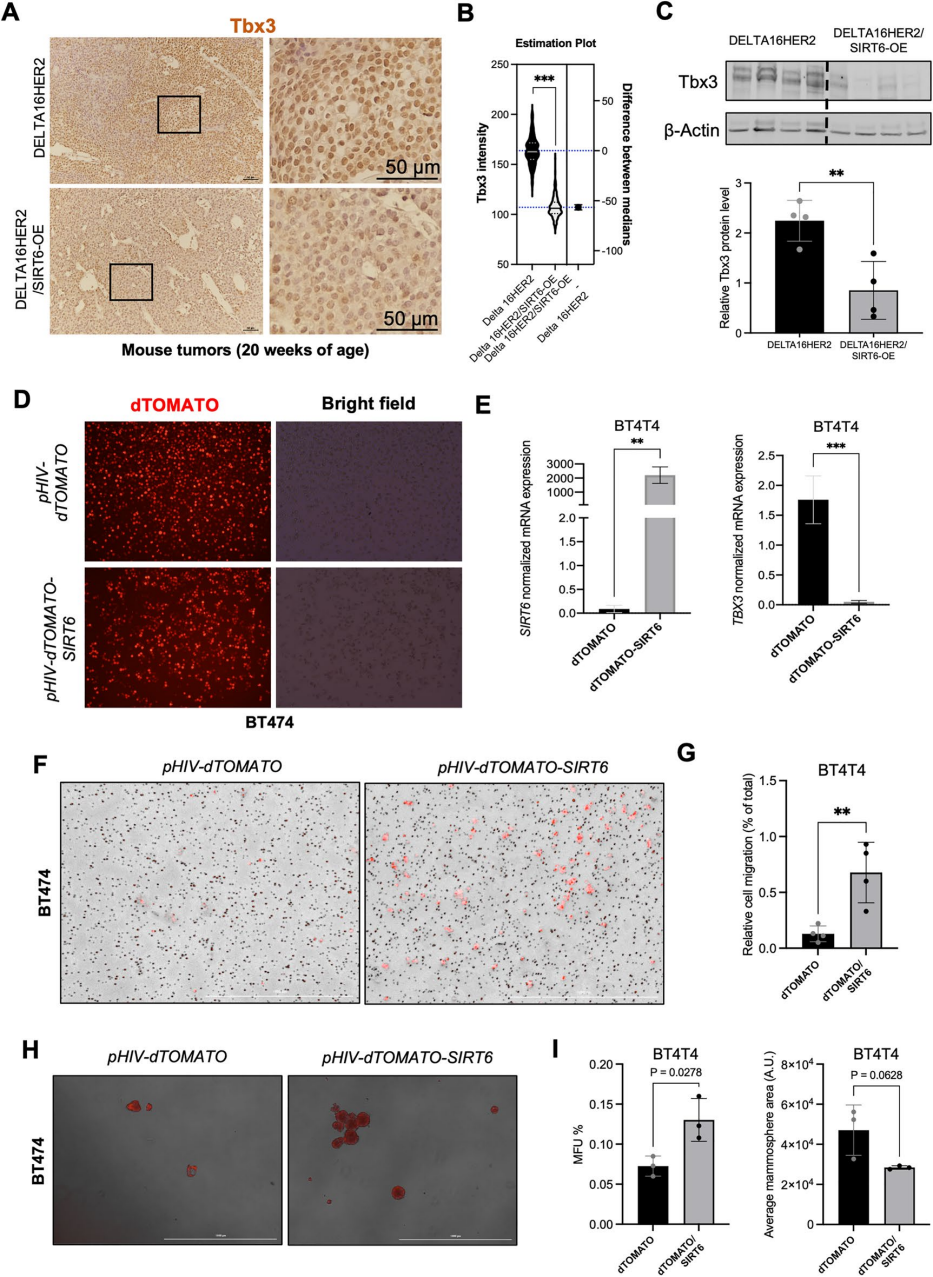
SIRT6-OE induces loss of TBX3 and aggressiveness in both mouse and human models of Delta16HER2/HER2 + . (**A**) Representative pictures and (**B**) quantification for Tbx3 IHC in the indicated mouse tumors. (**C**) Immunoblot of Tbx3 in mouse tumors of the indicated genotype (20 weeks, n = 4 per group). (**D**) Representative pictures of the human BT474 cell line transfected with either pHIV-dTomato or pHIV-SIRT6-dTomato. (**E**) Real-time PCR for *SIRT6* and *TBX3* expression in sorted BT474 transfected as indicated. Not transfected HEK293 were used as control sample and *TBP* as housekeeping gene. (**F**) Representative pictures and (**G**) quantification of the Transwell migration assay of the human BT474 cell line transfected with either pHIV-dTomato or pHIV-SIRT6-dTomato. (**H**) Representative pictures and (**I**) number and area quantification of pHIV-dTomato and pHIV-SIRT6-dTomato BT474 mammospheres. MFU% = % of mammary forming units over total number of seeded cells. *A.U*. arbitrary unit. Bars indicate mean ± SD. In (**B**) ***p = 0.0009; (**C**) **p = 0.0077; (**D**) **p = 0.0014, ***p = 0.0003; (**G**) **p = 0.0077 and (**I**) statistics indicates unpaired two-tailed *t* test.

To validate our sequencing results we performed Tbx3 IHC and western blot on tumors harvested from Delta16HER2/SIRT6-OE and Delta16HER2 mice (Fig. 7A–C). Consistent with our RNA-seq and ChIP-seq data indicating that SIRT6 downregulates the expression of *Tbx3*, Tbx3 protein levels were significantly lower in Delta16HER2/SIRT6-OE tumors than in Delta16HER2 ones (Fig. 7A–C).

To confirm whether SIRT6-OE is sufficient to induce *TBX3* downregulation, we ectopically overexpressed *SIRT6* in the human HER2 + /Delta16HER2 + breast cancer cell line BT474. As expected, SIRT6-OE significantly decreased *TBX3* transcript levels in this cell line as well (Fig. 7D,E). Moreover, consistent with the results obtained in our mouse models, the human BT474 cells with SIRT6-OE and low TBX3 display higher migratory and self-renewal capabilities than and the wild-type counterparts (Fig. 7F–I). Similar results were obtained overexpressing *SIRT6* in BCM-4888 cells, a patient-derived model of HER2 + /ER + breast cancer^72^ (Fig. S12).

### *TBX3* loss-of-function mimics *SIRT6*-*OE* in vitro and predicts poor survival of HER2-positive breast cancer patients

**Figure 8:**
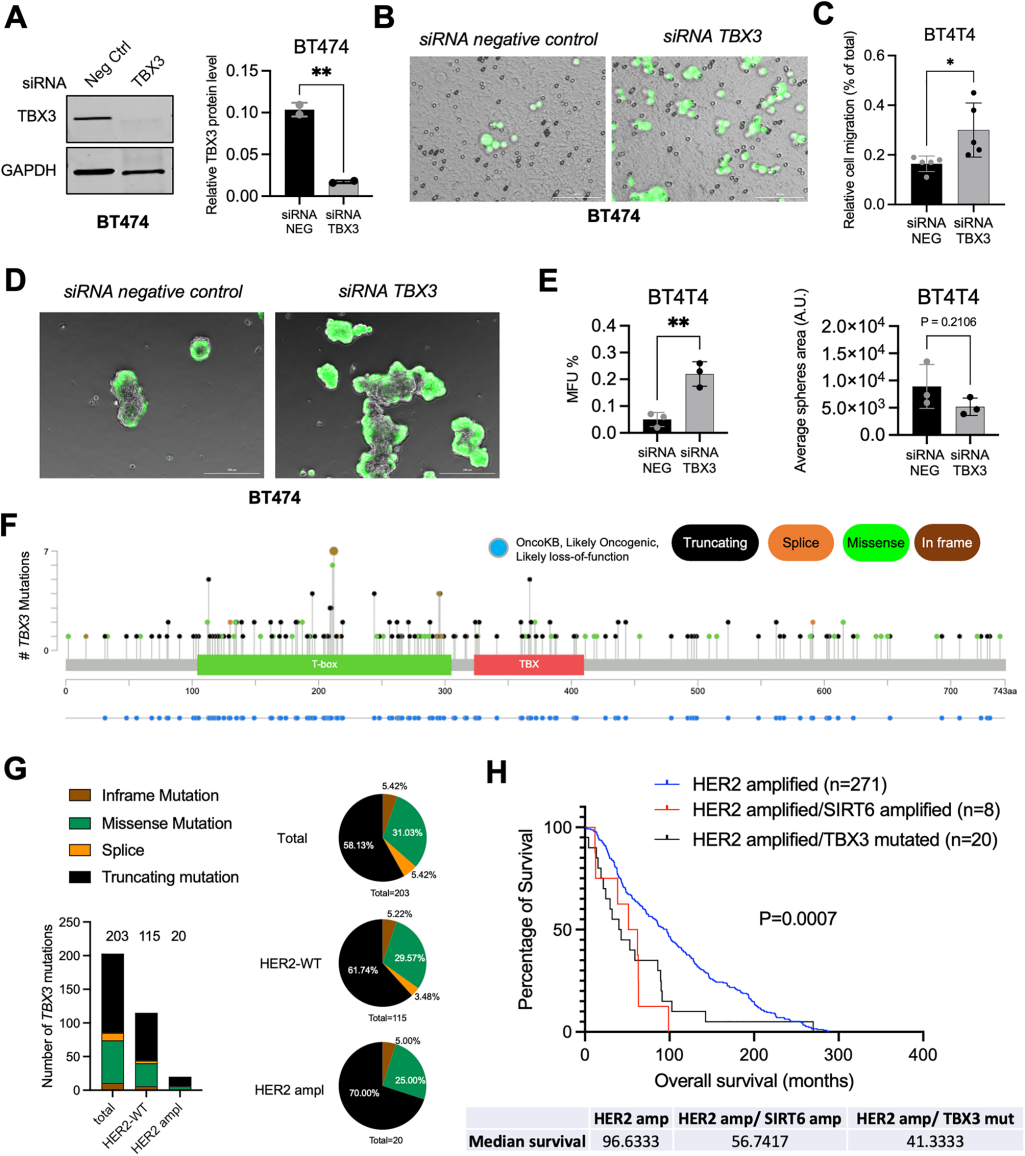
Loss of *TBX3* mimics *SIRT6*-OE in vitro and predicts poor survival of HER2-positive breast cancer patients. (**A**) Immunoblot and quantification of TBX3 and GAPDH in lysates of BT474 cell line transfected with the indicated siRNA (48 h post transfection, n = 2 independent replicates). (**B**) Representative pictures and (**C**) quantification of the Transwell migration assay of the human BT474 cell line transfected as indicated (n = 5). (**D**) Representative pictures and (**E**) number and area quantification of siRNA control and siRNA *TBX3* BT474 mammospheres (n = 3). MFU% = % of mammary forming units over total number of seeded cells. *A.U*.  arbitrary unit. Bars indicate mean ± SD. See also Fig. S12. (**F**) Lollipop graph from cBioPortal showing mutations of the *TBX3* gene in breast cancer patients (n = 338). Y axis represents the number of mutations while X axis indicates the correspondent amino acid position. OncoKB tracks in blue indicate those mutations that are likely-oncogenic and likely loss-of-function. Types of mutation are color-coded in the figure legend. (**G**) Summary of total number and frequency of *TBX3* mutations in all, HER2-WT and HER2 amplified breast cancer subsets. Pie charts indicate the frequency of the different types of *TBX3* mutations over total number of *TBX3* mutations in each subset. (**H**) Kaplan–Meier plots from cBioPortal showing the overall survival of patients with *HER2* amplified alone (n = 271), and those with concomitant *SIRT6* amplification (n = 8) or *TBX3* loss-of-function mutations (n = 20)*.* P = 0.0007 (Log-rank Mantel-Cox test). In (**A**) **p = 0.0047, (**C**) *p = 0.0282, (**E**) **p = 0.0051 and ns, p = 0.2106 indicates unpaired two-tailed *t* test.

Our data indicate that SIRT6-OE suppresses the expression of *TBX3.* To test whether direct *TBX3* loss is sufficient to mimic SIRT6-OE, we induced siRNA-mediated knockdown of *TBX3* in both BT474 (Fig. 8A–E) and BCM-4888 (Fig. S13) human breast cancer cells. Of note, *TBX3* knockdown recapitulates the effects of SIRT6-OE in both models, significantly increasing cell invasion (Fig. 8B,C and Fig. S12B,C) and mammosphere formation (Fig. 8D,E and Fig. S13D,E).

To test whether *TBX3* acts as a tumor-suppressor in human patients, we looked for putative oncogenic mutations of *TBX3* in cBioPortal. Noteworthy, we found that in breast cancer patients *TBX3* has many mutations, the majority of which are truncating and missense mutations classified as likely oncogenic and likely loss-of-function events, suggesting that *TBX3* acts as a tumor suppressor in breast cancer (Fig. 8F and G). This is particularly true for HER2-amplified breast cancers, in which almost all *TBX3* mutations are loss-of function ones (Fig. 8G, 70% truncating and 35% missense). To investigate whether *TBX3* loss-of-function mutations predict a worse survival in HER2-amplified patients and if they phenocopy *SIRT6* amplification, we analyzed the patients with available OS information in cBioPortal (Fig. 8H). In the context of HER2-amplified breast cancer, either *TBX3* loss-of-function or *SIRT6* amplification predicts a worse survival than patients with no alterations in *SIRT6* and *TBX3* (Fig. 8H, Log-rank P = 0.0007). This evidence indicates that *TBX3* has a tumor suppression function and its loss phenocopies *SIRT6* amplification in HER2-positive breast cancer.

## Discussion

Our work suggests that SIRT6 acts as a tumor oncogene in HER2-positive breast cancer. Indeed, even if SIRT6-OE significantly postponed tumor onset in Delta16HER2/SIRT6-OE mice, around the 20th week of age Delta16HER2/SIRT6-OE animals started to develop more tumor masses. Moreover, Delta16HER2/SIRT6-OE mice were found to suffer from more and larger metastatic lesions than Delta16HER2 animals. At the molecular level, we observed that SIRT6-OE does not modulate SRC/STAT3 pathway, that is the main molecular axis in the Delta16HER2 model, but rather influences AKT, ERK1,2 and MAPK-p38 signaling cascades. At later stages of tumorigenesis, SIRT6-OE correlates with the suppression of AKT pathway and with a high pMAPK-p38/pERK ratio. This signaling profile is consistent with the concept the “G0-like” progeny that can arise inside tumors by asymmetric division^73^. Such cell population renders tumors less sensitive to stress and hypoxia, it is usually enriched after chemotherapy, and contributes to relapse and treatment failure^73,74^. In this context, the multifocal morphology displayed by Delta16HER2/SIRT6-OE tumors might function as a strategy to evade senescence as well. Furthermore, the MAPK-p38^high^/ERK1,2^low^ paradigm we reported, has been previously associated with G0/1 arrest and tumor dormancy^61,75^. Consistently, SIRT6-OE maintains the majority of Delta16HER2/SIRT6-OE tumor cells in G0/1 phase, while Delta16HER2 tumors show G2/M accumulation typical of highly proliferating tumors. SIRT6 capacity to participate in DNA repair^76–78^, might contribute as well to this phenotype, preventing the arrest in G2 and the senescence of Delta16HER2/SIRT6-OE tumors. Accordingly, we found that SIRT6-OE represses the transcription of cell cycle and senescence guardians such as p53, p21 and p16^79–81^ in Delta16HER2/SIRT6-OE tumors, further suggesting its anti-senescence and pro-quiescence action in this model.

The Delta16HER2/SIRT6-OE tumors exhibit a more pronounced expression of CD44 and OCT3/4, two specific markers for breast cancer stem cells (BCSCs)^82–84^, a higher mammosphere-forming ability than controls in anchorage-independent conditions, and stem cell-like transcriptional signatures. These findings indicate an enrichment of the stem cell compartment in Delta16HER2/SIRT6-OE mammary carcinomas.

Our findings are apparently in contrast with what has been found in basal-like tumors with PI3K activation, in which SIRT-OE exerts anti-tumor effects by suppressing the CSCs^24^. However, data from cBioPortal, GOBO and bc-GenExMiner confirmed that SIRT6-OE is indeed predictive of good and poor prognosis in basal-like and HER2-positive breast cancer patients, respectively. This evidence strengthens the concept of a context-dependent pro- or anti-tumor effect of SIRT6. As Kugel and Mostoslavsky previously suggested^85^, it could be also a matter of timing. Perhaps, *SIRT6* expression is often downregulated in early tumor formation contributing to the increased genomic instability and pro-tumor reprogramming. However, at later stages, enhanced SIRT6 activity may protect cancer cells against further mutagenesis and oxidative stress which could negatively impact tumor growth^85^. This description perfectly fits not only our preclinical data, but also what we observed in patients, where high expression of *SIRT6* correlates with a significantly worse prognosis of HER2-positive breast cancer patients, in particular those with grade 3 tumors. However, why this time/age-dependent effect of SIRT6 on tumorigenesis applies only to some tumor subtypes remains to be clarified. In this regard, SIRT6 co-factors or targets may contribute to the different outcomes observed in different tumor types/subtypes. For instance, we identified *TBX3* as a likely target downregulated by SIRT6 in our in vivo and in vitro models.

As reported for SIRT6^86^, also TBX3 has been described to regulate a plethora of genes, including CSC and tumor progression markers, in a tissue-specific manner^87–89^.

In this regard, even if the expression of *TBX3* and *SIRT6* is inversely correlated in both basal-like and HER2-positive breast cancer patients, their paired expression levels have opposite predictive meanings in these two cancer subtypes. Indeed, if on the one hand the concomitant high *SIRT6* and low *TBX3* expression predicts better prognosis in basal-like breast cancer, on the other, it is indicative of poor prognosis in HER2-positive breast cancer patients. The relevance of these results is reinforced by the fact that loss-of-function mutations of *TBX3* are found in 5.64% of all the breast carcinoma patients (https://www.mycancergenome.org/content/gene/tbx3/#ref-3)^90^ and we showed that they correlate with a significant lower overall survival of HER2-positive breast cancer patients. Accordingly, the silencing of *TBX3* promotes migration and self-renewal of both human Delta16HER2- and HER2-positive breast cancer cells in vitro.

Altogether, these data suggest that *TBX3* suppression or loss mimics SIRT6-OE and amplification in HER2-positive breast cancer and demands for further future specific investigations about their interaction/co-regulation and their role in therapy resistance, relapse and lung metastasis.

The low amount of genes that were differentially expressed and differentially bound by H3K9Ac suggests that SIRT6-OE can deacetylate H3K9Ac only on specific loci depending on the cellular context and the availability of co-binding partners and regulators^67^. Even though this behavior can explain why SIRT6 has opposite roles in different cancer subtypes, we are cognizant that SIRT6 can also act directly and indirectly via mechanisms that are independent of its histone deacetylase activity^4,5,23–25,85,91,92^ and future studies will be focused on investigating additional mechanisms mediated by SIRT6-OE in our models.

Also, even though our conclusions were supported by functional validation in preclinical models and human datasets, we recognize that the low number of samples used for the ChIP- and RNA-seq experiments represents a limitation of the present work. Future studies including a larger sample size and patient-derived samples will be fine-tuning our pilot observations.

Despite the need for more work to help refine these aspects, to our knowledge this is the first study investigating the effect of SIRT6-OE on Delta16HER2-dependent mammary tumorigenesis and reporting a breast cancer subtype-specific, pro-tumorigenic role for this sirtuin.

Therefore, our results provide evidence that SIRT6-OE is a marker of poor prognosis in HER2-positive breast cancer and that SIRT6 inhibition might be beneficial in this cancer type. As several novel small molecule activators and inhibitors of SIRT6 are becoming available^21,93–95^, future pharmacological studies will be of pivotal importance to elucidate the role of SIRT6 and will help identify suitable breast cancer subtype-specific interventions.

## Methods

### Human breast cancer datasets

Publicly available gene expression data on human breast tumors were obtained via GOBO^62^, cBioPortal^96,97^ and bc-GenExMiner databases^63,64^.

### Animals

All experiments were performed in accordance with ARRIVE guidelines and the directive 2010/63/EU on the protection of animals used for scientific purposes. All procedures were approved by the Ethic Committee on Animal Use of the University of Camerino (protocol number 14/2012).

Mice were housed under controlled temperature (20 °C) and circadian cycle (12-h light/12-h dark). The animals were fed on chow diet and water ad libitum. Female mice have been used for all experiments. All animals were humanely euthanized under gradual CO_2_ exposure followed by cervical dislocation.

### Generation and in vivo monitoring of Delta16HER2/SIRT6-OE mice

Delta16HER2 male mice^27^ were bred to Sirt6BAC females^52^ to obtain Delta16HER2/SIRT6-OE females. Since tumorigenesis can negatively affect lactation, we avoid using Delta16HER2 females for mating. The offspring was characterized by genotyping of tail biopsies. Delta16HER2/Sirt6wt female mice (later regarded as Delta16HER2) obtained from the same mating were used as controls.

Starting at 8 weeks of age, both Delta16HER2/SIRT6-OE and Delta16HER2 females were weekly monitored for tumor formation by palpation. Masses greater than 1.5 mm in diameter were regarded as tumors. Tumor growth was measured by means of an electronic caliper. Tumor curves were derived for each single palpable tumor mass in every mouse. Then the average tumor volume per mouse was calculated per each mouse in both group.

Because hormone sensitive tumors such as breast cancer have been reported to be influenced by estrous cycle even when they are surgically removed^98,99^, hormone status was assessed by vaginal smear starting a week before the experimental endpoint (20 or 30 weeks of age). All mice were euthanized on the second day of diestrus. This procedure has been carried out in compliance with OECD guidelines for preparation, reading and reporting of vaginal smears (http://www.oecd.org/chemicalsafety/testing/40581357.pdf).

### Generation of Delta16HER2/SIRT6-OE/Sirt6^–/–^ mice

Delta16HER2/SIRT6-OE/Sirt6^–/–^ mice harboring both Delta16HER2 and Sirt6BAC, but homozygous for the Sirt6wt null allele (Sirt6^–/–^) were generated as additional control group. The breeding was performed as hereafter summarized.

*F1a*. Sirt6BAC females (SIRT6-OE) bred to males heterozygous for the Sirt6wt null allele (Sirt6^+/–^)^1^ to obtain SIRT6-OE/Sirt6^+/–^ females.

*F1b*. Females heterozygous for the Sirt6wt null allele (Sirt6^+/–^) bred to Delta16HER2 males to get Delta16HER2/Sirt6^+/–^ males.

*F2*. SIRT6-OE/Sirt6^+/–^ females bred to Delta16HER2/Sirt6^+/–^ males to obtain Delta16HER2/SIRT6-OE/Sirt6^–/–^ females.

Copy number of Sirt6wt sequences was determined using *Escherichia coli* ß-galactosidase Mr00529369_cn system (Applied Biosystems) as previously reported^52^. In vivo monitoring and genotyping for Sirt6BAC and Delta16HER2 were performed as described in the previous paragraph.

### mRNA extraction and quantitative real-time PCR (qRT-PCR)

Total RNA was extracted from liquid nitrogen cryopreserved mouse tumors or from human cell cultures using TRIzol reagent (Life Technologies). RNA was quantified by measuring 260 nm absorbance via NanoDrop 1000 spectrophotometer (Thermo Scientific). RNA purity was considered good with A_260_/A_280_ ratio ≥ 2.0 and A_260_/A_230_ ratio ≥ 1.7. 2 µg of RNA was reverse-transcribed using the High-Capacity cDNA Reverse Transcription Kit (Applied Biosystems). SYBR Premix Ex Taq (Tli RNaseH Plus) reagent (TaKaRa) was used for qRT-PCR analysis. Annealing/extension temperature was optimized taking into consideration the melting temperature of the different primers listed in Table 1. In each PCR, ß-actin or TBP were used as housekeeping genes for mouse and human targets, respectively. Standard curves for target and housekeeping genes were included to evaluate reaction efficiency. Experiments were performed at least in triplicates. A 2-step-amplification program was carried out on Bio-Rad iCycler Thermal Cycler with iQ5 Multicolor Real-Time PCR Detection System.

**Table 1:** qRT-PCR primer list.

Target	Species	Primer sequence (forward and reverse)	Amplicon (bp)
Sirt1	Mouse	5′-AGCAACATCTCATGATTGGCACCG-3′	102
		5′-TCTGCCACAGCGTCATATCATCCA-3′	
Sirt2	Mouse	5′-ACGCAGAACATAGACACGCTGGAA-3′	88
		5′-AGTGTGATGTGTAGAAGGTGCCGT-3′	
Sirt3	Mouse	5′-CGGCTCTATACACAGAACATCGA-3′	75
		5′-GTGGGCTTCAACCAGCTTTG-3′	
Sirt4	Mouse	5′-GACAAGGTTGACTTTGTGCAC-3′	211
		5′-TTAAAGGCAGCAACTCTCCAC-3′	
Sirt5	Mouse	5′-TATAGGAGTCCGATCTGCCCAGC-3′	134
		5′-ACGTGAGGTCGCAGCAAGCCTCC-3′	
Sirt6	Mouse	5′-GTCTGGTCATTGTCAACCTGCAAC-3′	94
		5′-ATGAGTCTGCACATCACCTCATCC-3′	
SIRT6	Human	5′-CCCACGGAGTCTGGACCAT-3′	194
		5′-CTCTGCCAGTTTGTCCCTG-3′	
Sirt7	Mouse	5′-GTTTGCATGAGCAAAAGCTG-3′	136
		5′-ATGCAGGAGGTGCAGACTTC-3′	
Delta16HER2	Human	5′-CACCCACTCCCCTCTGAC-3′	158
		5′-GCTCCACCAGCTCCGTTTCCTG-3′	
Cdkn1a	Mouse	5′-AGACCTGTGAAGACAGGAATGGTC-3′	124
		5′- AGCAGATCACCAGATTAACCCTCC-3′	
Cdkn2a	Mouse	5′-CATCTGGAGCAGCATGGAGTC-3′	155
		5′-CGTTGCCCATCATCATCACCT-3′	
Trp53	Mouse	5′-TGTTATGTGCACGTACTCTCCTCC-′3	142
		5′-GTGCTGTGACTTCTTGTAGATGGC-′3	
ß-actin	Mouse	5′-CAAGGCCAACCGCGAGAAGAT-3′	216
		5′-GTCCCGGCCAGCCAGGTCCAG-3′	
ß-actin_bis	Mouse	5′-CAGGCATTGTGATGGACTCCGG-3′	100
		5′-CCAGCCAGGTCCAGACGCAG-3′	
TBX3	Human	5′-CCCGGTTCCACATTGTAAGAG-3′	104
		5′-GTATGCAGTCACAGCGATGAAT-3′	
TBP	Human	5′-GAGCCAAGAGTGAAGAACAGTC-3′	116
		5′-GCTCCCCACCATATTCTGAATCT-3′	

### Protein extraction and western blot assay

Tumor samples were mechanically homogenized in RIPA buffer (0,1% SDS, 1% NP40, 0.5% CHAPS) supplemented with protease inhibitors aprotinin, sodium orthovanadate and phenylmethylsulphonyl fluoride (Sigma-Aldrich). After 30 min-incubation on ice, whole tumor lysates were centrifuged at 14,000 rpm, 4 °C, for 20 min. The supernatant was collected, quantified via Bradford method (Bio-Rad) and stored in aliquots at − 80 °C to avoid repeated freezing–thawing cycles. For Western Blot analysis an equal amount of protein lysates (20–40 mg depending on the target assayed) were separated onto Criterion TGX precast gels (Bio-Rad) and transferred to a polyvinylidene difluoride (PVDF) membrane (Millipore) using Criterion Blotter (Bio-Rad). Membranes were blocked with 5% BSA-TBS-T and then overnight incubated with primary antibodies at 4 °C. Secondary antibody-binding was performed at RT for 1 h. After TBS-T washing, immunoreactive bands were incubated with enhanced chemiluminescent reagent (Euroclone) and detected via ChemiDoc XRS + System (Bio-Rad). Densitometry analysis was accomplished through ImageJ software. In all the *TBX3* knockdown experiments on human cell lines, the anti-mouse IgM and IgG (H + L) cross adsorbed, DyLight 680 (Thermo Scientific) was used as secondary antibody and blots were analyzed using the Odyssey Scanner and Image Studio Software (LI-COR). All WB experiments were done including n = 2–5 biological replicates per group. Some blots were cut before antibody hybridization to accommodate multiple antibodies on the same blot. The original acquisition images and additional duplicates can be found in Supplementary Information. All the antibodies used were previously validated for WB and are summarized in Table 2.

**Table 2:** Summary of used antibodies.

Primary antibodies				
Antigen	Antibody	Application	Dilution	Brand
Phospho-HER2	Rabbit monoclonal anti-phospho HER2/ErbB2 (Tyr1248)	WB	1:1000	Cell signaling technology
HER2	Rabbit monoclonal anti-her2/erbb2			
	Rabbit polyclonal anti-neu	IHC	1:250	Santa Cruz BT
		FC	1:50	
GAPDH	Mouse monoclonal anti-gapdh	WB	1:500	Santa Cruz BT
Phospho-SIRT6	Rabbit polyclonal anti-phospho sirt6 (ser338)	WB	1:1000	Biorbyt
SIRT6	Rabbit monoclonal anti-sirt6	WB	1:1000	Cell signaling technology
	Rabbit polyclonal anti-sirt6	IHC	1:300	Thermo scientific
		FC	1:40	
Phospho-SRC	Rabbit monoclonal anti-phospho src (tyr416)	WB	1:1000	Cell signaling technology
SRC	Rabbit monoclonal anti-src			
Phospho-STAT3	Mouse monoclonal anti-phospho stat3 (tyr705)			
STAT3	Rabbit monoclonal anti-stat3			
Phospho-MAPK p38	Rabbit monoclonal anti-phospho mapk p38 (thr 180/tyr 182)			
MAPK p38	Rabbit monoclonal anti-p38			
Phospho-ERK 1,2	Rabbit monoclonal anti-phospho erk1/2 p44/42 (thr202/tyr204)			
ERK 1,2	Rabbit monoclonal anti- erk1/2 p44/42			
Phospho-AKT	Rabbit monoclonal anti-phospho akt (ser473)			
AKT	Rabbit monoclonal anti-akt			
PI3K p110 alpha	Rabbit monoclonal anti- pi3k p110 alpha (#4249)			
Phospho-4E-BP1	Rabbit monoclonal anti-p4e-bp1 (#2855)			
Raptor	Rabbit monoclonal anti-raptor (#2280)			
b-actin	Rabbit monoclonal anti-b-actin			
Cyclin E	Mouse monoclonal anti-cyclin e			
Cyclin D1	Rabbit monoclonal anti-cyclin d1	WB	1:5000	Epitomics
PI3K p85 alpha	Rabbit monoclonal anti- pi3k p85 alpha (#1675-1)		1:1000	
CD44	FITC-conjugated rat monoclonal anti-CD44	FC	1:50	Life technologies
CD24	PerCP-Cy 5.5-conjugated rat monoclonal anti-CD24		1:40	
Oct 3,4	PerCP-Cy 5.5-conjugated mouse monoclonal anti-Oct 3,4		1:20	BD-Biosciences
NANOG	Alexa Fluor 488-conjugated mouse monoclonal anti-NANOG		1:20	
NOTCH1	PE-conjugated mouse monoclonal anti-NOTCH1		1:40	
8-oxo-dG	Mouse monoclonal anti-8-oxo-dg	IHC	1:250	Trevigen
H3	Rabbit polyclonal to Histone H3 (06–755)	WB	1:500	Merk millipore
H3K9ac	Rabbit polyclonal to Histone H3 (acetyl K9)—chip grade	ChIP	2 μg/IP	Abcam
		WB	1:1000	
TBX3	Mouse monoclonal igm anti-TBX3 (A-6) (for human)	WB	1:500	Santa cruz BT
	Rabbit polyclonal anti-TBX3 (#42–4800) (for mouse)	IHC, WB	1:500	Thermo scientific

Secondary antibodies				
Antibody	Application	Dilution	Brand	
HRP-conjugated goat anti-mouse IgG (H&L)	WB	1:3000	Calbiochem	
HRP-conjugated goat anti-rabbit IgG (H&L)	WB	1:20,000	Sigma-Aldrich	
Biotin-conjugated goat anti-rabbit IgG (H&L) #BA-1000	IHC	1:400	Vector Laboratories	
Biotin-conjugated goat anti-rabbit IgG (H&L)	IHC	1:200	Bethyl Laboratories	
Biotin-conjugated goat anti-mouse IgG (H&L)				
Alexa Fluor 488-conjugated goat anti-rabbit IgG (H&L)	FC	1:100	Life Technologies	
Alexa Fluor 488-conjugated goat anti-mouse IgG (H&L)				
Alexa Fluor 555-conjugated goat anti-rabbit IgG (H&L)				
Alexa Fluor 555-conjugated rabbit anti-mouse IgG (H&L)	IF	1:400	Life Technologies	
DyLight 680, goat anti-mouse IgM cross-absorbed	WB	1:5000	Thermo Scientific	
DyLight 680, donkey anti-mouse IgG (H&L) cross-absorbed	WB	1:5000	Thermo Scientific	

*WB* western blot, *FC* flow cytometry, *IF* immunofluorescence, *IHC* immunohistochemistry, *ChIP* chromatin immunoprecipitation.

### Cell culture

All cells were maintained at 37 °C in an atmosphere of 5% CO_2_ and regularly checked for mycoplasma infection as previously described^100^.

#### Primary cell cultures

Tumors collected under sterile conditions underwent enzymatic digestion at 37 °C for 1 h in Dulbecco’s Modified Eagle Medium (DMEM, Lonza) supplemented with collagenase-dispase mix (Sigma). Mechanical dissociation of tumor samples was achieved via gentleMACS Dissociator (Miltenyi Biotec) using m_inptumor_1 protocol (Miltenyi Biotec). The obtained single cell suspensions were cultured up to 5 days in DMEM (Lonza) plus 10% FBS (Gibco) and 1% 100 U/mL penicillin-100 mg/mL streptomycin (P/S, Gibco). For each experimental group three primary cell cultures were obtained from an equal number of tumors.

#### Stable cell lines

Mouse CAM6 breast cancer cells^29^ stably transduced with pLenti-GIII-UbC-mSirt6 (NM_181586, cat # LV466465, ABMgood) and with pLenti-III-Ubc-Blank (cat # LV589, ABMgood) were kindly provided by Dr. Galiè. Cells were maintained in DMEM (Lonza) plus 10% FBS (Gibco) and 0.5 mg/mL puromycin. Human BT474 breast cancer cells stably transduced with pHIV-dTomato (cat # 21,374, Addgene) and with pHIV-dTomato-hSIRT6 (generated in Dr. Coppari lab) were kindly provided by Dr. Galiè. Cells were maintained in DMEM (Lonza), 10% FBS (Gibco), 1% 100 U/mL penicillin-100 mg/mL streptomycin (P/S, Gibco) and 1.5 mg/mL sodium bicarbonate. HEK293 were from ATCC (CRL-1573) and were cultured in EMEM (Lonza) plus 10% FBS (Gibco). The BCM-4888 HER2 + ER + breast cancer patient-derived line^72^ was kindly provided by Dr. Zhang and maintained as previously reported for other patient-derived organoids^101^.

### Flow cytometry

Primary cell cultures were detached to obtain single cell suspensions (1X trypsin, Euroclone). 10^6^ cells per sample were used for flow cytometry analysis via BD FACScalibur (BD Biosciences). To detect membrane-associated proteins, cells were washed in staining buffer (0.1% NaN_3_, 0,2% FBS in PBS), incubated with primary antibodies 1 h at 4 °C and then stained (whenever necessary) with the proper fluorescent secondary antibodies, 30 min at 4 °C. To assay intracellular targets, cells were fixed and permeabilized by mean of BD cytofix and BD cytoperm solutions (BD Biosciences) according to manufacturer guidelines. All the antibodies employed are listed in Table 2.

### Cell cycle analysis

5 × 10^5^ primary cells per well were seeded onto 6-well tissue culture plates. The day after, the cells were harvested and fixed with ice-cold 70% ethanol, 1 h at 4 °C. RNA was digested by 1 mg/mL bovine RNase (Sigma) 30 min at 37 °C. Cells were then labeled with 15 mg/mL propidium iodide (PI) 30 min in the dark. Samples were analyzed via BD FACScalibur (BD Biosciences) and data elaborated via FlowJo software (v8.7).

### Cell migration assay

Cell migration assay was performed using transwell PET membrane inserts (6.5-mm diameter, 8-µm pore size, BD Biosciences) in 24-well tissue culture plates. The bottom surfaces of the transwell membranes were coated with fibronectin (10 mg/mL), let to dry and exposed to UV light overnight. After 24 h of starvation, cells were re-suspended in serum-free medium (2% BSA DMEM). 2 × 10^4^ cells/insert were seeded on the upper transwell surface and allowed to migrate through the membrane for 16–20 h. In negative controls, the lower chamber was filled with serum-free DMEM, while for the other samples conditional medium derived from either Delta16HER2 or Delta16HER2/SIRT6-OE primary cultures was used as chemo-attractant (1:1 ratio with serum-free DMEM). After removal of non-migrating cells in the upper membrane by cotton swabs, the migrating cells were fixed and stained using Diff-Quik reagents (Baxter Healthcare). The stained membranes were thereafter washed in water, inverted and air-dried. Cell motility was evaluated under microscope by counting the cells crossing the 8-µm pore size membrane in eight randomly chosen fields. dTOMATO-positive cells were directly visualized and quantified with LionHeart FX (Biotek). *siRNA-*transfected cells mammospheres were visualized and quantified after Calcein AM viable staining (Invitrogen).

### Soft agar assay

Anchorage–independent cell growth was assessed using soft agar assay. The day before the assay 6-well plates were coated with 0.5% agar in complete medium (10% FBS, 1% P/S DMEM) and left to solidify overnight at 4 °C. Wells were then overlaid with 4 × 10^4^ cells/well suspended in 0.35% agar in complete medium. The plates were incubated at 37 °C, 5% CO_2_ up to 15 days. 500 mL of complete medium/well was added once a week. Colonies were stained with 0.05% crystal violet solution overnight at 4 °C and then visualized under stereomicroscope (Zeiss). Captured images were analyzed via ImageJ software in order to quantify both colony number and colony width.

### Mammosphere formation

10^5^ primary cells per well were grown onto Corning ultra-low attachment 6-well microplates with MEBM basal medium (Lonza) supplemented with 1:50 diluted B27 (Invitrogen), 10 ng/mL murine EGF (Sigma), 20 ng/mL murine bFGF (Sigma), 5 mg/mL insulin, 0.4% BSA and 4 mg/mL heparin (Sigma). The obtained spheres were enzymatically digested with 0.05% trypsin and 0.53 mM EDTA (Invitrogen) and sub-cloned in Corning ultra-low attachment 96-well plates by limiting dilute cell suspension (1 cell/well). The same procedure was repeated to obtain the second cloning. Efficiency of mammosphere formation was monitored under OLYMPUS IX71 microscope. For *siRNA-*transfected cells mammospheres, they were visualized and quantified after Calcein AM viable staining (Invitrogen). Captured images were analyzed in terms of colony number and colony area employing ImageJ software.

### Immunohistochemical (IHC) analysis

Tumors and lungs were surgically removed from euthanized mice of both groups, fixed in 4% PFA in PBS for 24–48 h and then paraffin embedded. 6 mm-thick tissue slices were de-paraffinized and Hematoxylin–Eosin (H&E) stained (tumors and lungs) or subjected to IHC analysis to detect HER2 and SIRT6 positivity (only tumors). Briefly, after antigen retrieval in Tris- HCl EDTA, pH 6 (× 2 times microwave for 5 min) and in PBS-TritonX-100 0.3% (PBS-T) for 20 min, slides underwent peroxidase blocking (H_2_O_2_ water solution for 20 min) and unspecific site-blocking in 3% BSA in PBS-T. Primary antibody incubation was carried out overnight at 4 °C while secondary antibody binding was performed at RT for 30 min. After washing in PBS-T, sections were incubated in 1:100 diluted ABC-peroxidase solution for 30 min. Substrate diaminobenzidine (DAB) was used as chromogen and hematoxylin as counterstaining. Used antibodies are available in Table 2.

### Senescence associated ß-galactosidase detection

Senescence associated ß-galactosidase (SA-ß gal) was detected using the senescence detection kit (abcam ab65351) following the manufacturer’s instructions.

### Oxidative DNA damage

Tumor tissue sections obtained as described above, underwent staining for 8-hydroxy-2’deoxyguanosine (8-oxo-dG) to determine the extent of oxidative DNA damage in the two experimental groups. Briefly, sections were deparaffinized, air-dried, and fixed in acetone:methanol (1:1), rehydrated in decreasing concentration of ethanol and finally in PBS. Tissue slices were then sequentially treated with Proteinase K (5 μg/mL) for 30 min, RNase (100 μg/mL) for 15 min, followed by 2 N HCl incubation for 30 min at 37 °C to denature DNA. The sections were washed in PBS and then incubated with 10% BSA solution for 30 min. Incubation with anti-8-oxoG antibody (Trevigen) was carried out at 4 °C overnight. Primary antibody binding was detected using biotin-conjugated goat anti-mouse IgG (H&L) (Bethyl Laboratories) following the aforementioned procedure. Staining intensity was assessed using ImageJ.

### ChIP-seq

#### Chromatin preparation, immunoprecipitation and sequencing

Tumors from mice were surgically removed as described above, flash-frozen in liquid nitrogen and stored at − 80 °C. About 20 mg of tissue were homogenized with a Dounce homogenizer in 0.5 mL of fixation solution (5 mM HEPES pH 7.5, 10 mM NaCl, 0.1 mM Na_2_EDTA, 0.05 mM EGTA, 1% formaldehyde) and fixed for 10 min at 37 °C. Cells were then lysed and subjected to chromatin extraction as previously reported^102^. Briefly, after resuspension in 0.3 mL of extraction buffer (10 mM Tris–HCl pH 7.4, 0.15 M NaCl, 3 mM CaCl_2_, 2 mM MgCl_2_, 0.1% SDS), samples were sonicated 6 times with pulses of 30 and 60 s on/off respectively, in a refrigerated thermoblock, with an amplitude of 40% using the EpiShear sonicator (Active Motif, Carlsbad, CA, USA). After clarification via centrifugation, chromatin supernatants were recovered. 30 μl of the clarified chromatin was purified using the PCR Purification Kit (QIAGEN, Hilden, Germany) and employed for DNA amount estimation using the dsDNA HS Assay Kit (Invitrogen, Eugene, OR, USA) and Qubit (Invitrogen, Eugene, OR, USA). Chromatin size was assessed by agarose gel electrophoresis. Chromatin immunoselection was conducted as previously described^103,104^ using anti-H3K9ac antibody (ab4441, Lot. GR32651091; Abcam, Cambridge, UK). Immunoselected DNA, once purified and quantified, was preliminary used to test the immunoprecipitation specificity by Real-time qPCR and then processed for libraries preparation and, finally, sequenced in 51 bp pair-ends mode on a NovaSeq 6000 sequencer (Illumina Inc., San Diego, CA, USA).

#### ChIP-seq bioinformatic analysis

All fastq files were aligned to the mouse reference genome (assembly GRCm38/mm10) using “bwa” (v0.7.17), a software package for mapping low-divergent sequences against a large reference genome^105^. Using the sampe function of bwa, pair-end files were created, and the output (SAM format) files were converted to binary (BAM) format, sorted and indexed using samtools^106^. Unmapped reads, reads with a mapping quality (MAPQ) value smaller than 1, duplicate reads and those that mapped outside of chr 1–19 and ChrX were removed using Samtools. Moreover, Samtools was also used to additionally remove non-uniquely mapped reads and kept only those marked as properly paired. Resulting uniquely mapped and properly paired reads (stored in a standard BAM format) were used for peak detection, using MACS2 software (v2.2.7.1)^107^. BAM files were merged based on group and indexed using Samtools. Merged bam files were then used to generate a BPM (bins per million) normalized bigwig file (a file format for display of dense, continuous data in a genome browser track) using deepTools bamCoverage (v3.5.1) (ChrX was ignored for normalization). Differential binding analysis was performed by the Diffbind R package (v3.2.6) and stringency in the analysis was obtained by creating a consensus dataset for each condition, including peaks that were in all samples of the considered group. Only different bound (DB) sites with a false discovery rate (FDR) ≤ 0.05 were considered^108^. ChIPseeker R package (v1.28.3) was applied to annotate peak files and DB sites using the curated RefSeq set^109^. Venn diagrams were drawn with the R package VennDiagram^110^.

### RNA-seq

#### RNA extraction, library preparation and sequencing

Tissue samples of about 30 mg were disrupted with a Dounce homogenizer in liquid nitrogen and total RNA was isolated using the RNeasy Mini Kit (QIAGEN, Hilden, Germany) and following manufacturer’s instructions. RNA-seq libraries were prepared using the TruSeq Stranded Total RNA Library Prep Gold kit (Illumina, San Diego, CA, USA, Cat #: 20,020,599). Briefly, 1.5 µg of total RNA was subjected to rRNA depletion and fragmentation. The first-strand cDNA synthesis was performed with random hexamers while the second cDNA strand synthesis was performed by substitution of dTTP with dUTP. The double-stranded cDNA was then end-repaired and adenylated. Barcoded DNA adapters were ligated to both ends of the double-stranded cDNA and then amplified. The libraries quality was checked on an Agilent 2100 Bioanalyzer system (Agilent Technologies, Palo Alto, CA, USA), quantified using Qubit 2.0 fluorometer (Invitrogen, Eugene, OR, USA) and sequenced in 51 bp pair-ends mode on a NovaSeq 6000 sequencer (Illumina Inc., San Diego, CA, USA).

#### RNA-seq bioinformatic analysis

Fastq file quality was assessed using FASTQC^111^ and reads were mapped on mouse reference genome (assembly GRCm38/mm10) using Tophat/Bowtie2^112^. Raw gene expression values were obtained with HTseq^113^ and used to perform differential analysis using edgeR package (TMM normalization was applied)^114^. Differentially expressed genes (DEGs) were identified when the following criteria were met: FDR ≤ 0.05, RPKM > 1, log2Fc ≥ 1 (for upregulated DEGs) and log2Fc ≤ -1 (for downregulated DEGs). Gene list enrichment analysis was performed using EnrichR^115–117^.

### siRNA transfection

Mission siRNA Universal Negative Control (# SIC001) and *TBX3* siRNA (# SASI-Hs01-00110889) were from Millipore Sigma. siRNA transfection was performed using Dharmafect Reagent 4 (Horizon Discovery) following the manufacturer’s protocol. Knockdown efficiency was assessed 48 h post transfection.

### Animal ethical approval

All animal experiments were performed in accordance with ARRIVE guidelines and the directive 2010/63/EU on the protection of animals used for scientific purposes. All procedures were approved by the Ethic Committee on Animal Use of the University of Camerino (protocol number 14/2012).

### Supplementary Information

Supplementary Figures.

## Data Availability

ChIP-seq and RNA-seq data have been deposited with accession number GSE216186 (https://www.ncbi.nlm.nih.gov/geo/query/acc.cgi?acc=GSE216186) in GEO repository. Supplementary data are available in Supplementary Information.
