# Supplementary material for: SIRT6 promotes metastasis and relapse in HER2-positive breast cancer
Source: Sci Rep. 2023 Dec 12;13:22000. doi: 10.1038/s41598-023-49199-7 (PMC10713583; doi:10.1038/s41598-023-49199-7)

Supplementary Information

SIRT6 promotes metastasis and relapse in HER2-positive breast cancer.

Cristina Andreani[1,2,#,*],Caterina Bartolacci[1,2,#], Giuseppe Persico[3], Francesca Casciaro[4], Stefano Amatori[5], Mirco Fanelli[5], Marco Giorgio[3,4], Mirco Galié[6], Daniele Tomassoni[1], Junbiao Wang[1], Xiaoting Zhang[7], Gregory Bick[7], Roberto Coppari[8,9], Cristina Marchini[1,10]* and Augusto Amici[1,10]

[1] Department of Biosciences and Veterinary Medicine, University of Camerino, 62032, Camerino, Italy

[2] present address: Department of Internal Medicine, University of Cincinnati, 45219, Cincinnati, Ohio, USA

[3] Department of Experimental Oncology, IRCCS—European Institute of Oncology, Via Adamello 16, 20139 Milano, Italy

[4] Department of Biomedical Sciences, University of Padua, Via Ugo Bassi 58/B, 35131 Padova, Italy

[5] Molecular Pathology Laboratory "PaoLa", Department of Biomolecular Sciences, University of Urbino Carlo Bo, 61032, Fano, Italy

[6] Department of Neuroscience, Biomedicine and Movement, Section of Anatomy and Histology, University of Verona, 37134, Verona, Italy

[7] Department of Cancer Biology, University of Cincinnati College of Medicine, Ohio, USA

[8] Department of Cell Physiology and Metabolism, University of Geneva, 1211, Geneva, Switzerland

[9] Diabetes Center of the Faculty of Medicine, University of Geneva, 1211, Geneva, Switzerland

[#]These authors contributed equally

[10] co-last authors

* corresponding authors: Cristina.Andreani@unicam.it; Cristina.Marchini@unicam.it

Content

Supplementary Figures (S1-S13)

Supplementary Figure Legends

Uncropped gels and blots

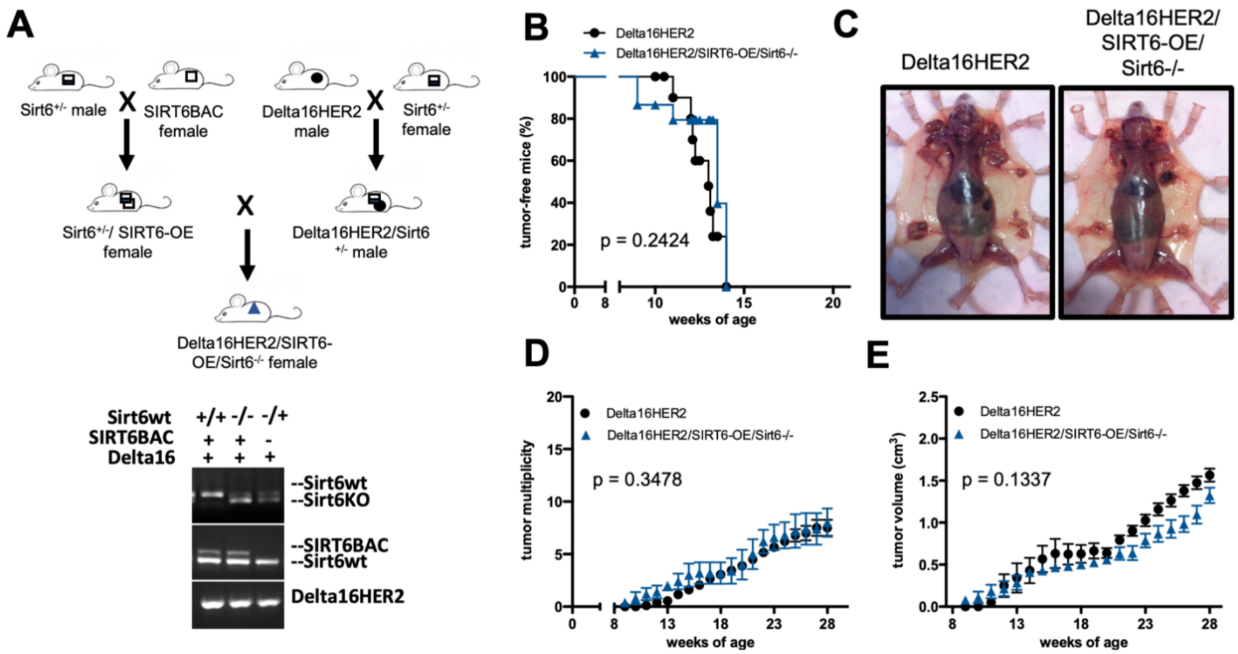

Figure S1. Related to Figure 1. Triple transgenic Delta16HER2/SIRT6-OE/Sirt6$^{-/-}$ mice show no phenotypic differences compared to Delta16HER2 controls. (A) Breeding scheme used to obtain Delta16HER2/SIRT6-OE/Sirt6$^{-/-}$ female mice and a representative genotyping PCR. In these mice SIRT6 is encoded from SIRT6-BAC sequences as they are homozygous for the Sirt6 null allele. **(B)** Kaplan-Meier curves comparing the percentage of tumor-free mice between Delta16HER2 (n=6) and Delta16HER2/SIRT6-OE/Sirt6$^{-/-}$ mice (n=4). Representative *post mortem* pictures of both cohorts are shown in **(C)**.

(D and **E)** Tumor multiplicity and tumor growth curves of Delta16HER2 (n=6) and Delta16HER2/SIRT6-OE mice/Sirt6$^{-/-}$ (n=4).

In **(B)** p=0.2424, not significant (Log-rank test); in **(D** and **E)** p=0.3478 and p=0.1337, not significant (two-way ANOVA followed by Sidak's multiple comparisons test). Error bars represent SD.

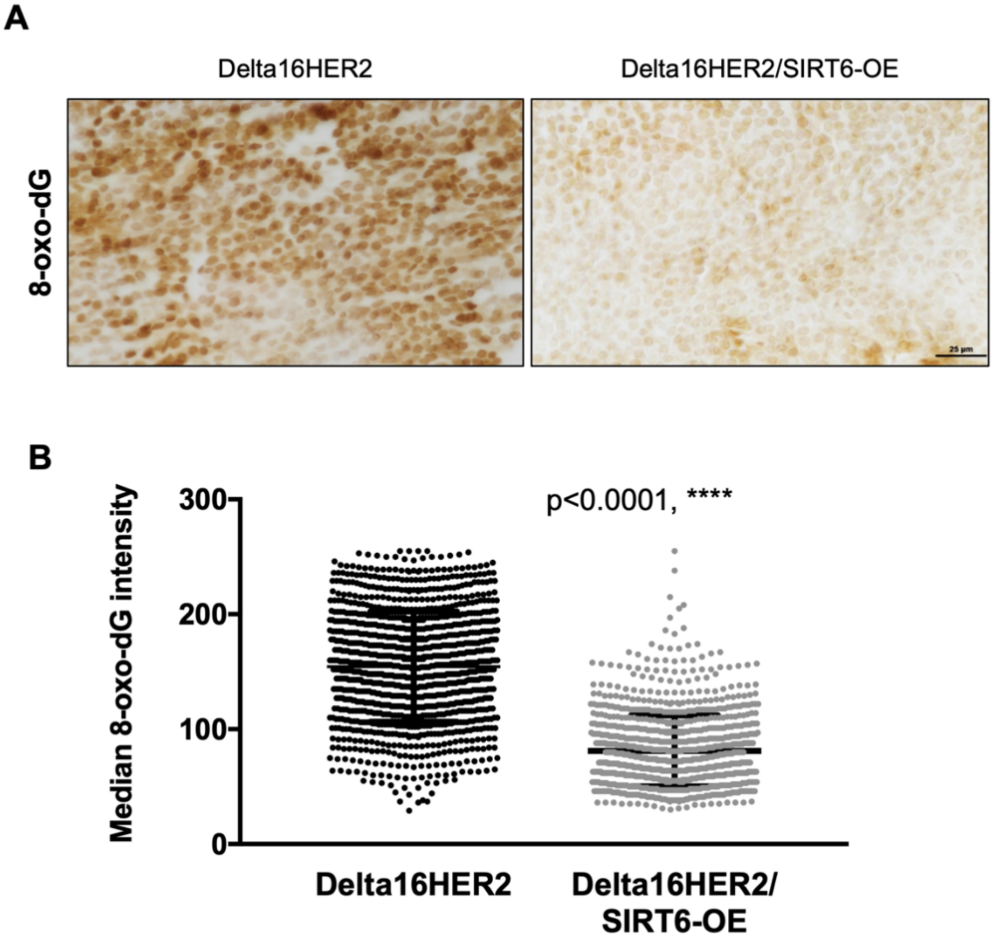

Supplementary Figure S2. Related to Figure 3. SIRT6-OE protects Delta16HER2 cancer cells from oxidative DNA damage. (A) Representative images of IHC staining for 8-oxo-2'-deoxyguanosine (8-oxo-dG) of tumors from 30-week-old Delta16HER2 and Delta16HER2/SIRT6-OE mice. Scale bar, 25 mm.
(**B**) Quantification of median 8-oxo-dG staining using ImageJ. Each spot represents a cell: Delta16HER2 (n=1176) versus Delt16HER2/SIRT6-OE (n=1107), ****p<0.0001 (two-tailed unpaired t test). Error bars represent SD.

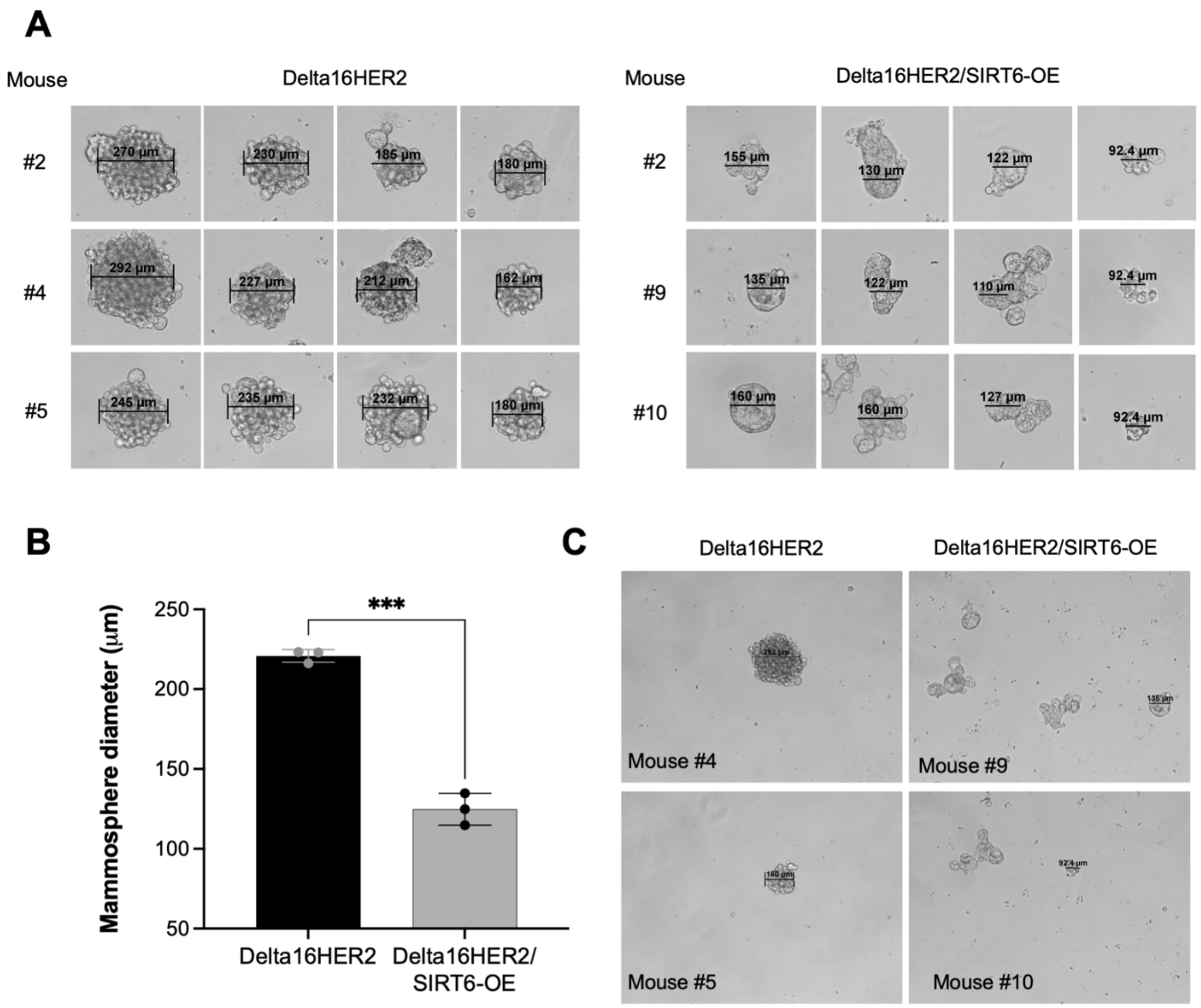

Supplementary Figure S3. Related to Figure 4E. Mammospheres derived from Delta16HER2/SIRT6-OE and Delta16HER2 primary cancer cells morphologically resemble their tumors of origin. (**A**) Mammospheres derived from the indicated tumors and mice (30 weeks of age) and (**B**) quantification of their size. ***p=0.0001 (two-tailed unpaired t test). Error bars represent SD. (**C**) Additional representative full field pictures of mammospheres derived from the indicated tumors and mice (30 weeks of age).

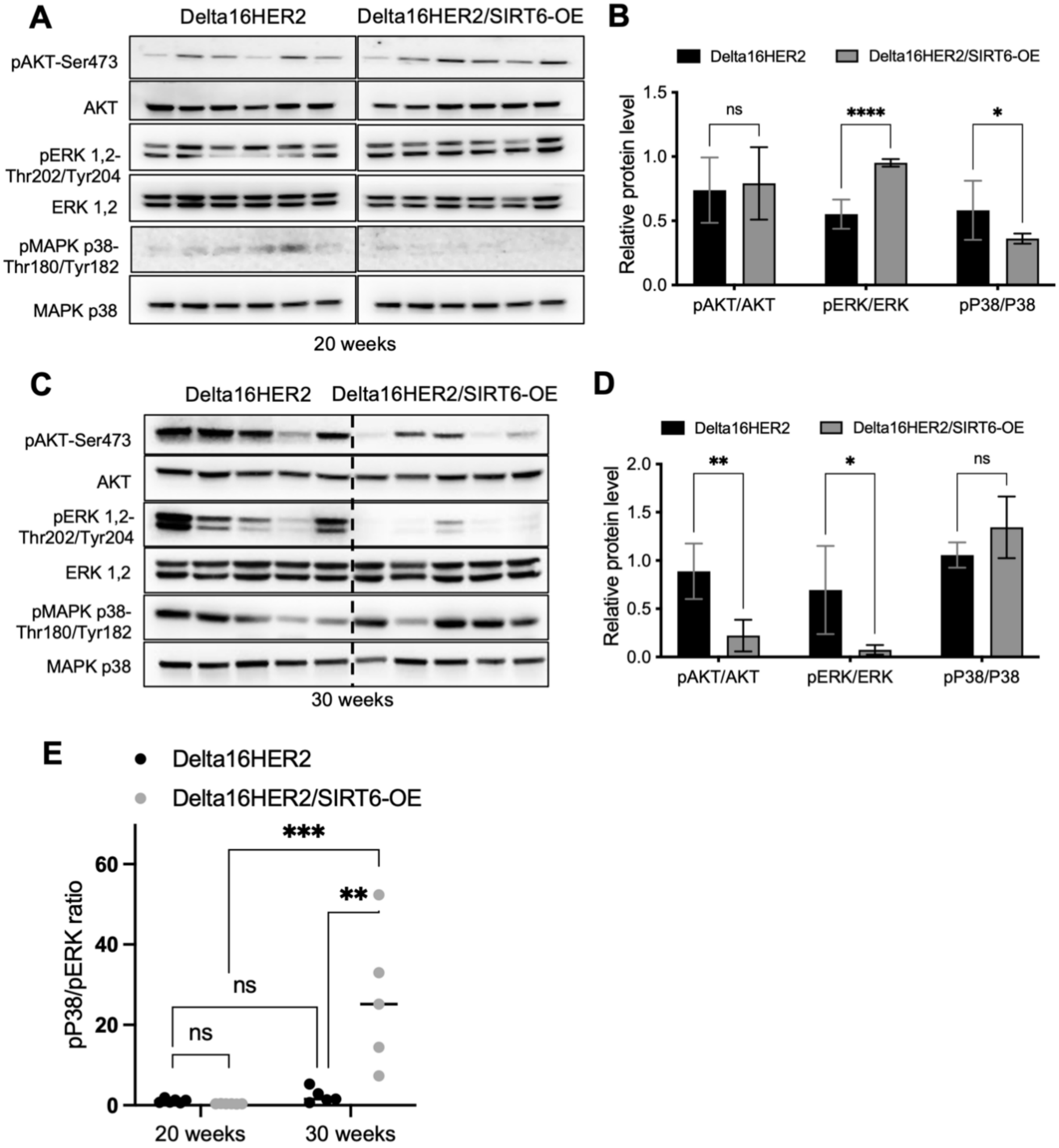

Supplementary Figure S4. SIRT6 molds AKT, ERK1/2 and MAPK-p38 pathways redirecting Delta16HER2 cancer cells toward tumor dormancy. Western blot analysis and relative quantification of AKT, ERK1/2 and MAPK-p38 pathways in tumor protein lysates of Delta16HER2 and Delta16HER2/SIRT6-OE mice at 20 **(A** and **B)** and 30 weeks of age **(C** and **D)**. Phosphorylated proteins are normalized over total protein levels. (**E**) pP38/pERK ratio in the indicated tumor lysates (same samples analyzed in **A-D**).
In **(B** and **D)** p>0.05, not significant (ns); *p<0.05; **p<0.01; ****p<0.0001 (two-tailed unpaired t test). In (**E**) p>0.05, not significant (ns); **p=0.0014; ***p=0.0004 (2-way ANOVA and Sidak's multiple comparisons). Error bars represent SD.

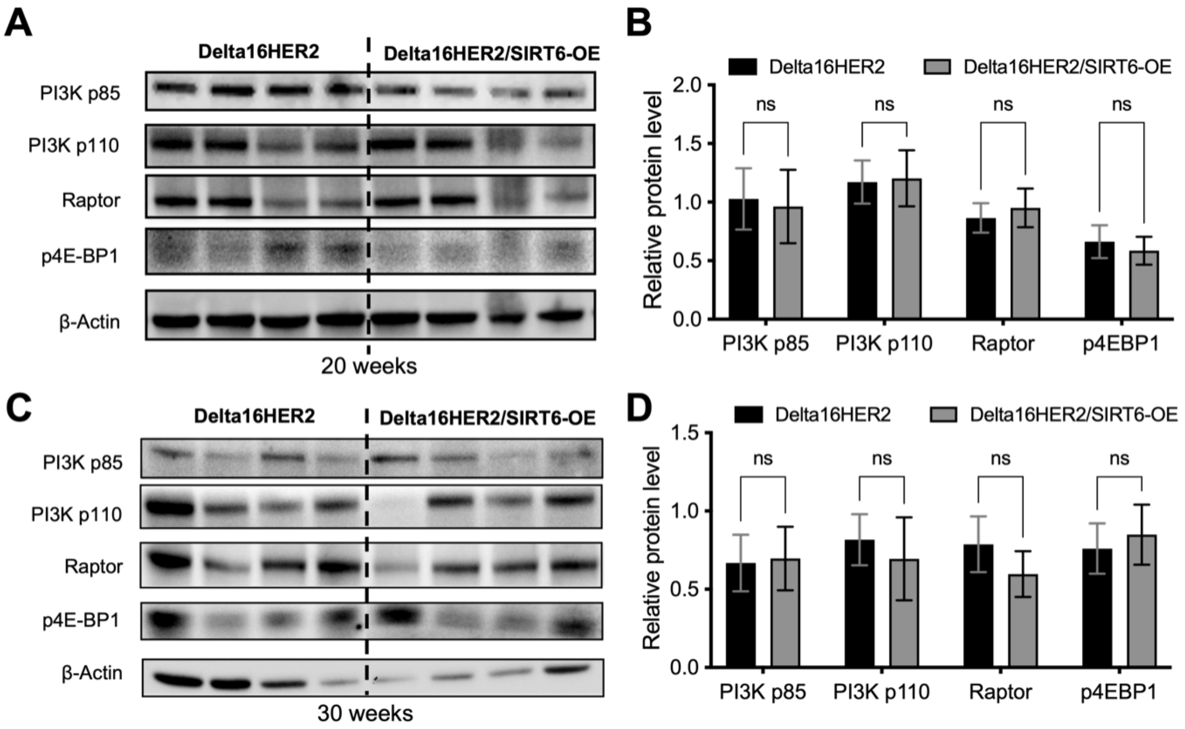

Supplementary Figure S5. SIRT6-OE does not induce changes in the PI3K-mTOR pathway. Western blot analysis and relative quantification of PI3K p85, PI3K p110, Raptor and p4E-BP1 levels in tumor protein lysates of Delta16HER2 and Delta16HER2/SIRT6-OE mice at 20 (**A** and **B**) and 30 weeks of age (**C** and **D**). Protein levels are normalized to ß-Actin.

In **(B** and **D**) $p>0.05$, not significant (two-tailed unpaired t test). Error bars represent SD.

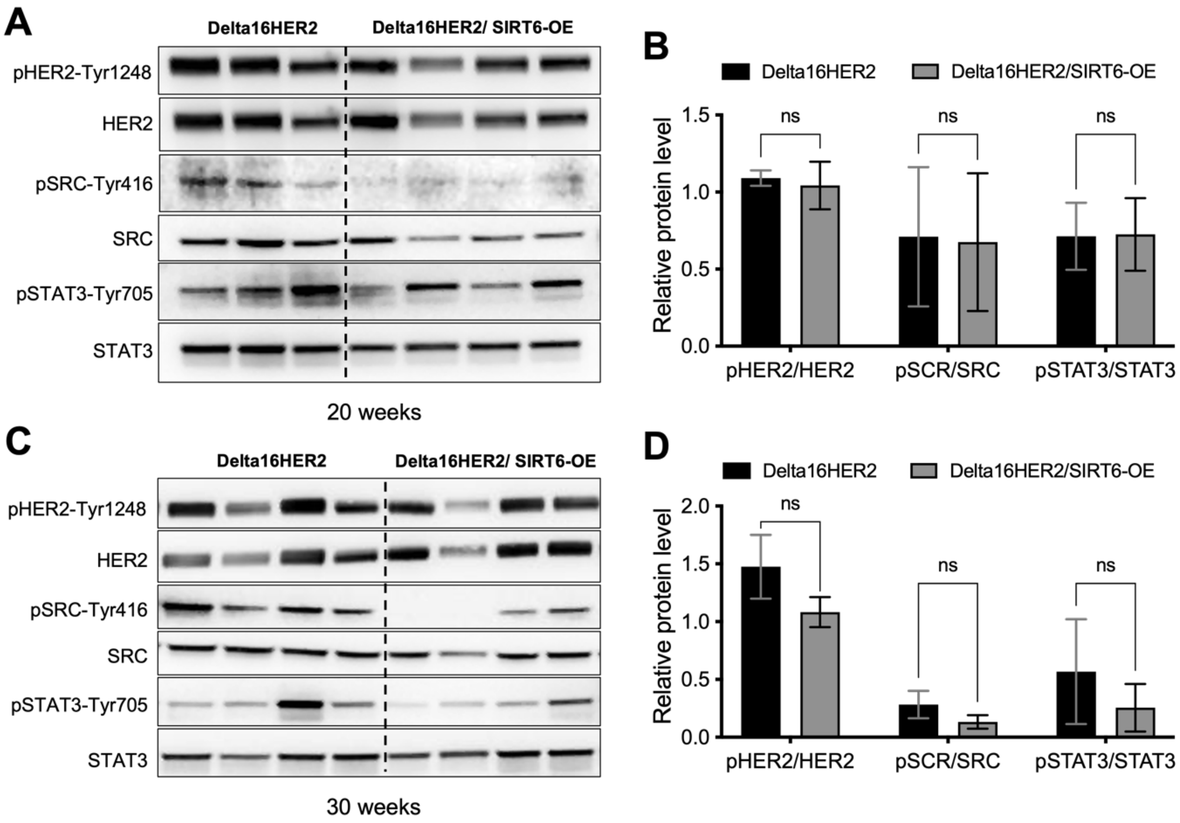

Supplementary Figure S6. SIRT6-OE does not induce changes in the HER2-SRC-STAT3 pathway. Western blot analysis and relative quantification of HER2, SRC and STAT3 activation in tumor protein lysates of Delta16HER2 and Delta16HER2/SIRT6-OE mice at 20 **(A** and **B)** and 30 weeks of age **(C** and **D)**. Phosphorylated proteins are normalized over total protein levels.

In **(B** and **D**) $p>0.05$, not significant (two-tailed unpaired t test). Error bars represent SD of 4 independent experiments.

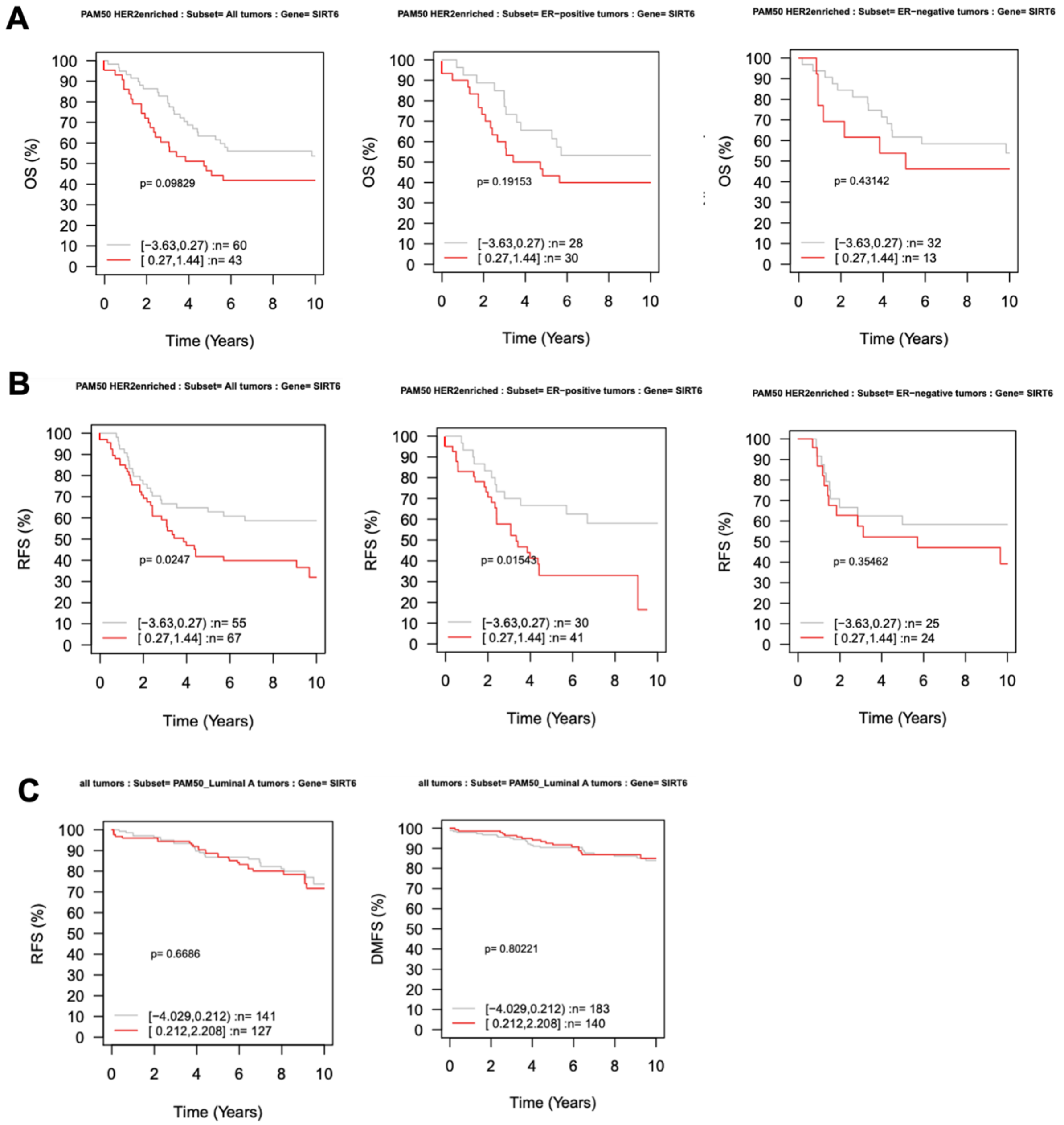

Supplementary Figure S7. Related to Figure 5. SIRT6 effect does not depend on ER and PR status. **(A** and **B)** Kaplan-Meier plots from GOBO database using overall survival (OS, **A**) relapse-free survival (RFS, **B**) in the indicated subsets of HER2-enriched tumors. (**C**) Kaplan-Meier plots from GOBO database using RFS and distant metastasis free survival (DMFS) as outcome in luminal A breast cancer patients. Data were stratified into the two quantiles based on SIRT6 gene expression level (SIRT6_low, grey line and SIRT6_high, red line) using 10-year censoring as endpoint.

P values indicate Log-rank test.

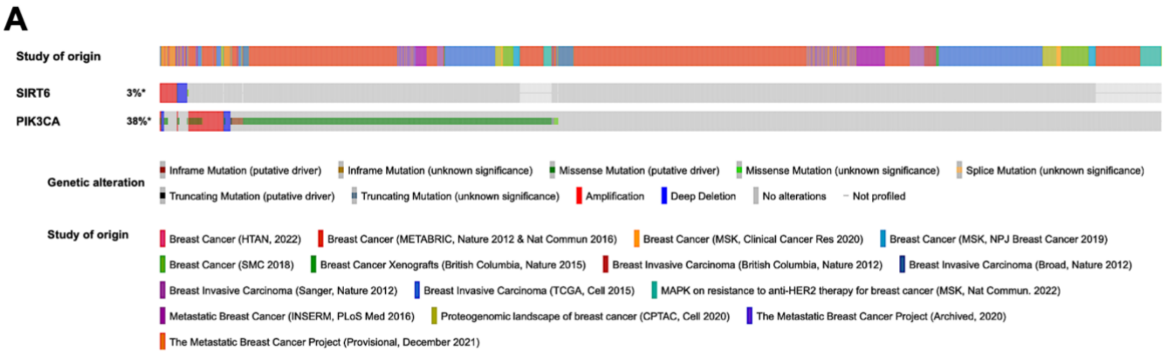

B

Deep del SIRT6 vs Amp/ mut PIK3CA	Value
Neitheir	3654
PIK3CA not SIRT6	1531
SIRT6 not PIK3CA	38
Both	8
Odd Ratio	725.5
Log2 Odd Ratio	9.5
P value	0.046
Tendency	co-occurrence

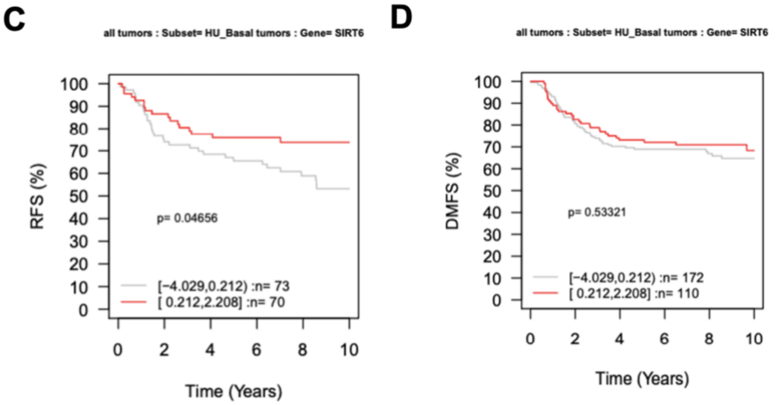

Supplementary Figure S8. Related to Figure 5. SIRT6 correlates with good prognosis in basal-like breast cancer

(A) Snapshot from cBioPortal summarizing the type and frequency of alteration of *PIK3CA* and *SIRT6* genes in the indicated publicly available breast cancer studies. A total of 4860 and 4379 breast cancer patients were profiled for *PIK3CA* and *SIRT6* alterations, respectively. **(B)** Co-occurrence/Mutual exclusivity analysis from cBioPortal indicating the number of patient samples that have amplification/activating mutation of *PIK3CA* and deletion of *SIRT6* alone, both alterations or neither of them. Odd ratio = neither*both / SIRT6 not ERBB2*ERBB2 not SIRT6. Log2 Odd Ratio > 1 indicates co-occurrence of SIRT6 deletion and *PIK3CA* amplifications/mutation (p=0.046, Pearson correlation). **(C** and **D)** Kaplan-Meier plots from GOBO database using relapse-free survival (RFS) (**C**) and distant metastasis free survival (DMFS) (**D**) as outcome in basal-like breast cancer in basal-like breast cancer. Data have been stratified into the two quantiles based on SIRT6 gene expression level (SIRT6_low, grey line and SIRT6_high, red line) using 10-year censoring as endpoint.

In **(C** and **D)** *p=0.04656 and p=0.53321 (Log-rank test).

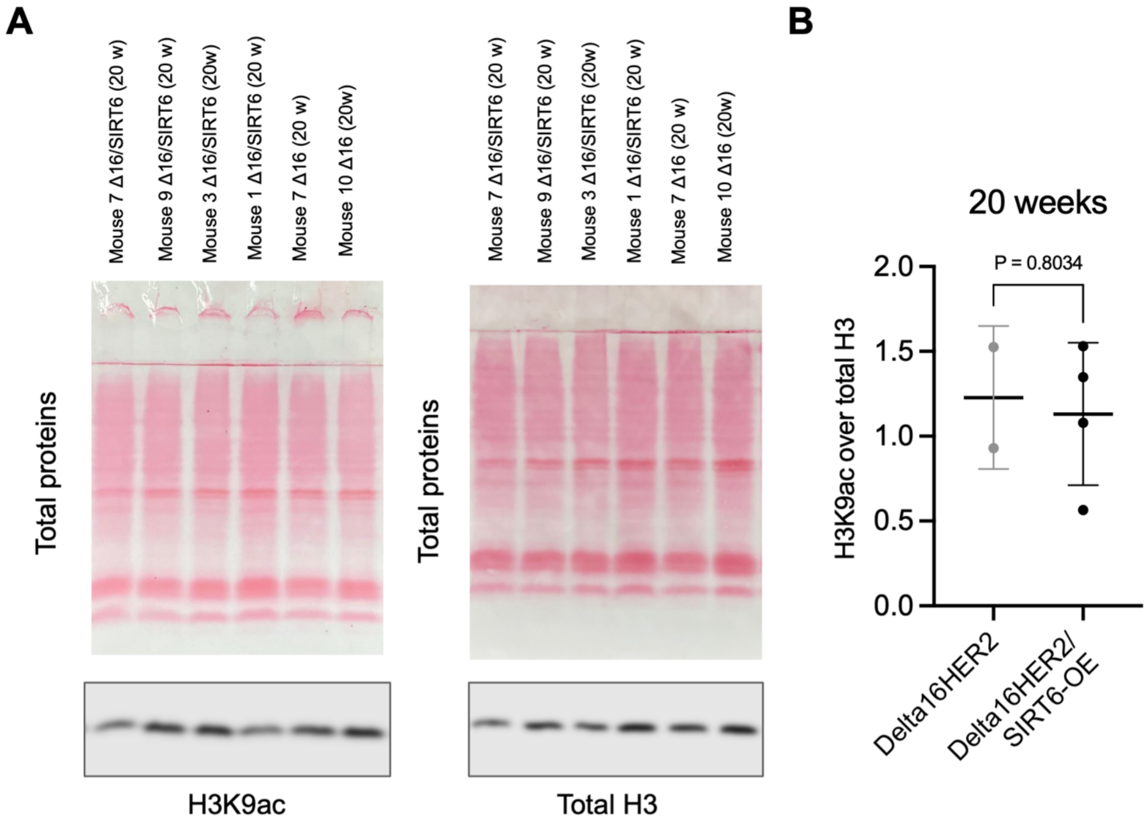

Supplementary Figure S9. Related to Figure 6. SIRT6-OE does not affect total levels of H3K9ac. (**A**) Ponceau red staining and immunoblot for H3K9ac and total H3 in the indicated tumor samples (20 weeks) and their quantification (**B**). ns p=0.8034 two-tailed unpaired t test. Data are reported as mean ± SD.

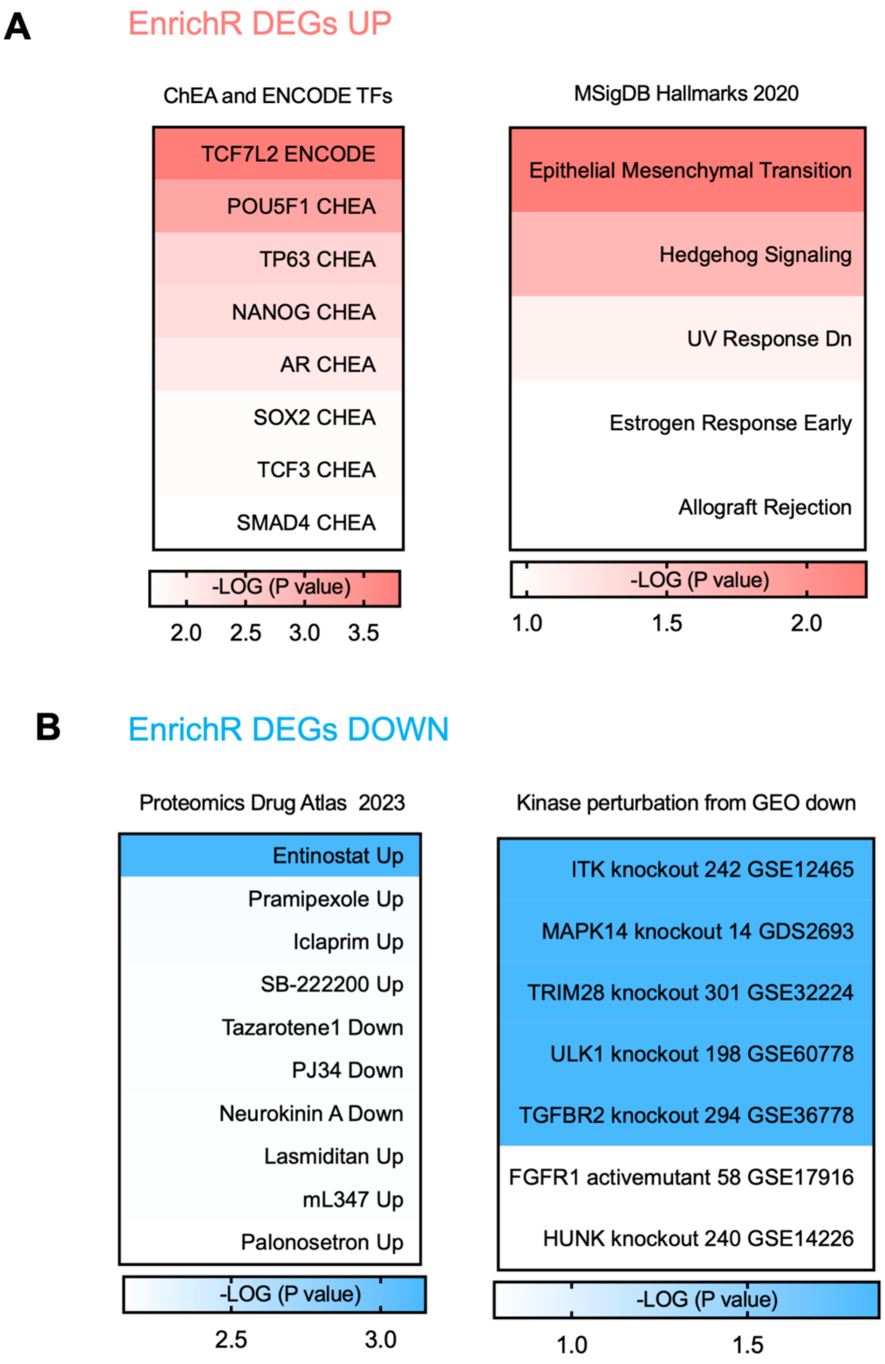

Supplementary Figure S10. Related to Figure 6A-C. EnrichR analysis for genes upregulated and downregulated in Delta16HER2/SIRT6-OE tumors.

Heatmaps showing the pathways and hallmarks for the differentially expressed genes that are upregulated (**A)** and downregulated (**B**) upon SIRT6-OE in mouse tumors (20 weeks).

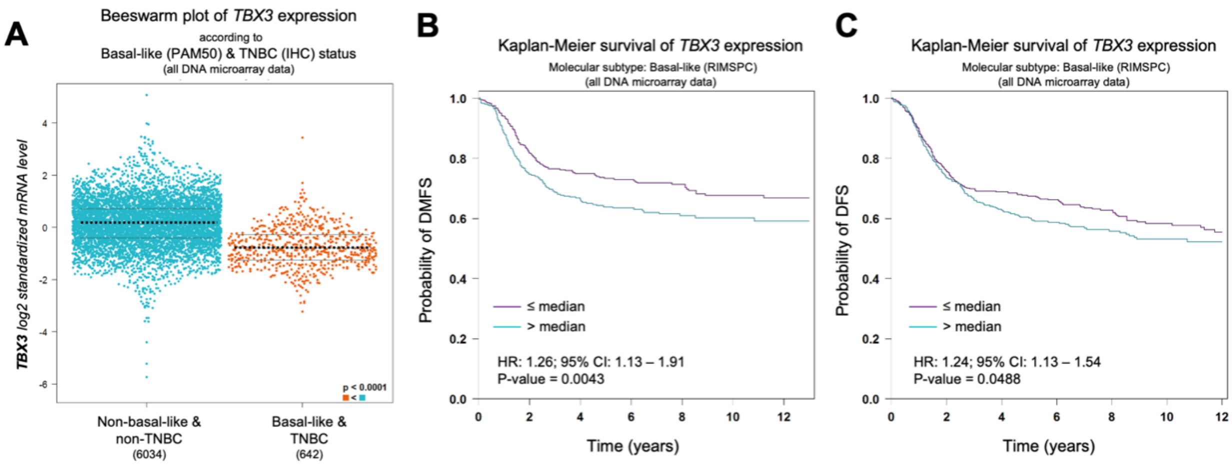

Supplementary Figure S11. Related to Figure 6. High *TBX3* predicts poor prognosis in basal-like tumors.

(**A**) Bee swarm plot computed in bc-GenExMiner showing the expression levels of *TBX3* in basal-like and Triple negative breast cancer (TNBC, n=642) versus non-basal-like/non-TNBC (n=6034). (**B** and **C**) Kaplan-Meier plots from bc-GenExMiner using distant metastasis free survival (DMFS) and disease-free survival (DFS) as outcomes in basal-like breast cancer. Data have been stratified into the two quantiles based on *TBX3* gene expression level (TBX3_low, purple line and TBX3_high, blue line).

In (**B** and **C**) **p=0.0043 and *p=0.0488 (Log-rank test). HR (hazard ratio) and 95% CI (confidence interval) are reported in the figure.

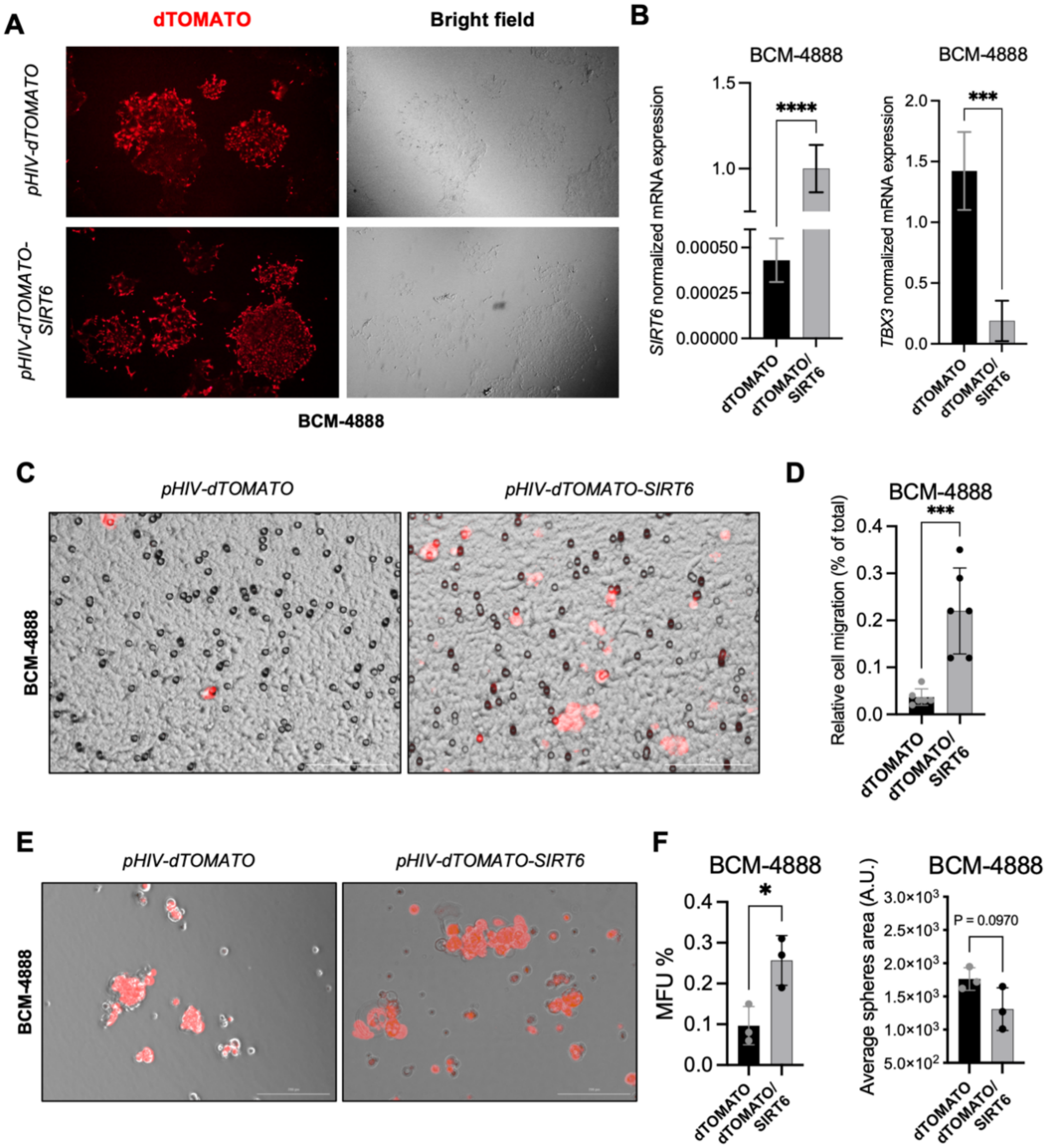

Supplementary Figure S12. Related to Figure 7. SIRT6-OE induces loss of TBX3 and aggressiveness in a patient-derived models of HER2+/ER+ breast cancer (BCM-4888). (**A**) Representative pictures of the patient-derived cell line BCM-4888 transfected with either pHIV-dTomato or pHIV-SIRT6-dTomato. (**B**) Real-time PCR for *SIRT6* and *TBX3* expression in sorted BCM-4888 transfected as indicated. Not transfected HEK293 were used as control sample and *TBP* as housekeeping gene. (**C**) Representative pictures and (**D**) quantification of the Transwell migration assay of BCM-4888 cells transfected with either pHIV-dTomato or pHIV-SIRT6-dTomato. (**E**) Representative pictures and (**F**) number and area quantification of pHIV-dTomato and pHIV-SIRT6-dTomato BCM-4888 mammospheres. MFU%= % of mammary forming units over total number of seeded cells. A.U.= arbitrary unit. Bars indicate mean ± SD. In (**B**) ; (**D**) ***p=0.0007; (**F**) *p=0.0230 and ns, p=0.0970 indicates unpaired two-tailed t test.

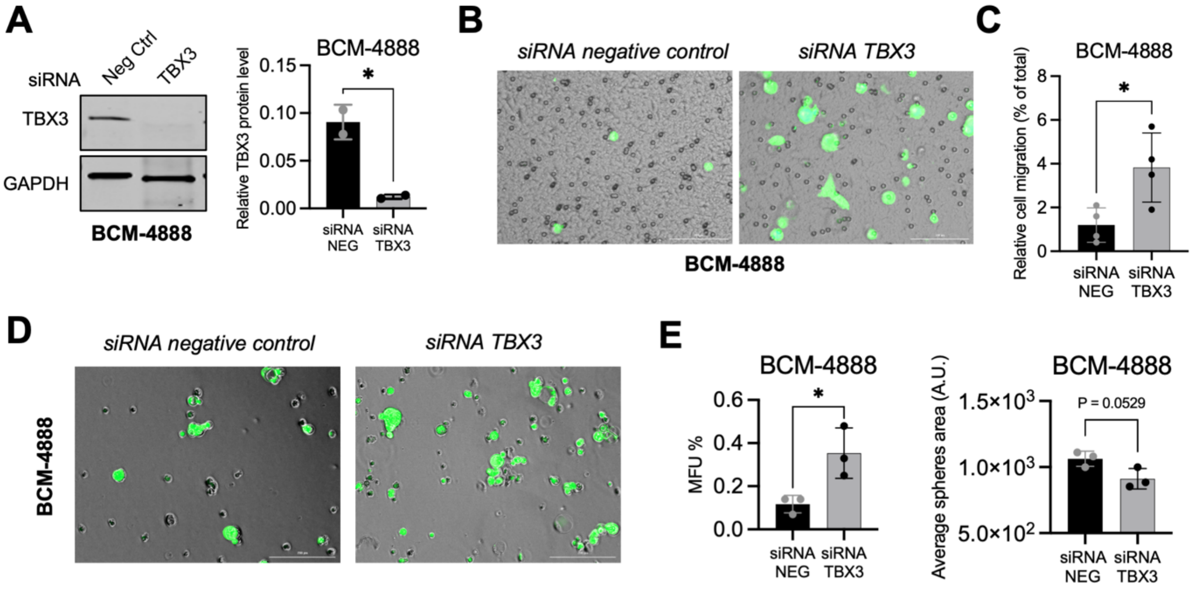

Supplementary Figure S13. Related to Figure 8. Loss of *TBX3* mimics *SIRT6*-OE in a patient derived model of HER2+/ER+ breast cancer (BCM-4888). (**A**) Immunoblot and quantification of TBX3 and GAPDH in lysates of BCM-4888 cells transfected with the indicated siRNA (48 hours post transfection, n=2 independent replicates). (**B**) Representative pictures and (**C**) quantification of the Transwell migration assay of the human BCM-4888 cells transfected as indicated (n=5). (**D**) Representative pictures and (**E**) number and area quantification of siRNA control and siRNA *TBX3* BCM-4888 mammospheres (n=3). MFU%= % of mammary forming units over total number of seeded cells. A.U.= arbitrary unit. Bars indicate mean ± SD. In (**C**) *p=0.0282, (**E**) **p=0.0051 and ns, p=0.2106 indicates unpaired two-tailed t test.
In (**A**) *p=0.0260, (**C**) *p=0.0247, (**E**) *p=0.0294 and ns, p=0.0529 indicates unpaired two-tailed t test.

Original images for gels and blots

Figure 1A
(genomic DNA PCR)

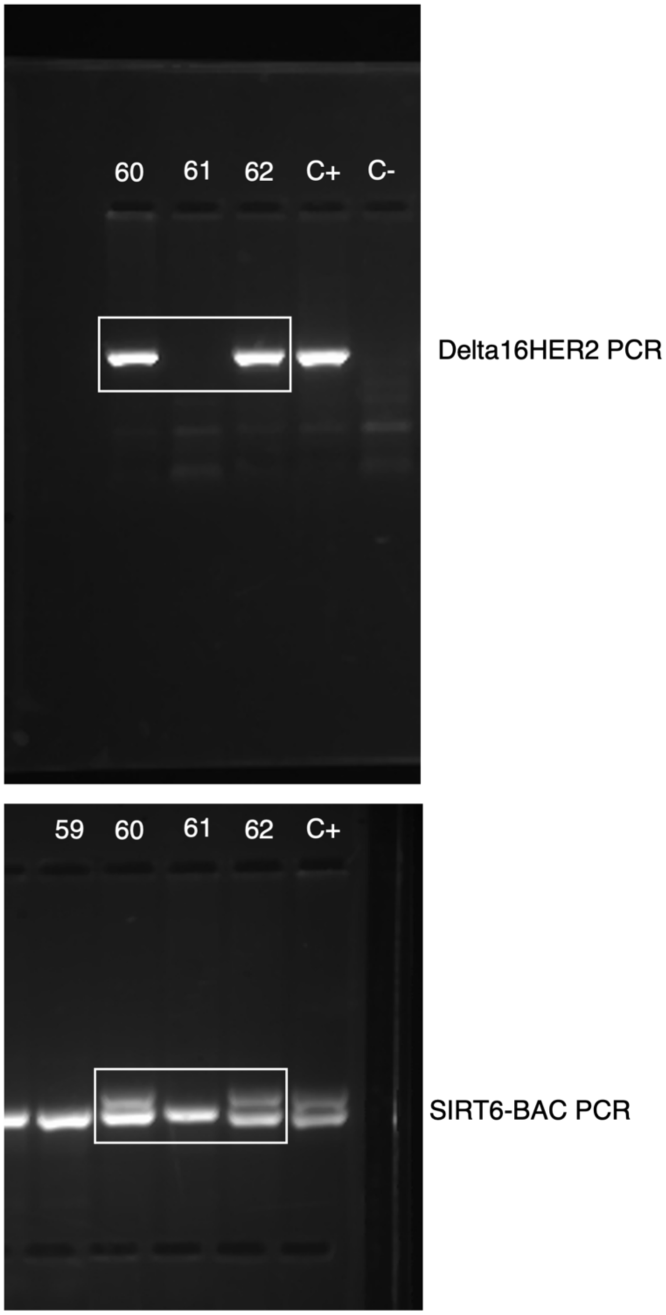

Figure 1H

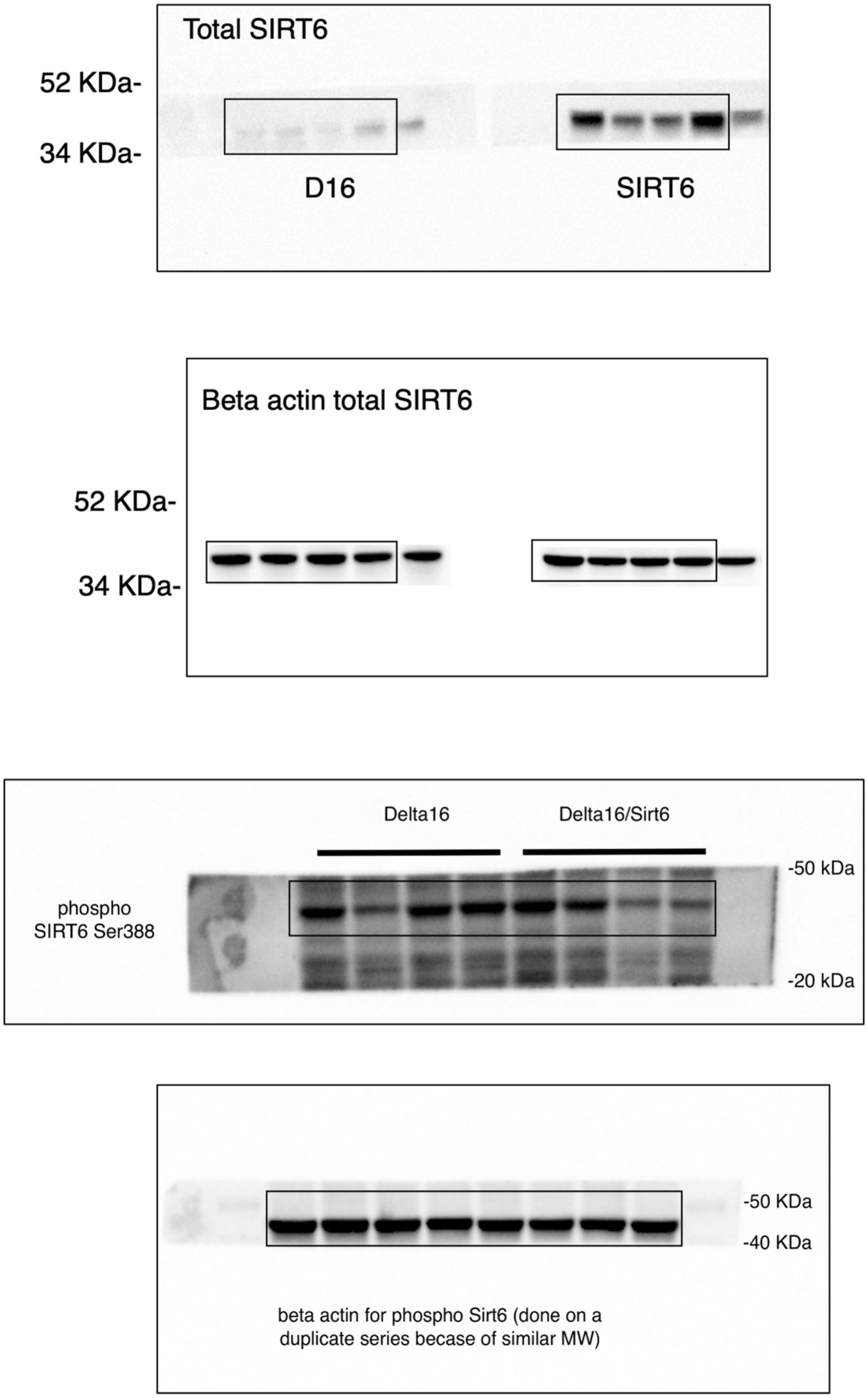
Total SIRT6
52 KDa-
34 KDa-
D16
SIRT6
Beta actin total SIRT6
52 KDa-
34 KDa-
Delta16
Delta16/Sirt6
-50 kDa
phospho
SIRT6 Ser388
-20 kDa
-50 KDa
-40 KDa
beta actin for phospho Sirt6 (done on a
duplicate series becase of similar MW)

Figure 1H
(short exposure/
Low contrast)

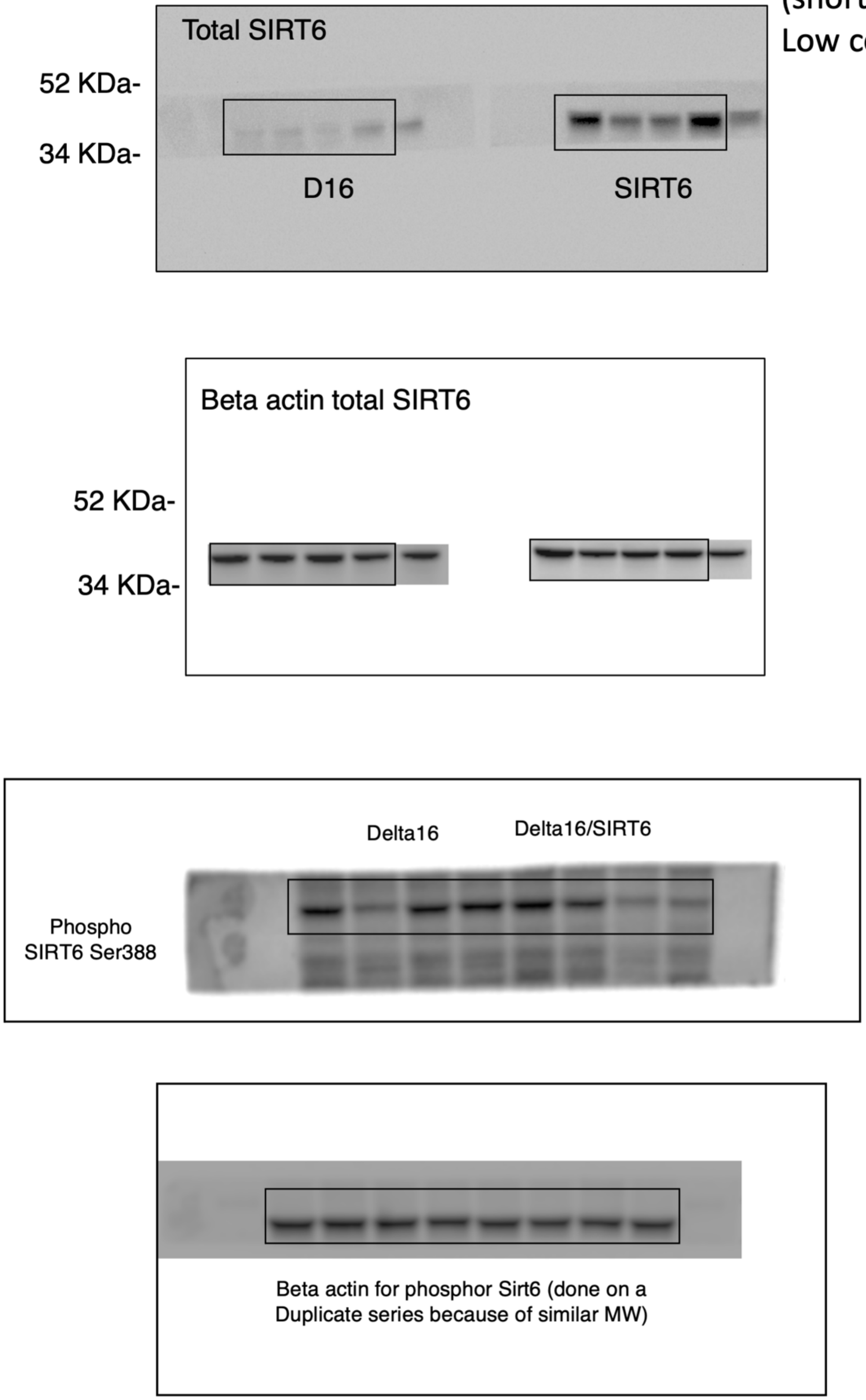

Additional Replicates for Figure 1H

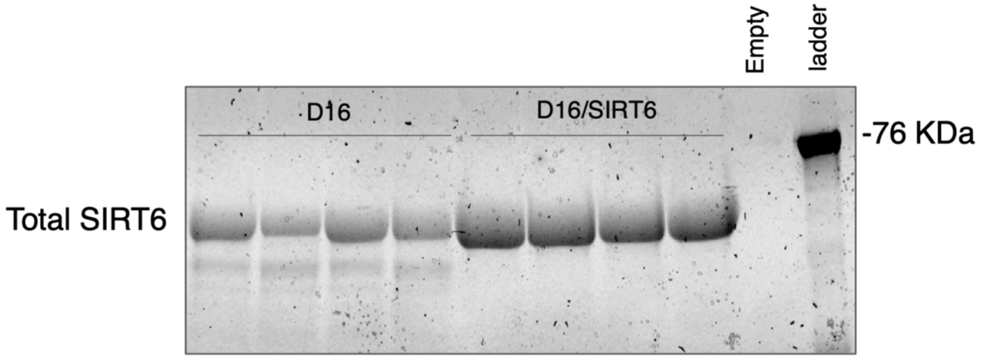

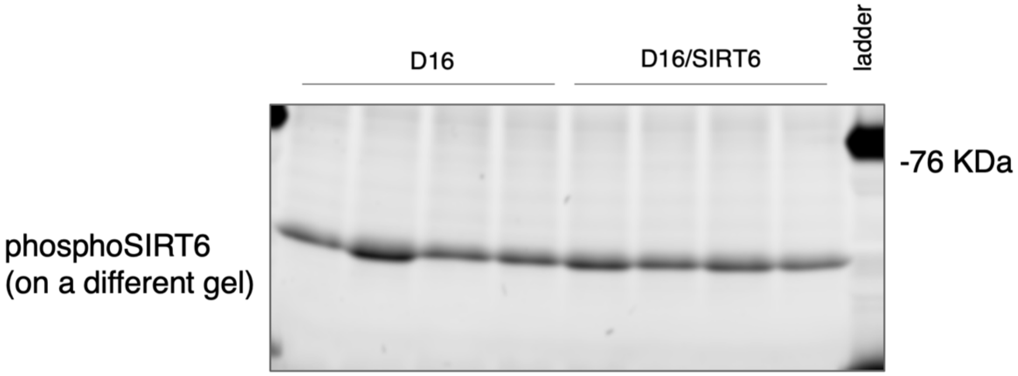

Figure 3B
(short exposure/
Low contrast)

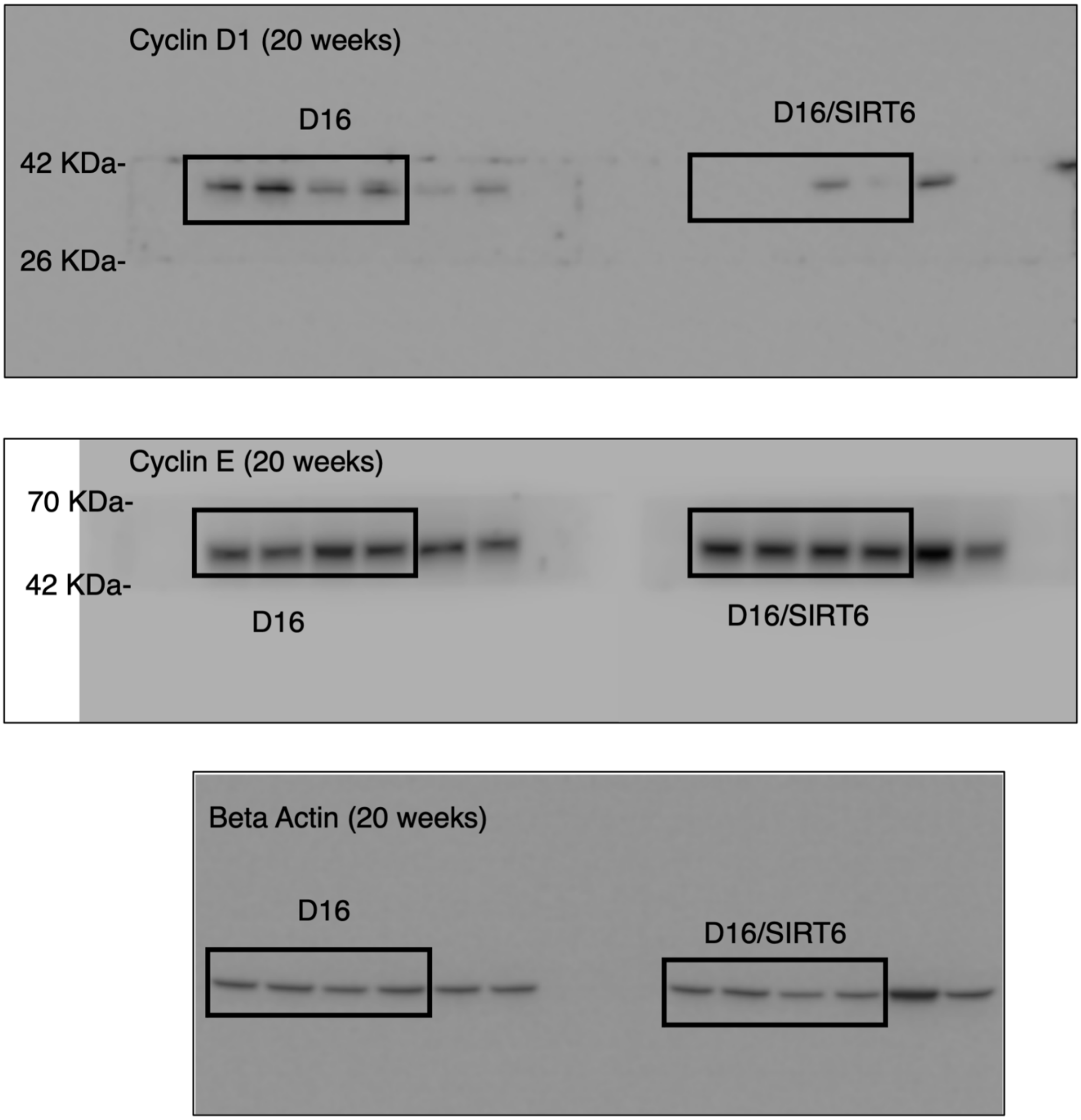

Figure 3B

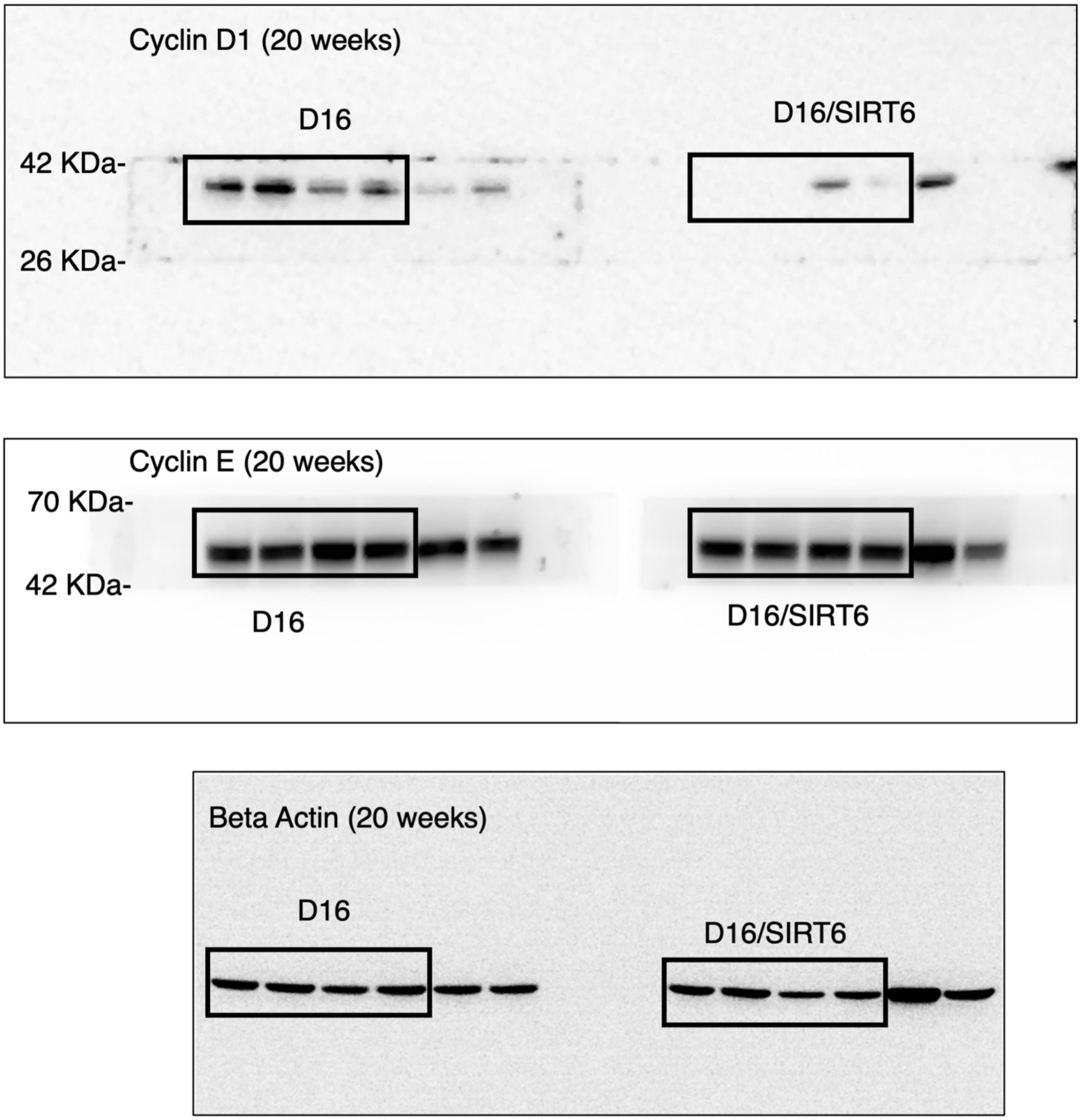
Cyclin D1 (20 weeks)
D16
D16/SIRT6
42 KDa-
26 KDa-
Cyclin E (20 weeks)
70 KDa-
42 KDa-
D16
D16/SIRT6
Beta Actin (20 weeks)
D16
D16/SIRT6

Figure 3B

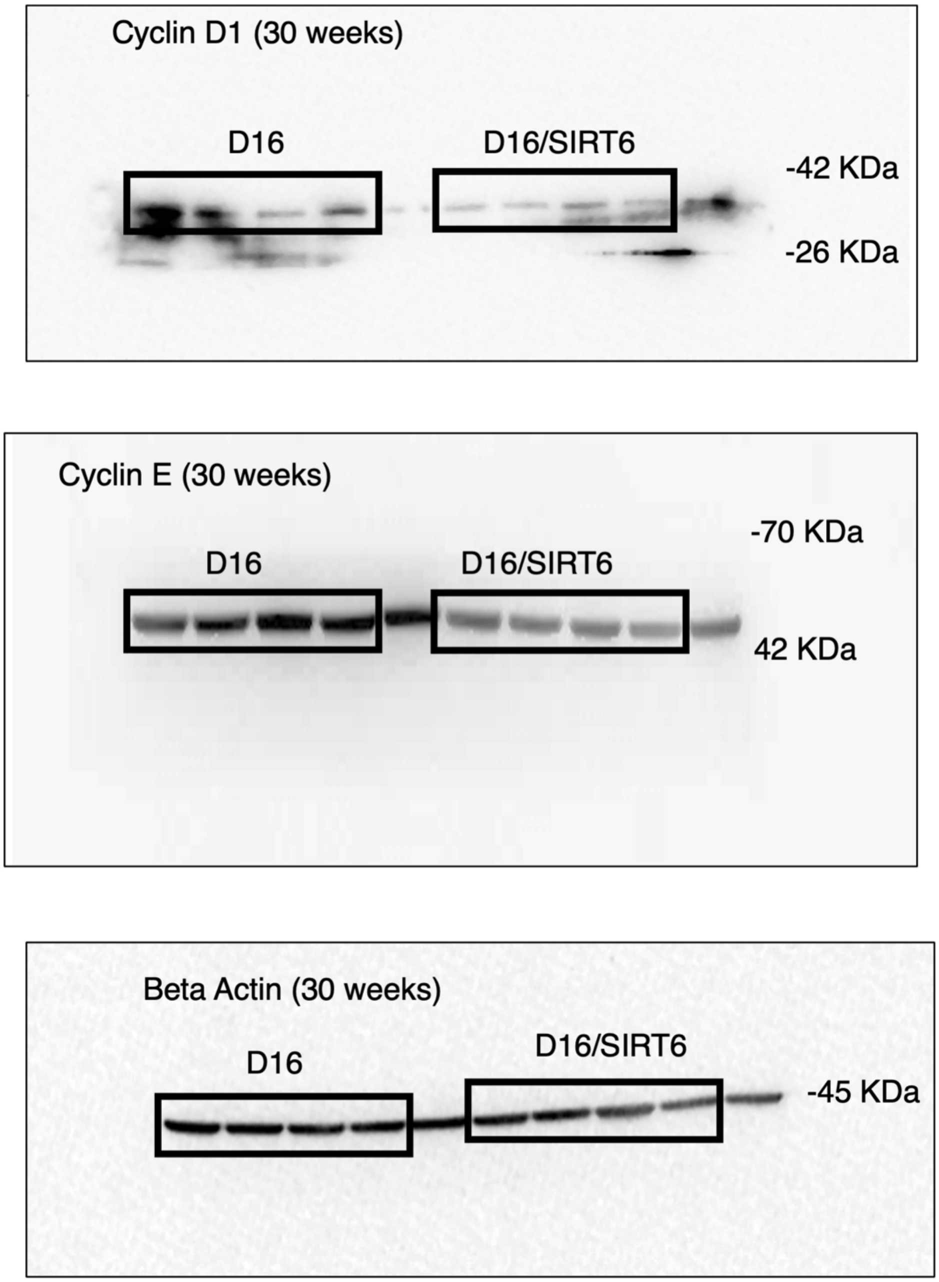

Cyclin D1 (30 weeks)
D16
D16/SIRT6
-42 KDa
-26 KDa
Cyclin E (30 weeks)
-70 KDa
D16
D16/SIRT6
42 KDa
Beta Actin (30 weeks)
D16
D16/SIRT6
-45 KDa

Figure 3B
(short exposure/
Low contrast)

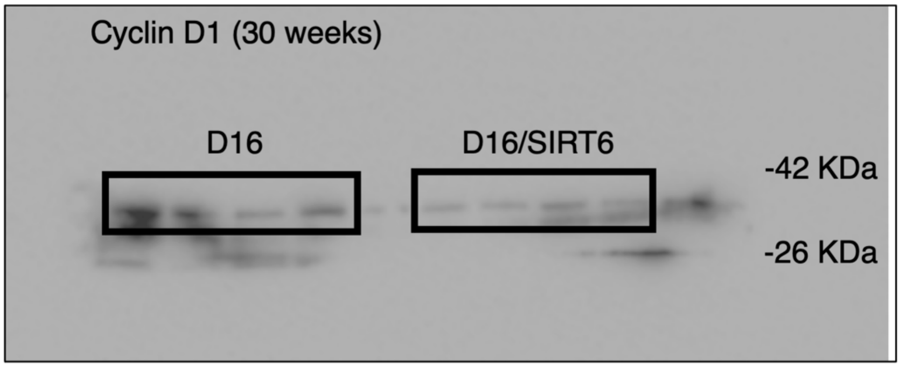

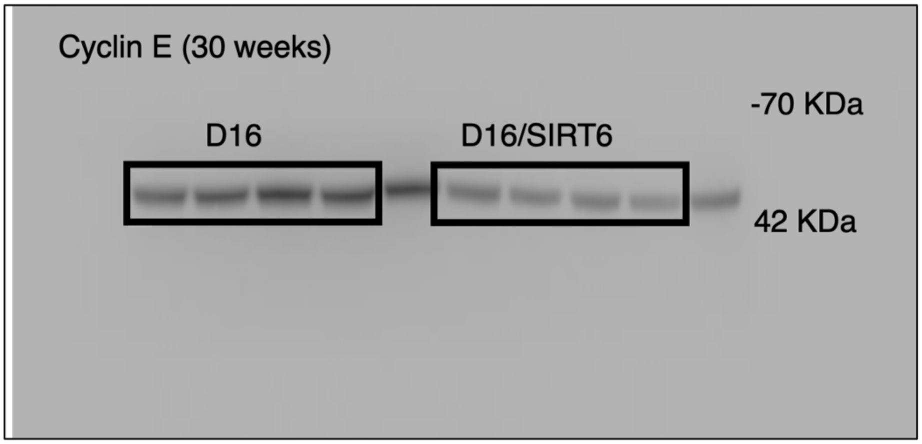

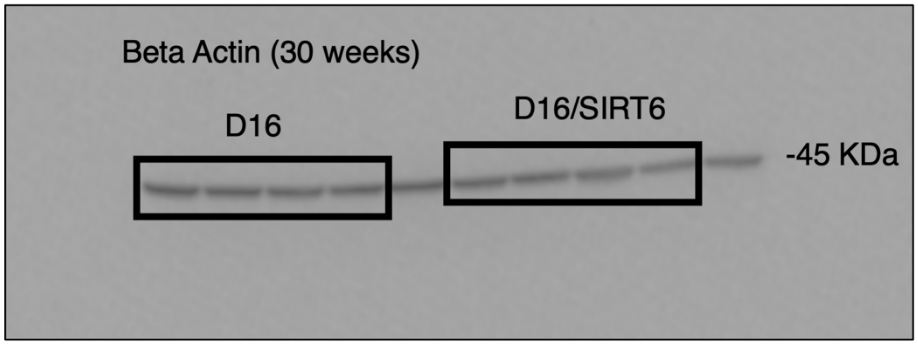

Additional Replicates for Figure 3B

Long exposure

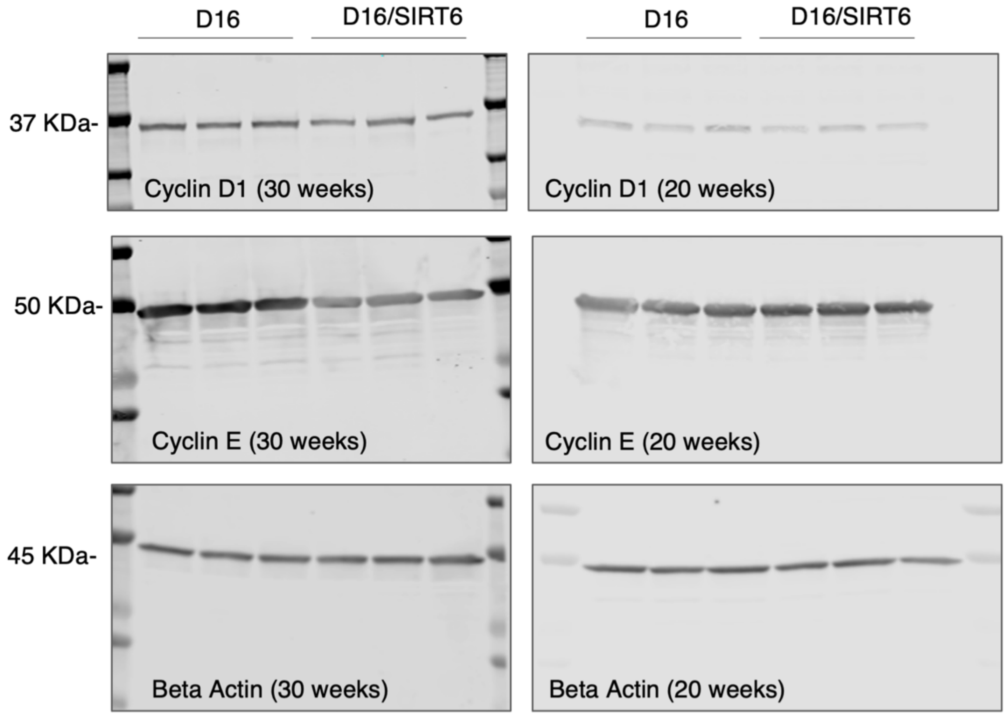

Short exposure

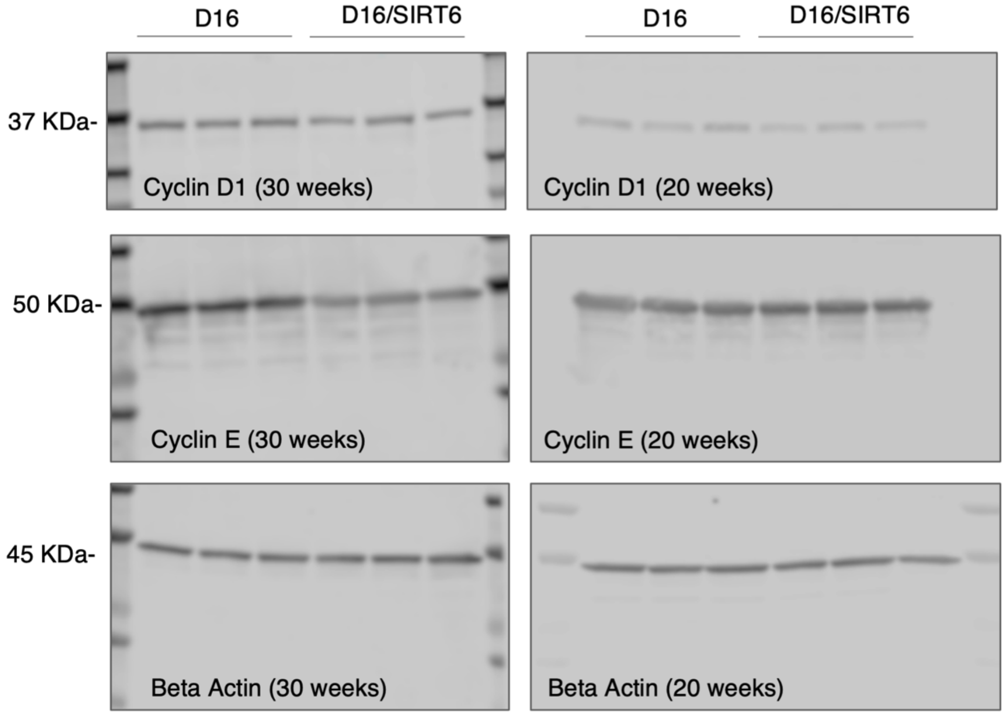

Figure 7C

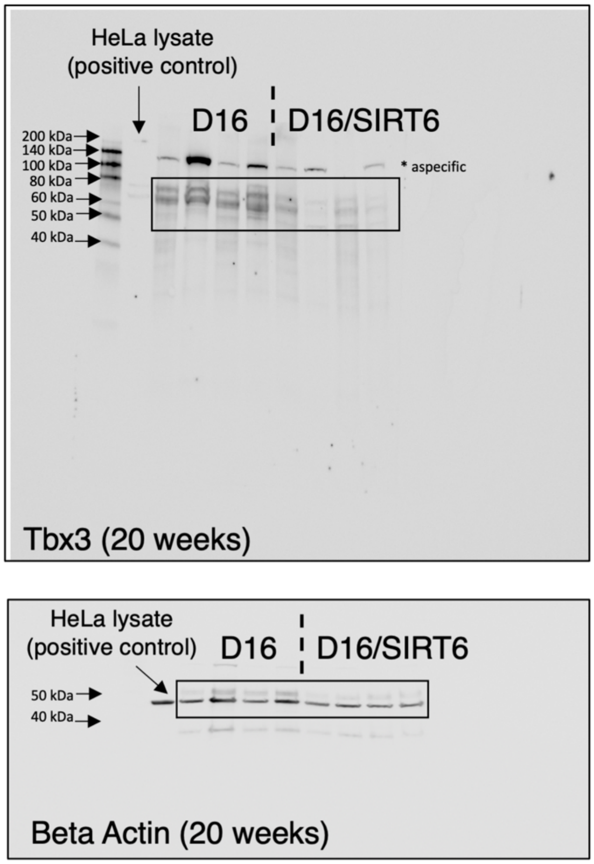
HeLa lysate
(positive control)
D16
D16/SIRT6
200 kDa
140 kDa
100 kDa
80 kDa
60 kDa
50 kDa
40 kDa
* aspecific
Tbx3 (20 weeks)
HeLa lysate
(positive control)
D16
D16/SIRT6
50 kDa
40 kDa
Beta Actin (20 weeks)

Figure 8A

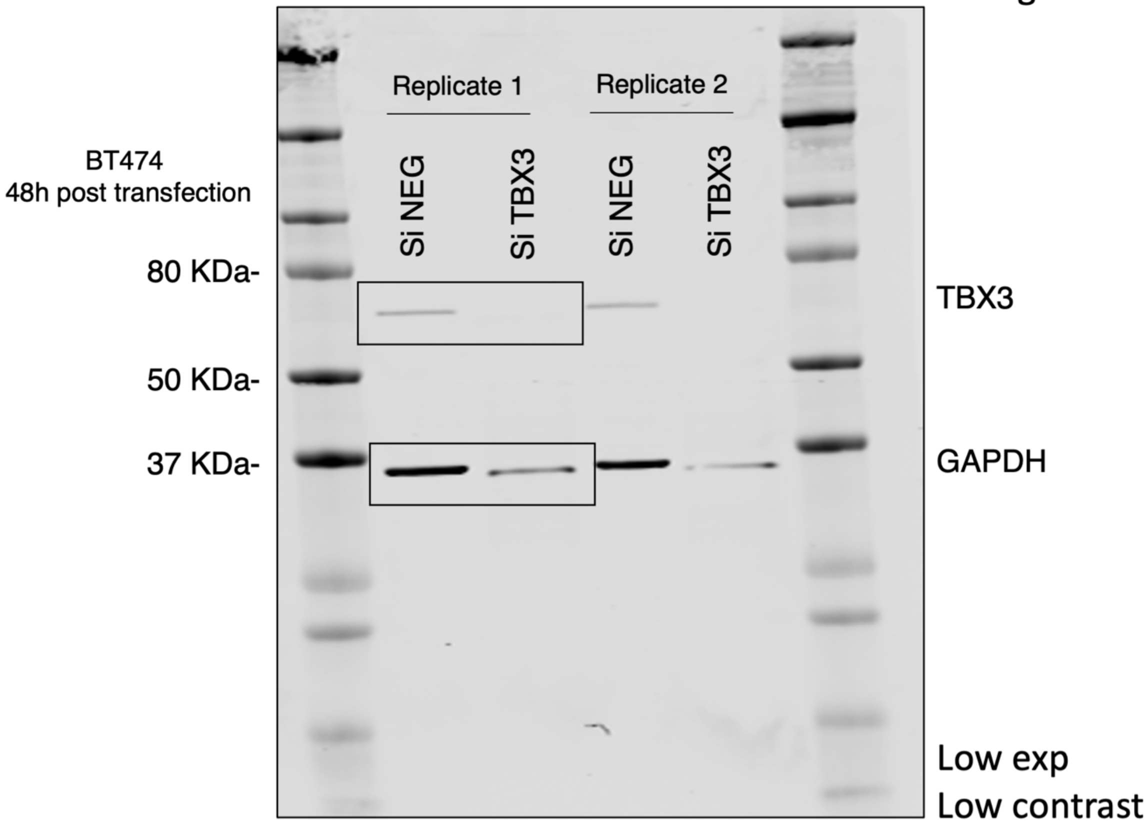
BT474
48h post transfection
Replicate 1
Replicate 2
Si NEG
Si TBX3
Si NEG
Si TBX3
80 KDa-
50 KDa-
37 KDa-
TBX3
GAPDH
Low exp
Low contrast

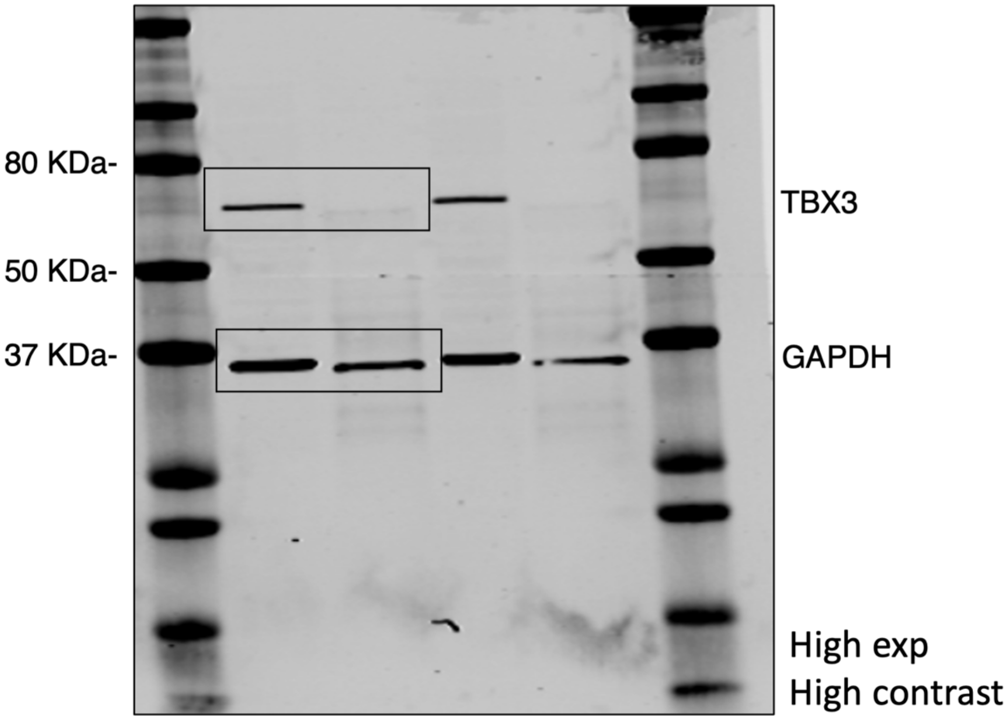
80 KDa-
50 KDa-
37 KDa-
TBX3
GAPDH
High exp
High contrast

Supplementary figure S1A (genomic DNA PCR)

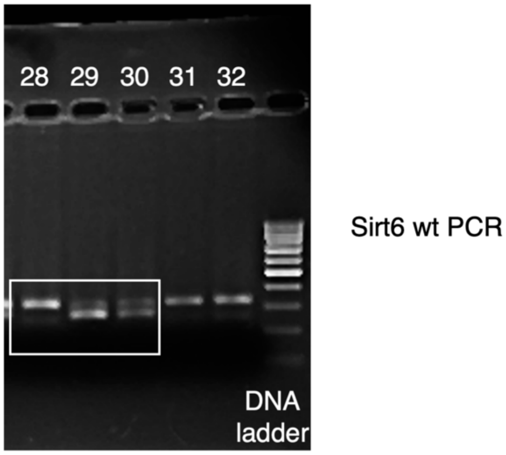

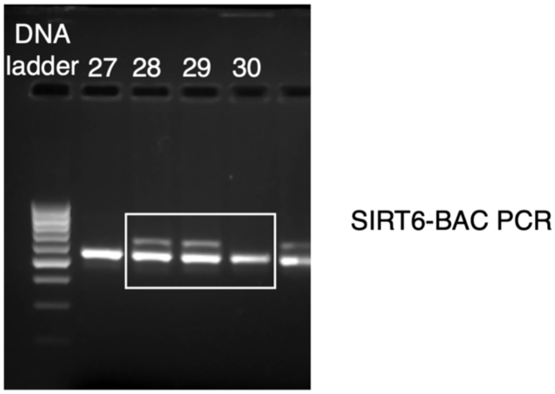

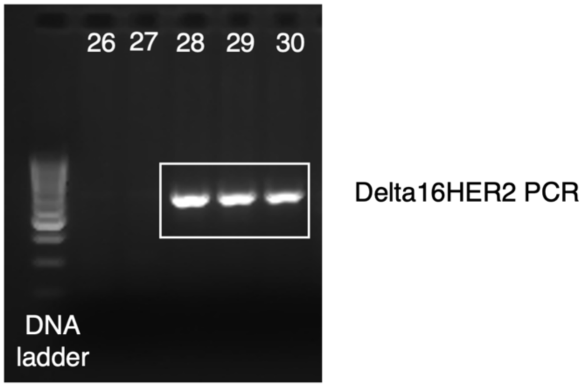

Supplementary Figure S4A

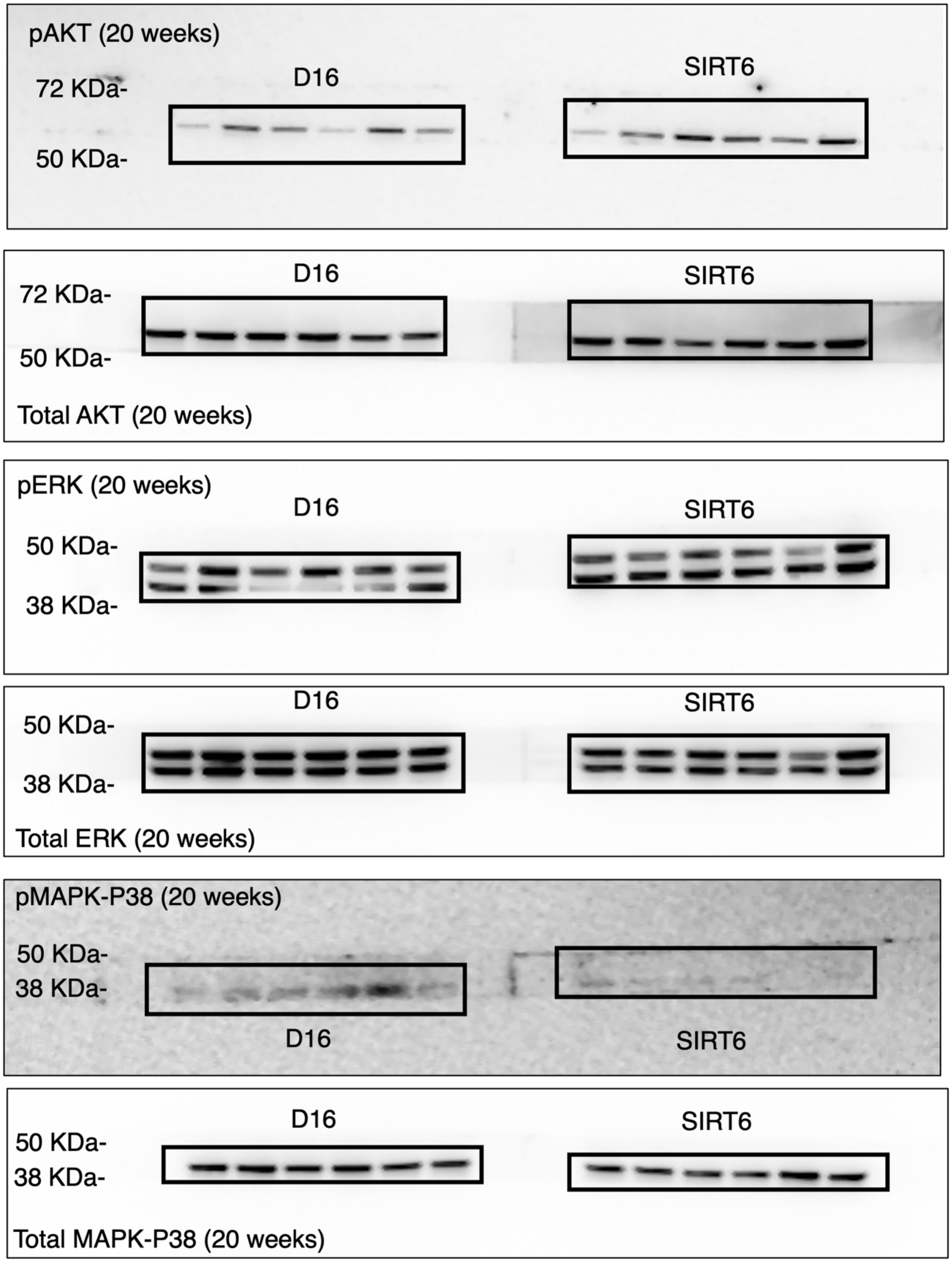

Supplementary Figure S4A
(short exposure, low contrast)

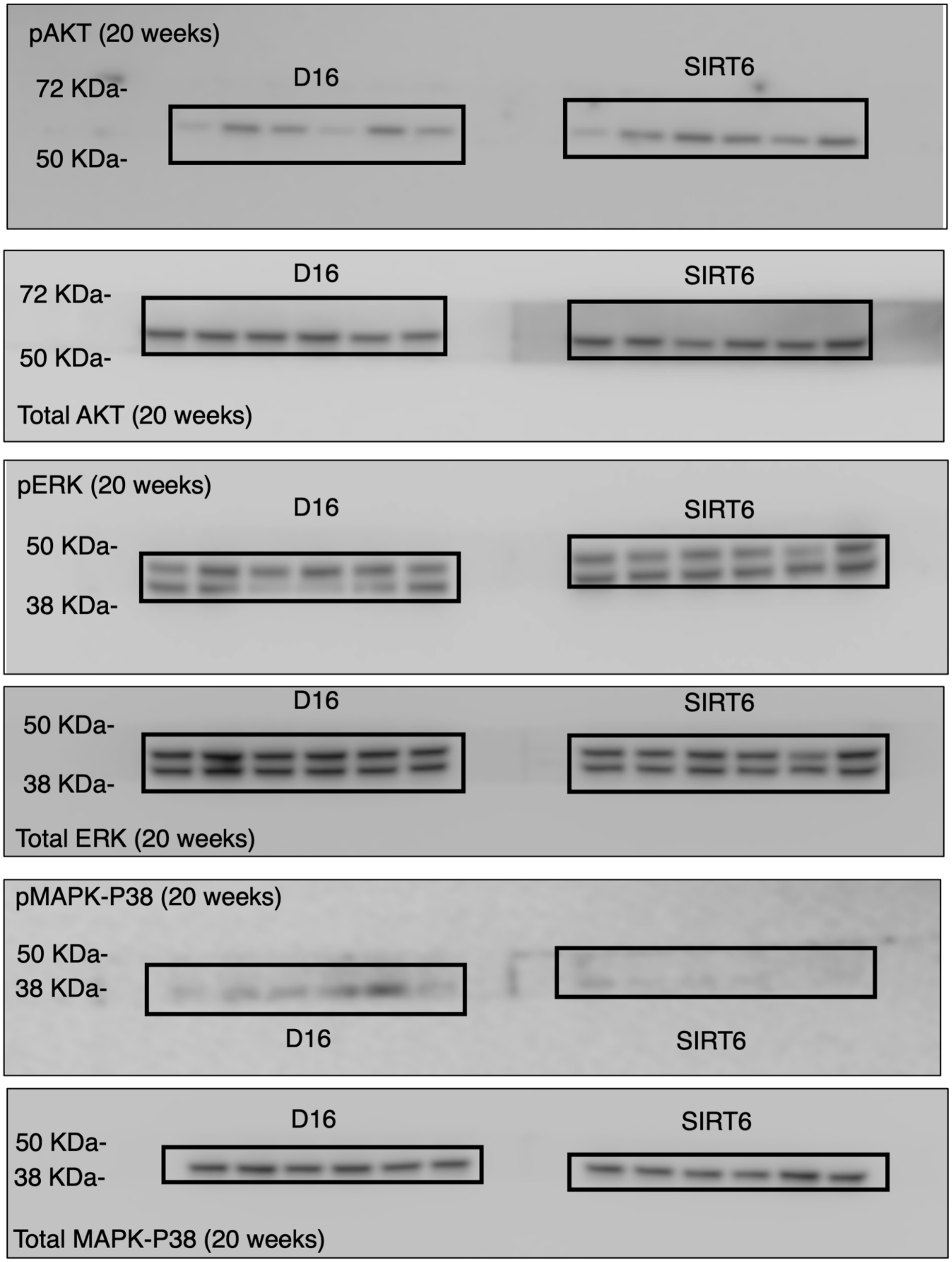

Additional Replicates for supplementary Figure S4A

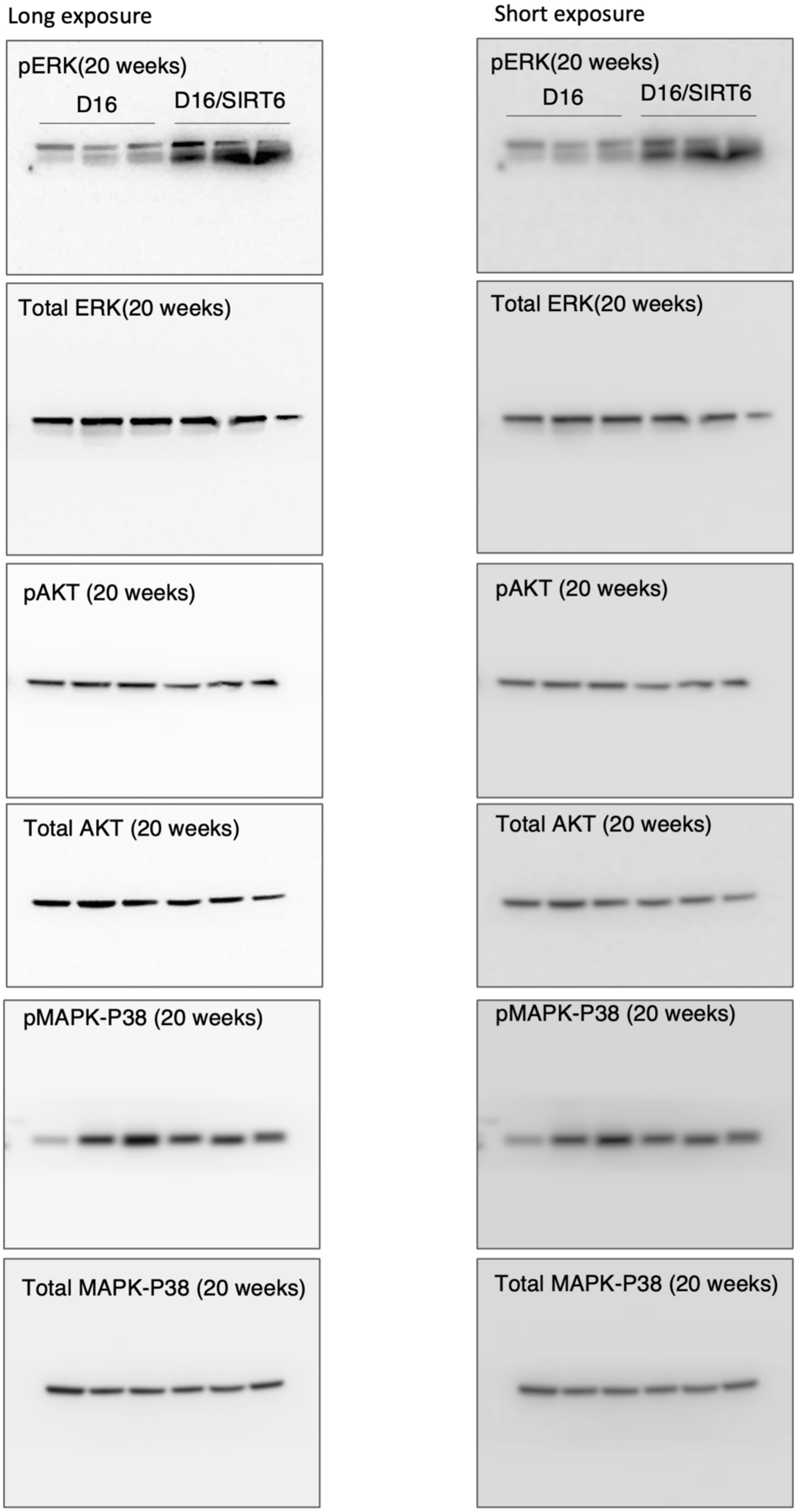

Supplementary Figure S4C

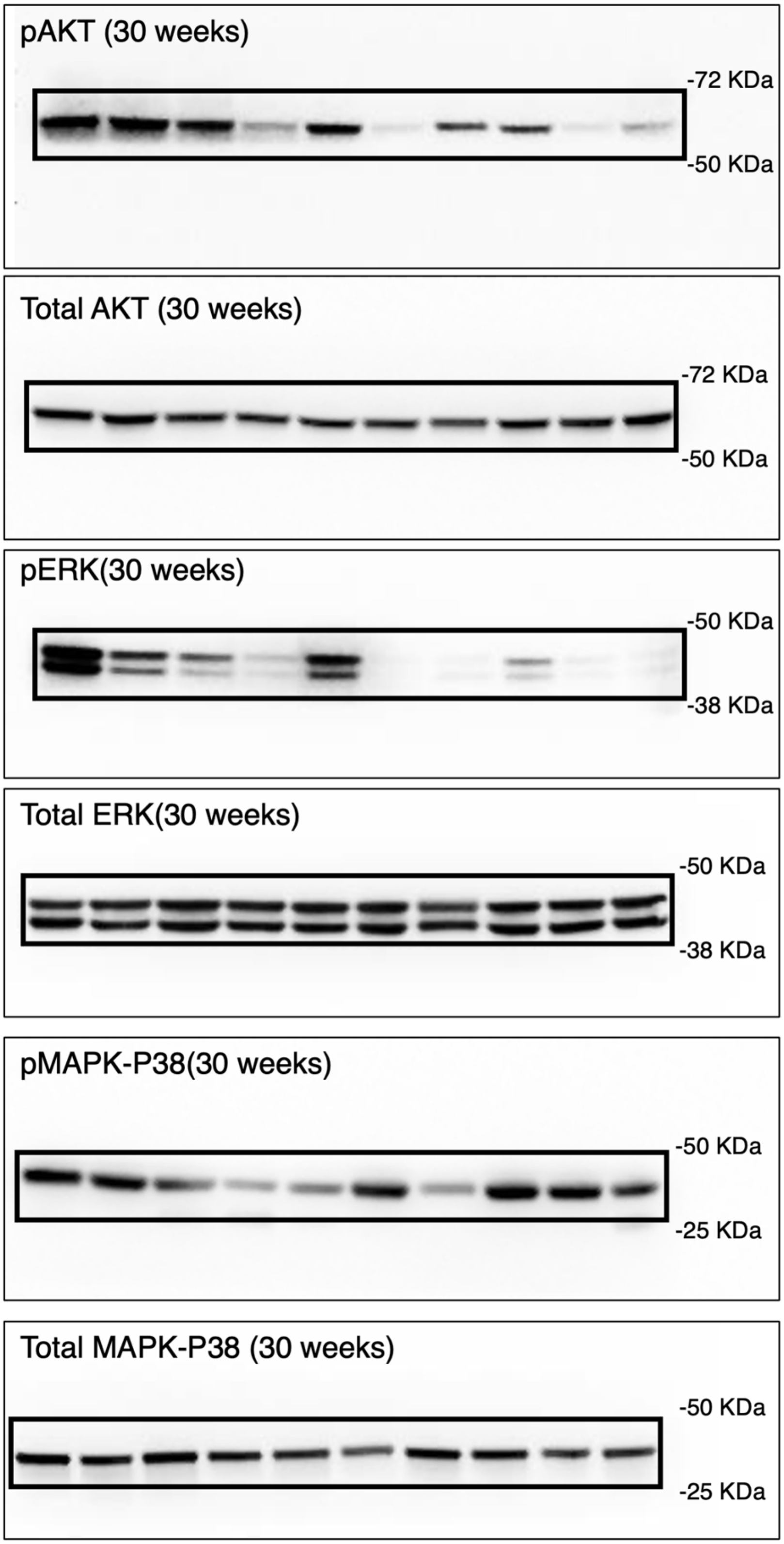

Supplementary Figure 4C
(short exposure, low contrast)

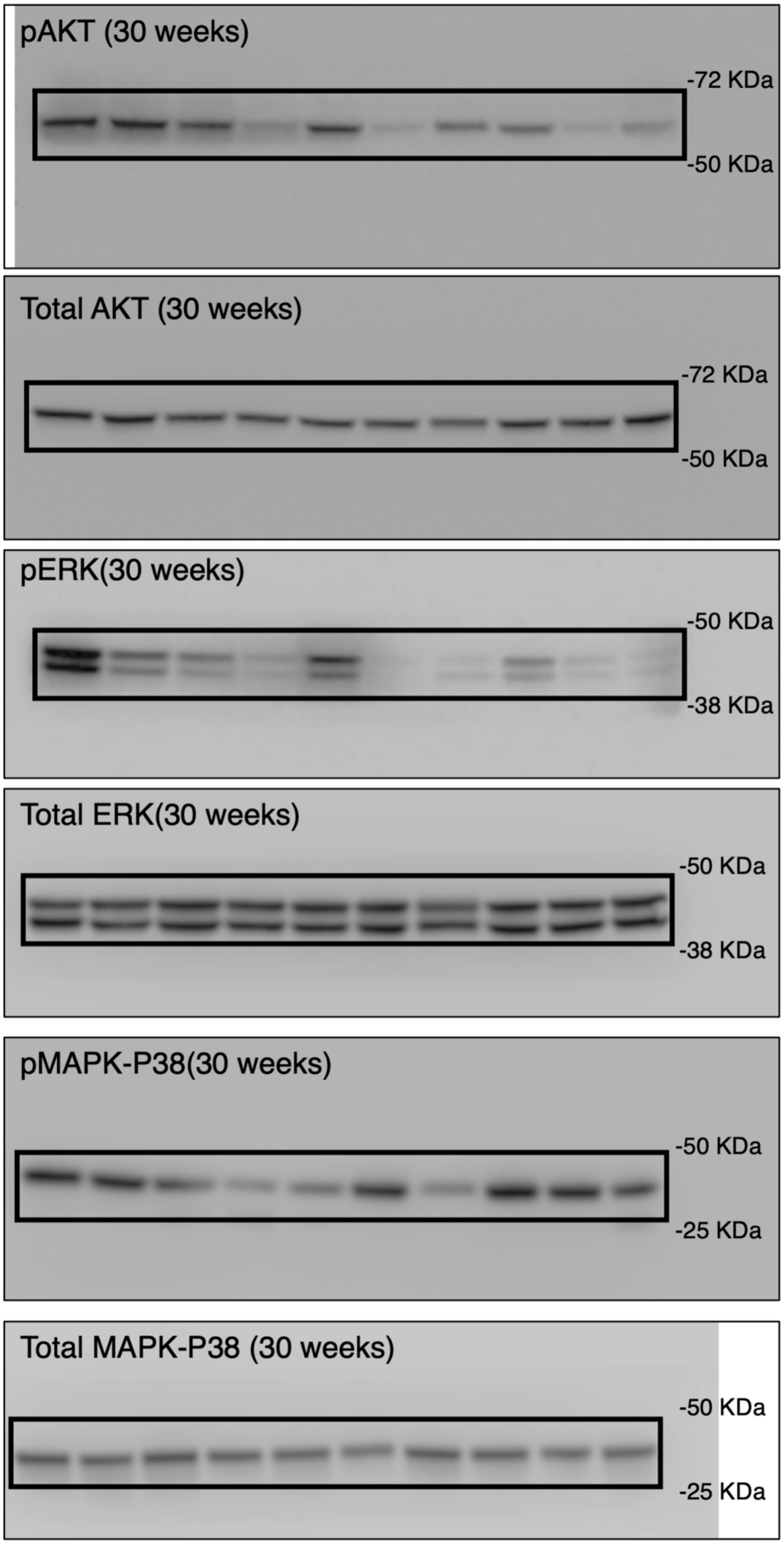

Additional Replicates for supplementary Figure S4C

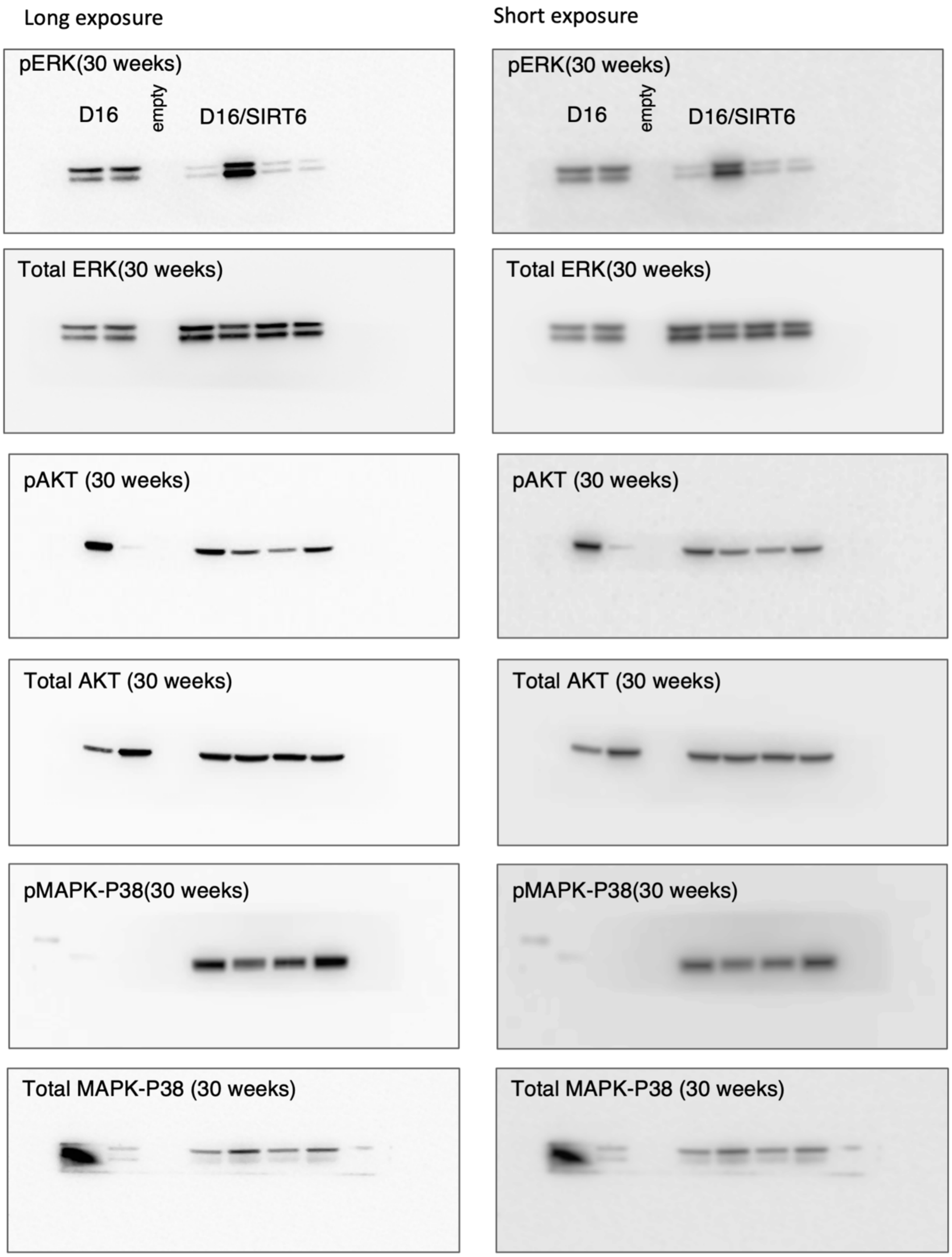

Supplementary FIGURE 5A

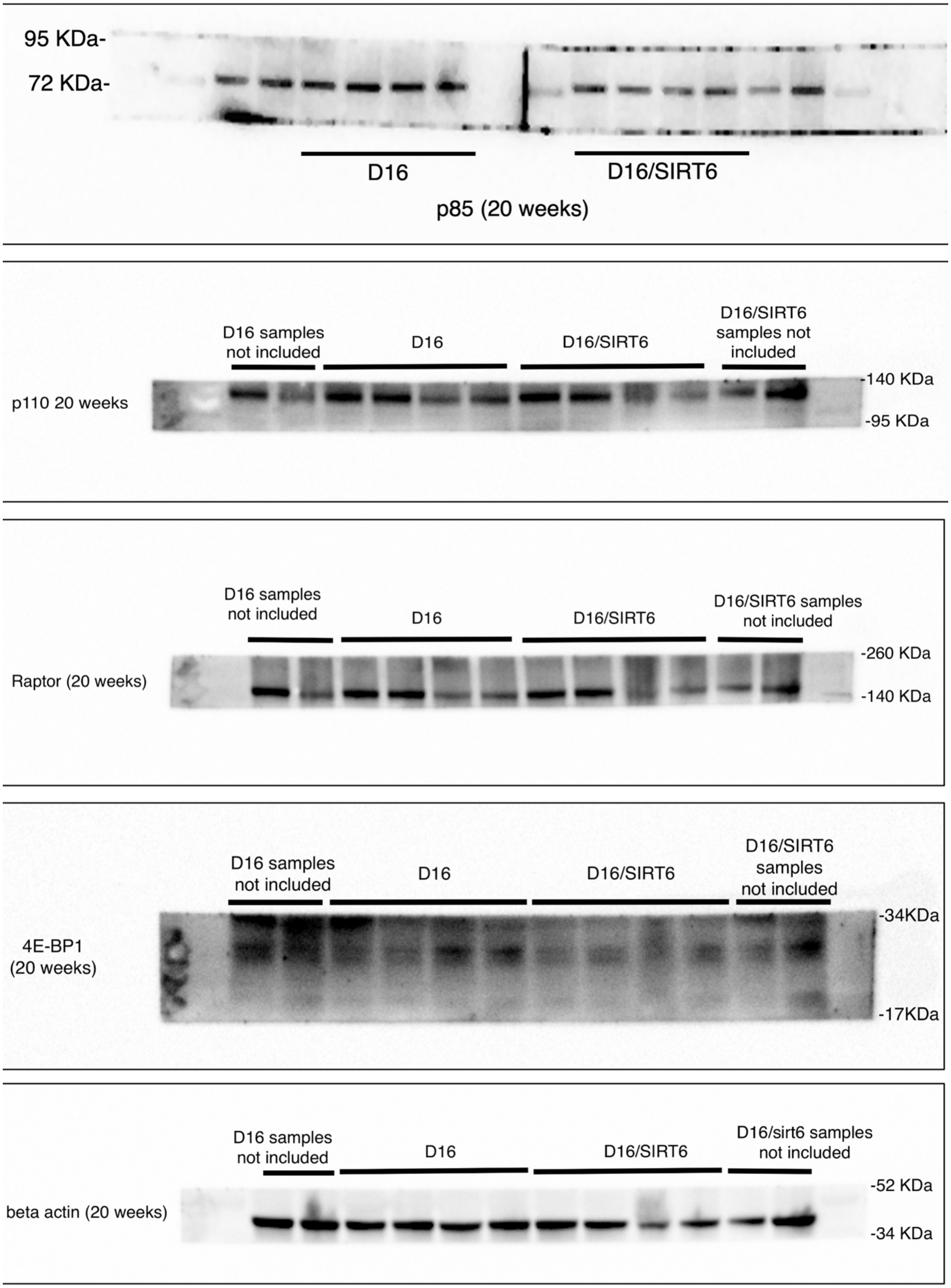

Supplementary FIGURE 5A
(short exposure, low contrast)

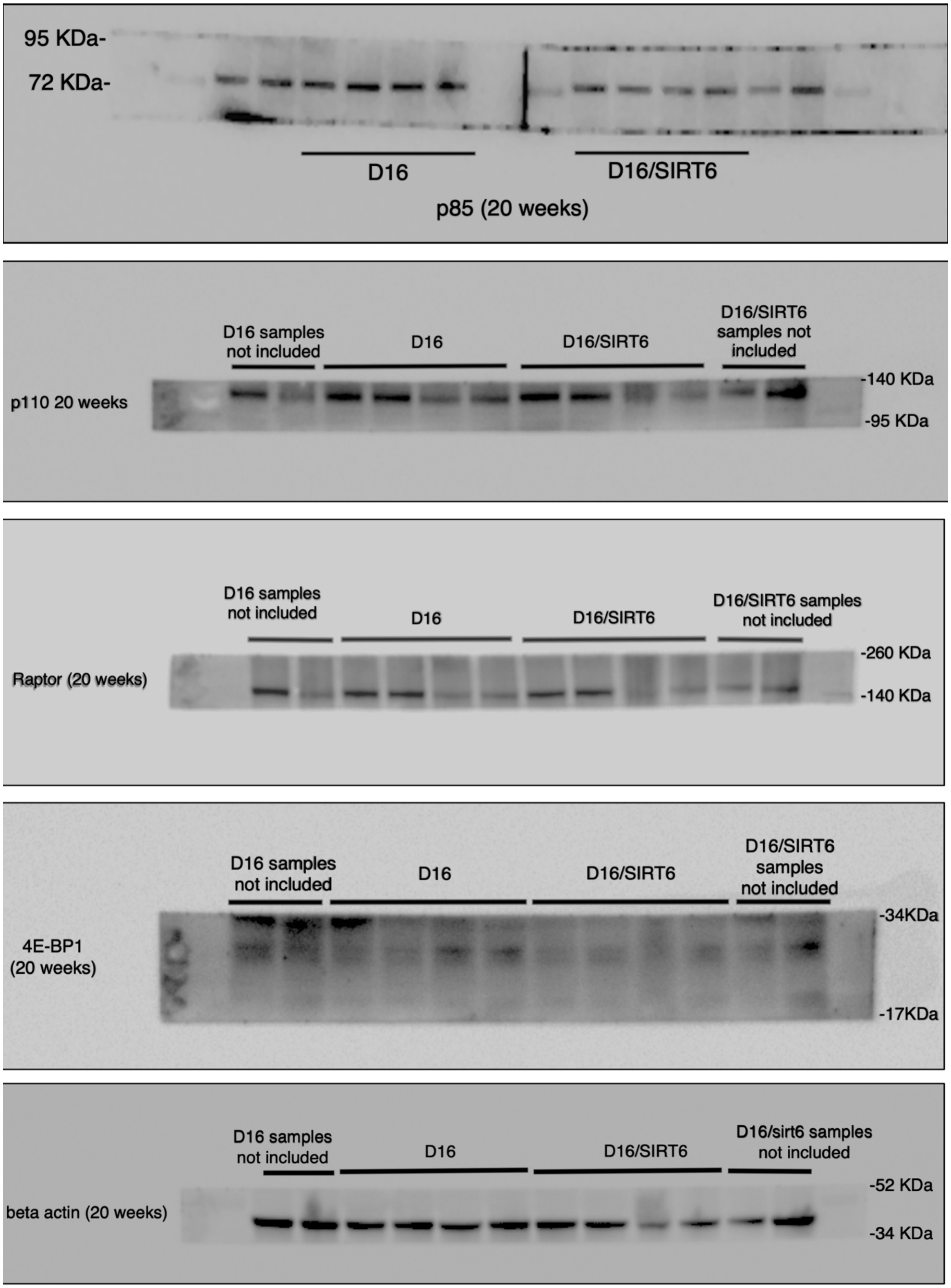

Additional Replicates for supplementary Figure S5A

Long exposure

Short exposure

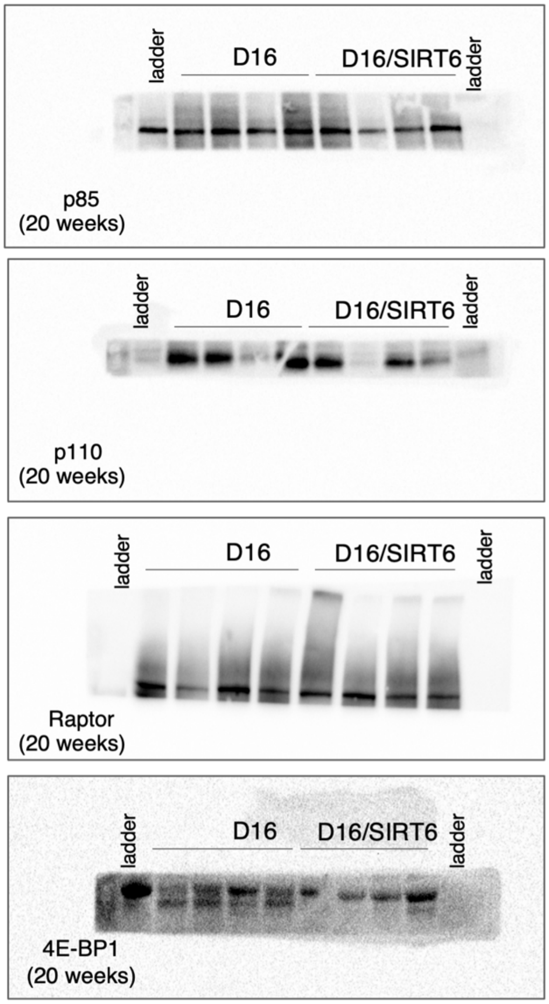

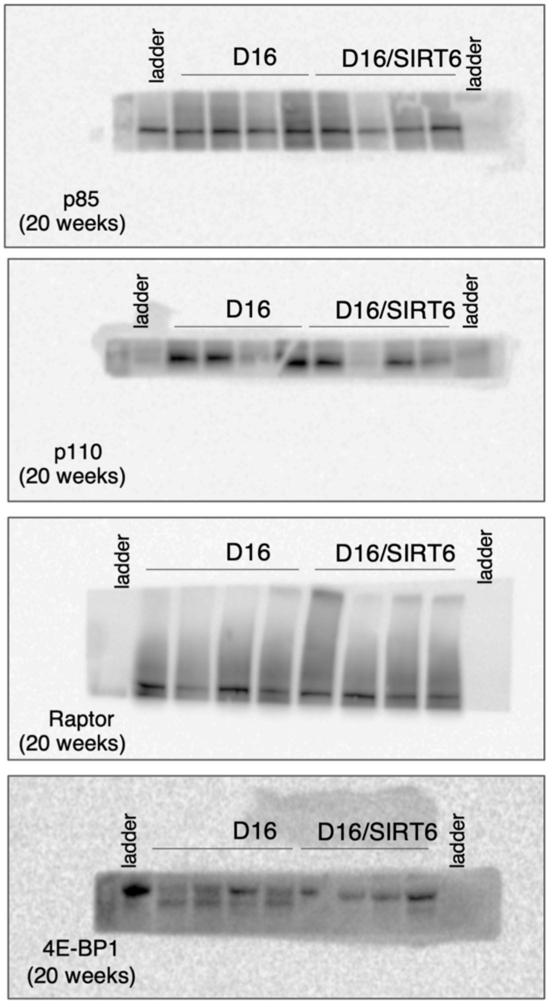

Supplementary Figure S5C

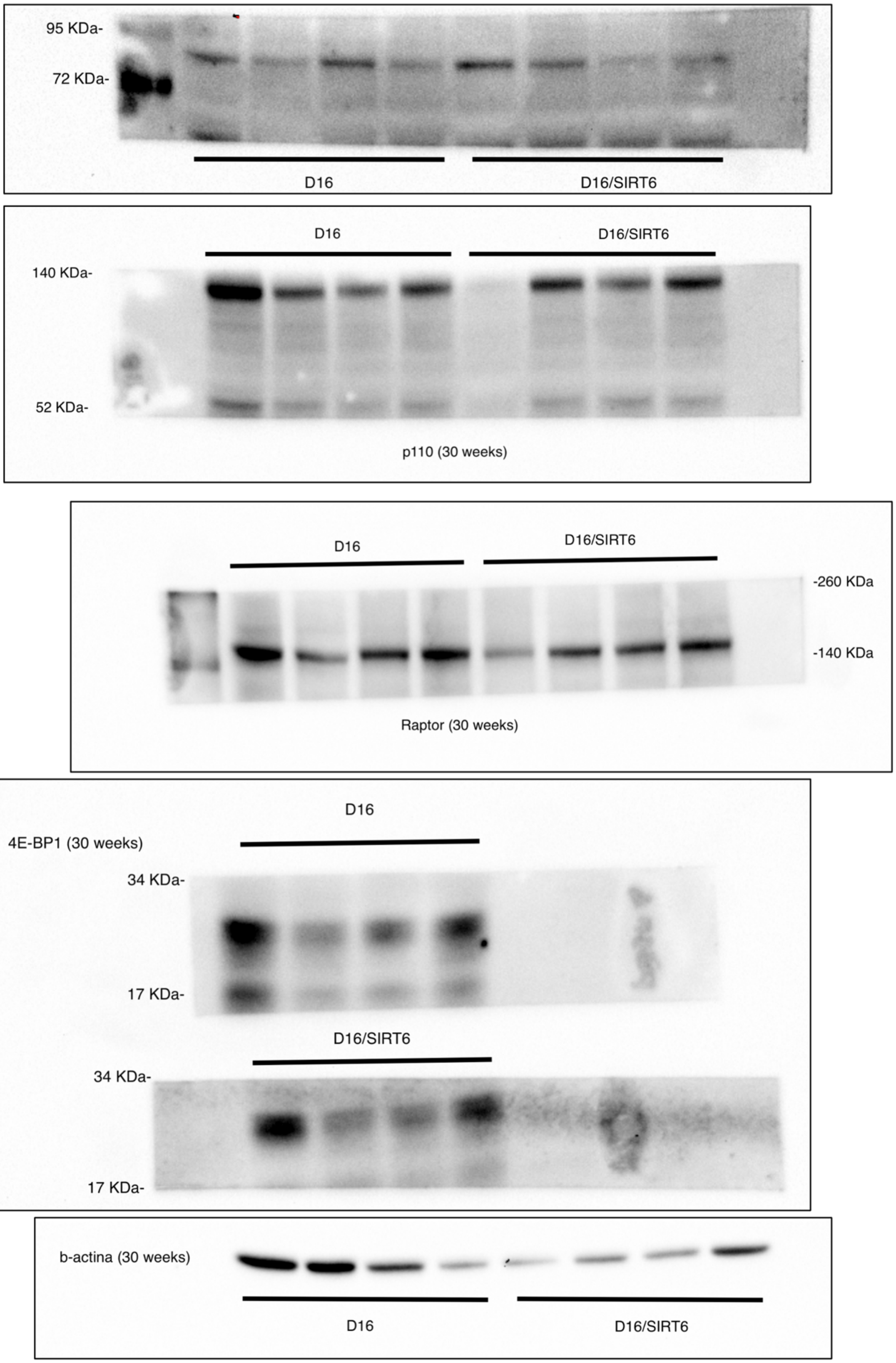

Supplementary Figure S5C
(short exposure/low contrast)

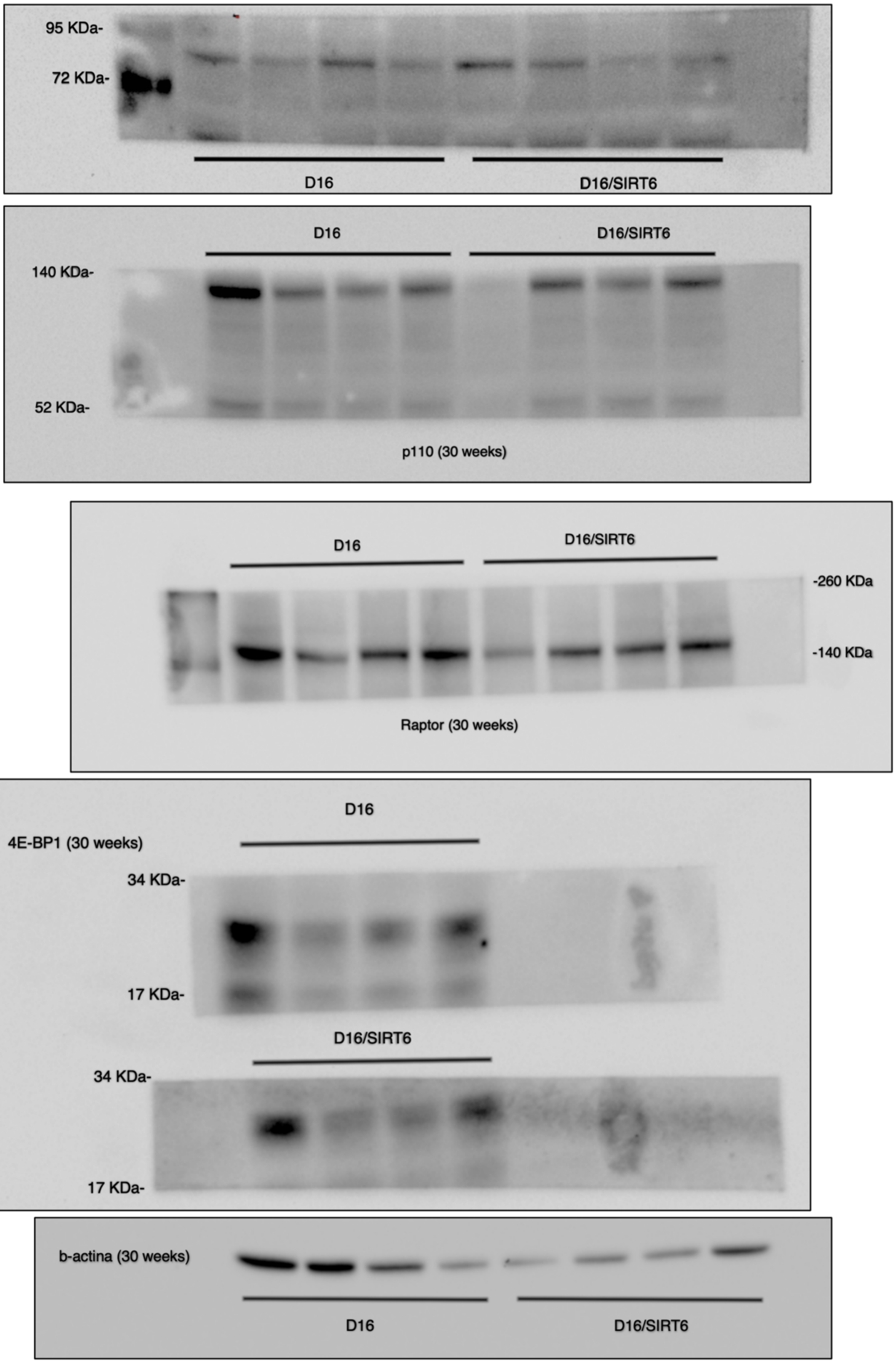

Additional Replicates for supplementary Figure S5C

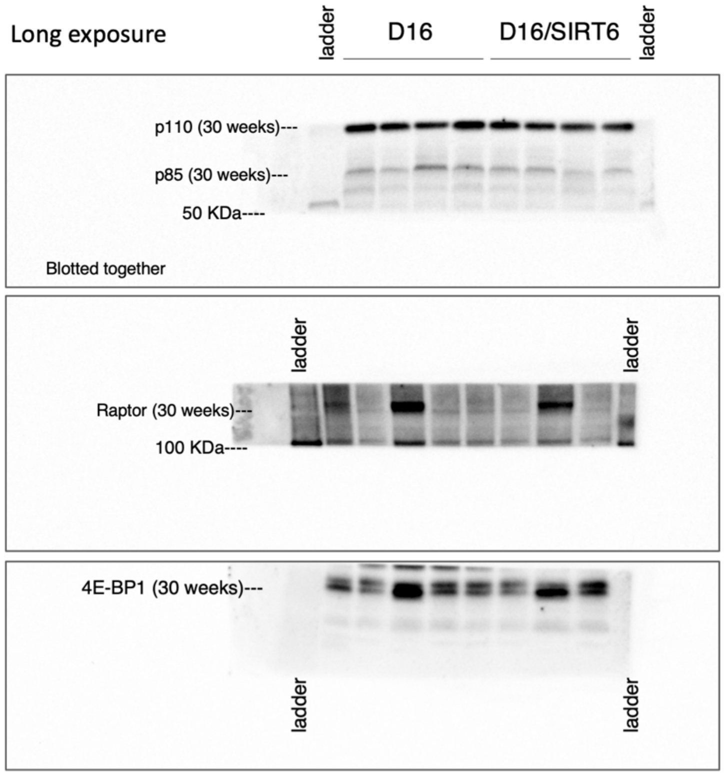

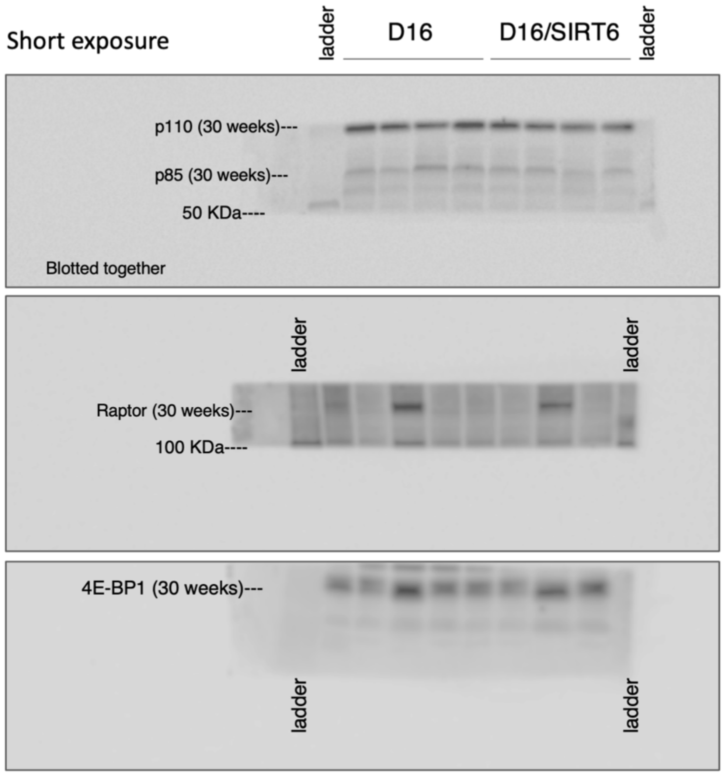

Supplementary Figure S6A

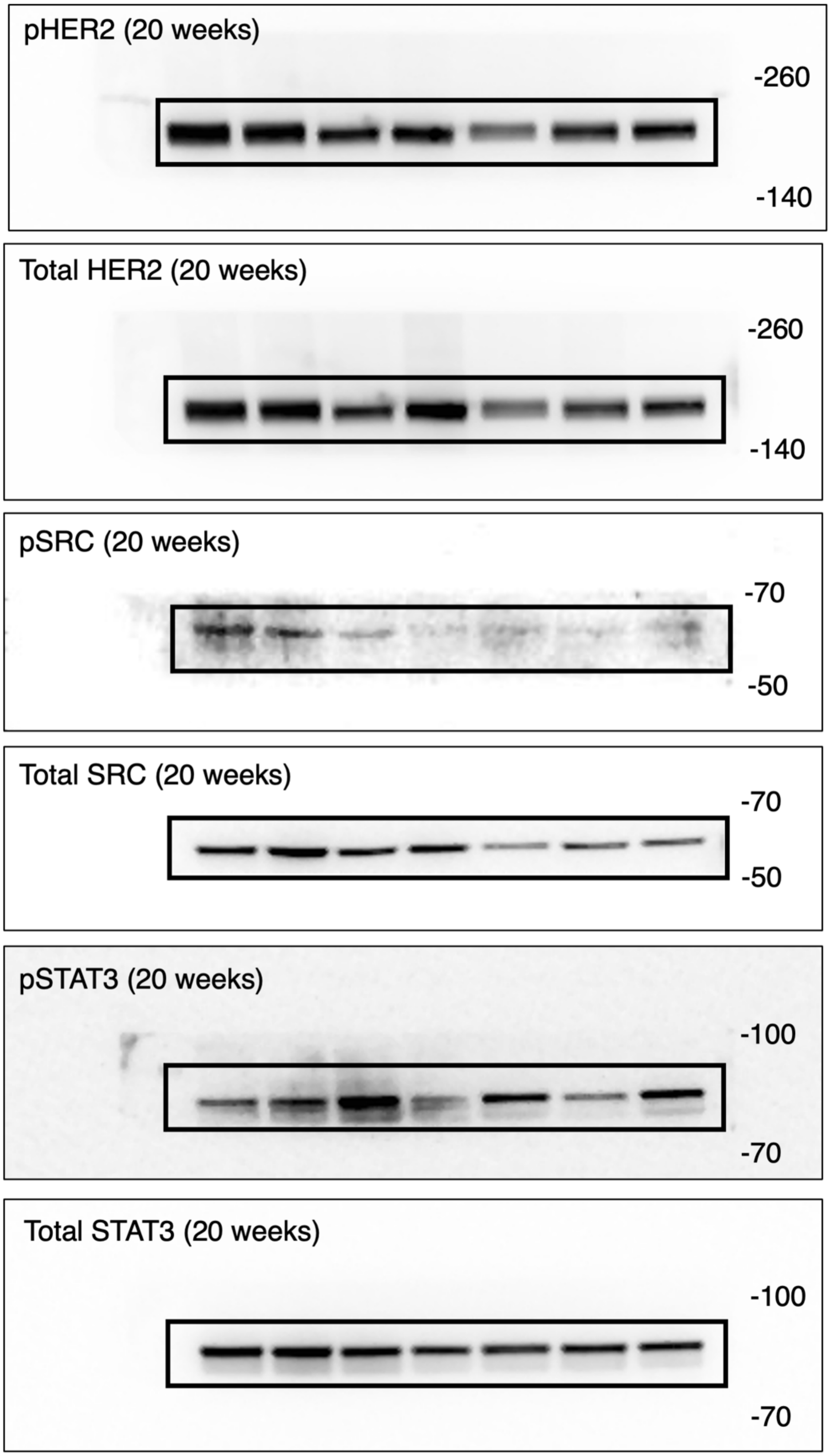

Supplementary Figure S6A
(short exposure/low contrast)

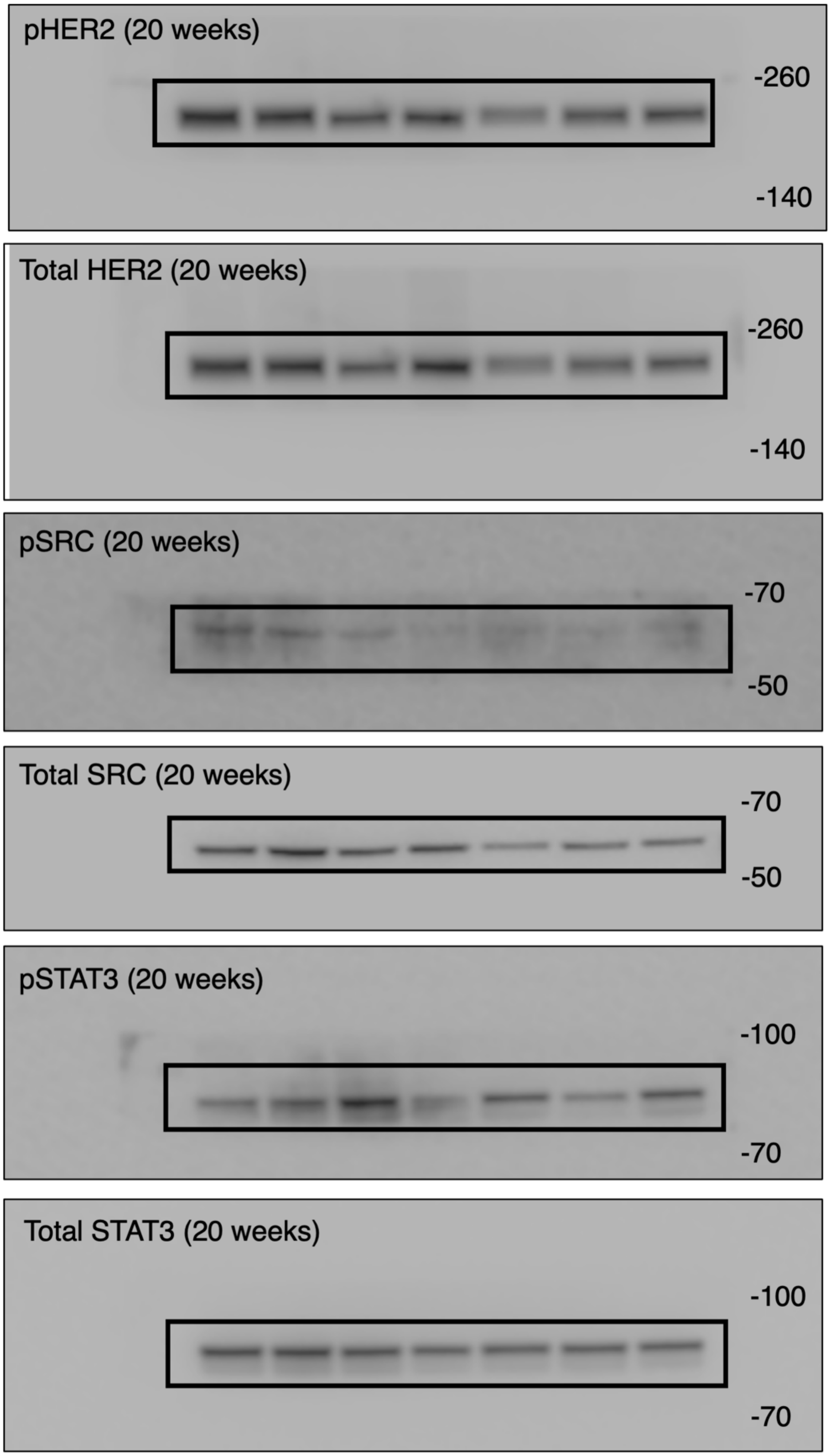

Additional Replicates for supplementary Figure S6A

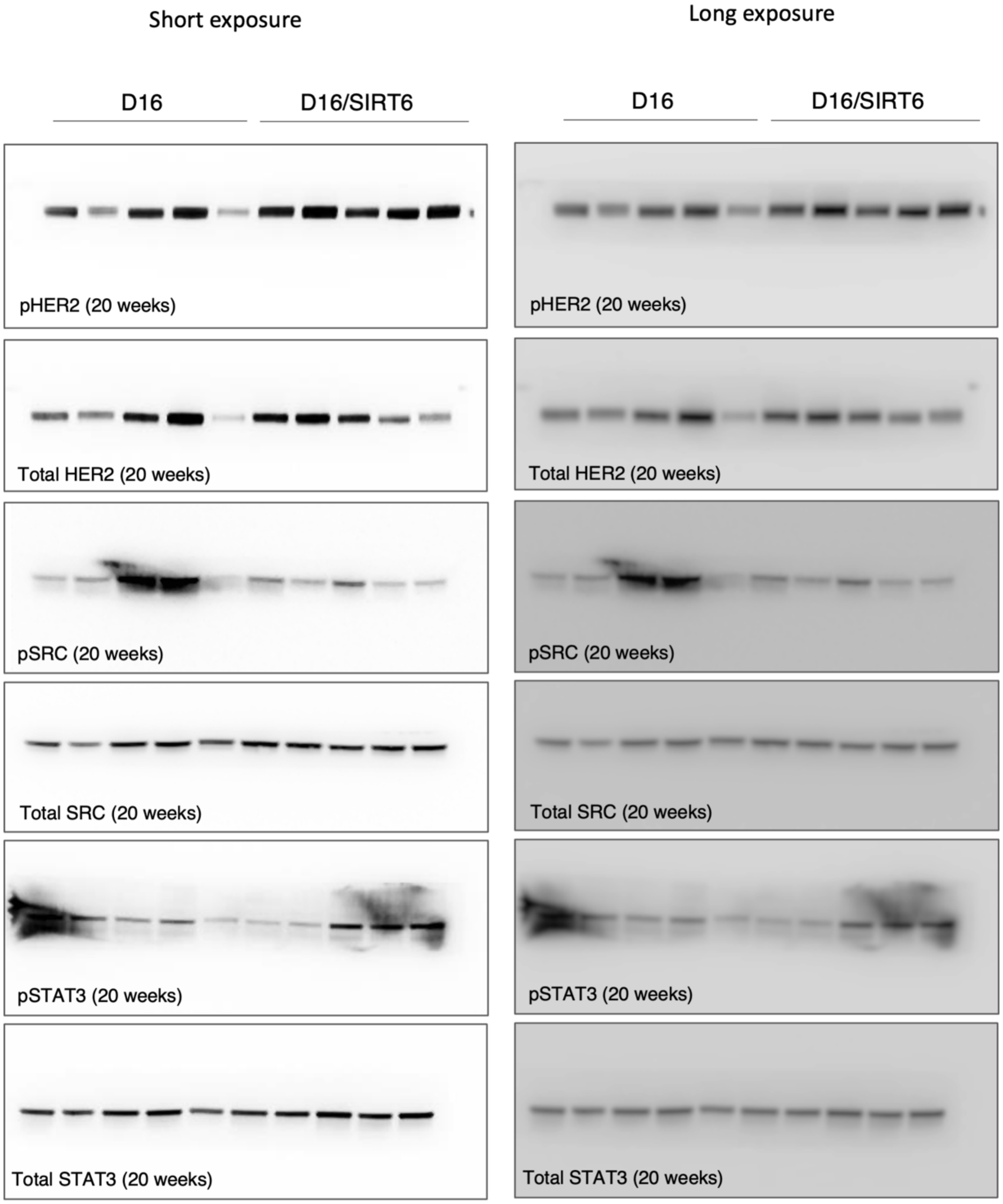

Supplementary Figure S6C

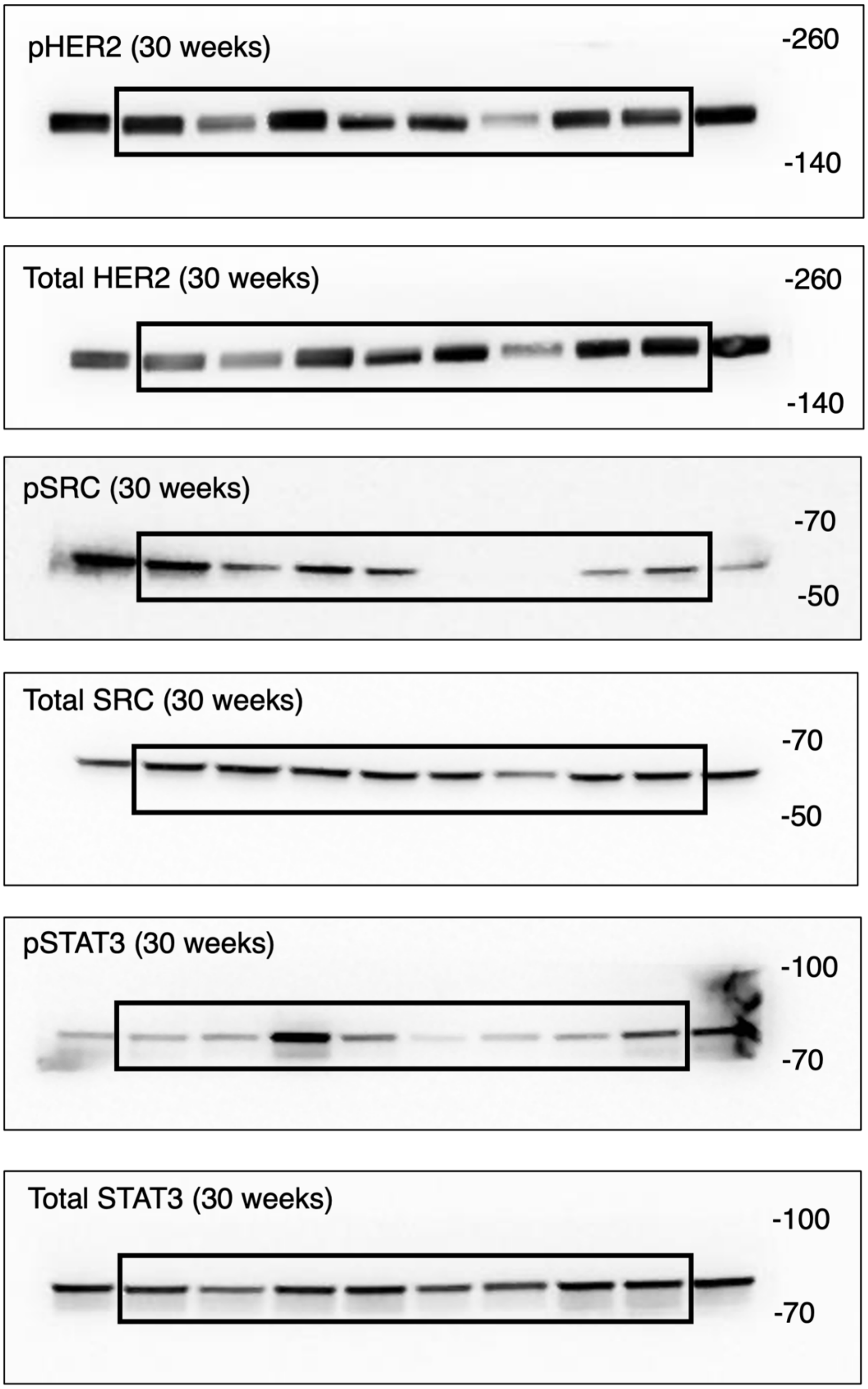

Supplementary Figure S6C
(short exposure/low contrast)

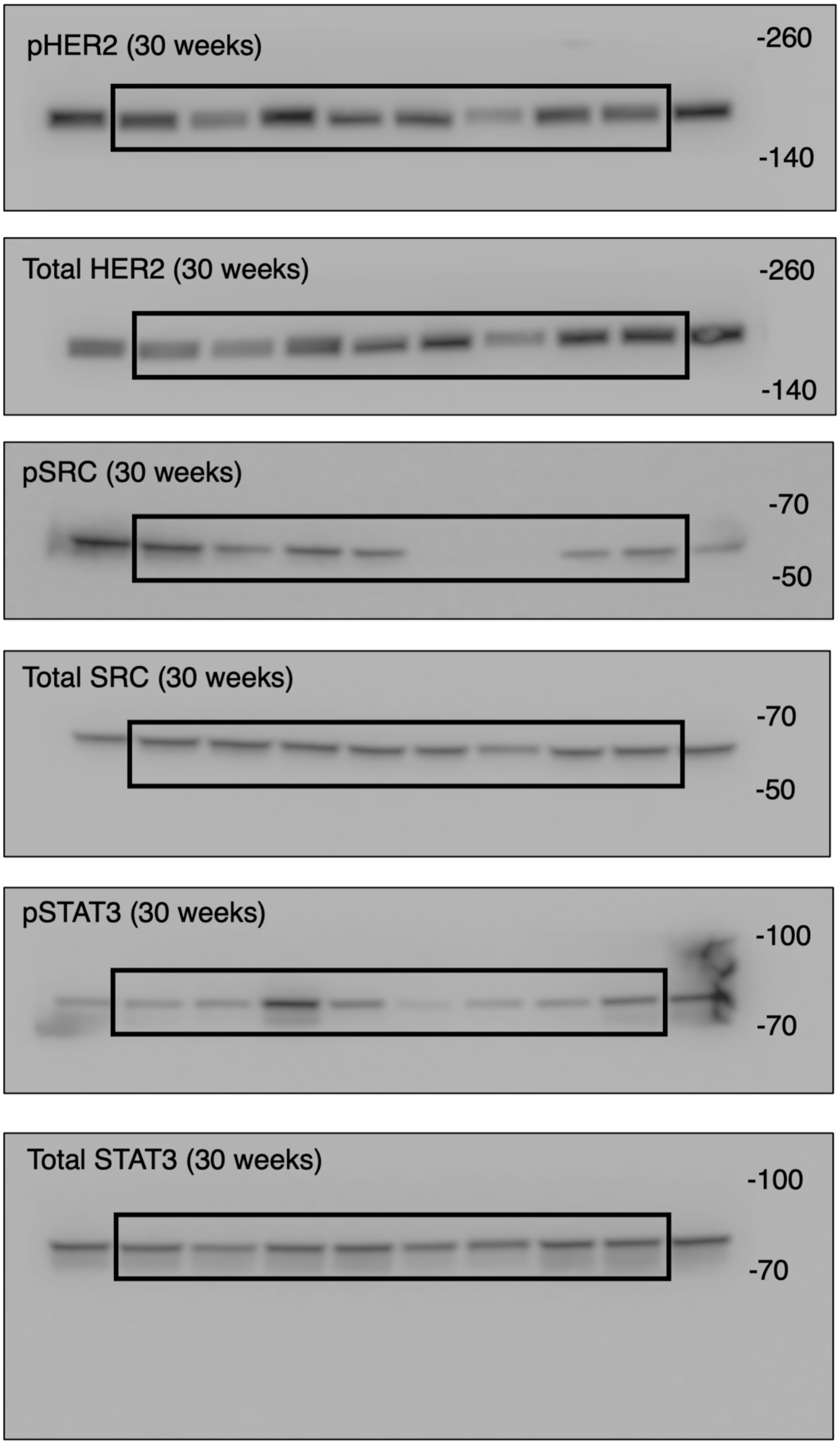

Additional Replicates for supplementary Figure S6C

Long exposure

Short exposure

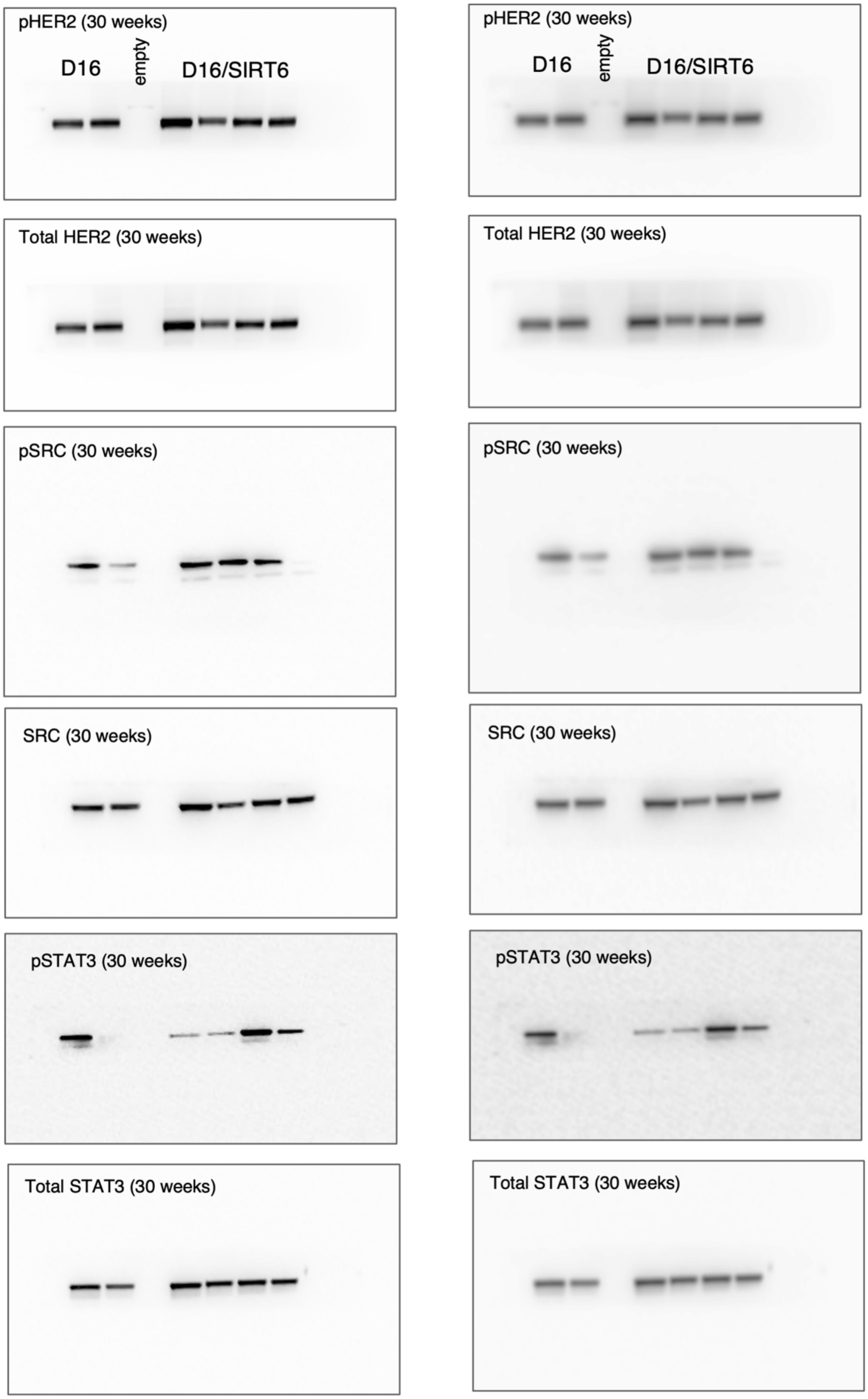

Supplementary Figure 9

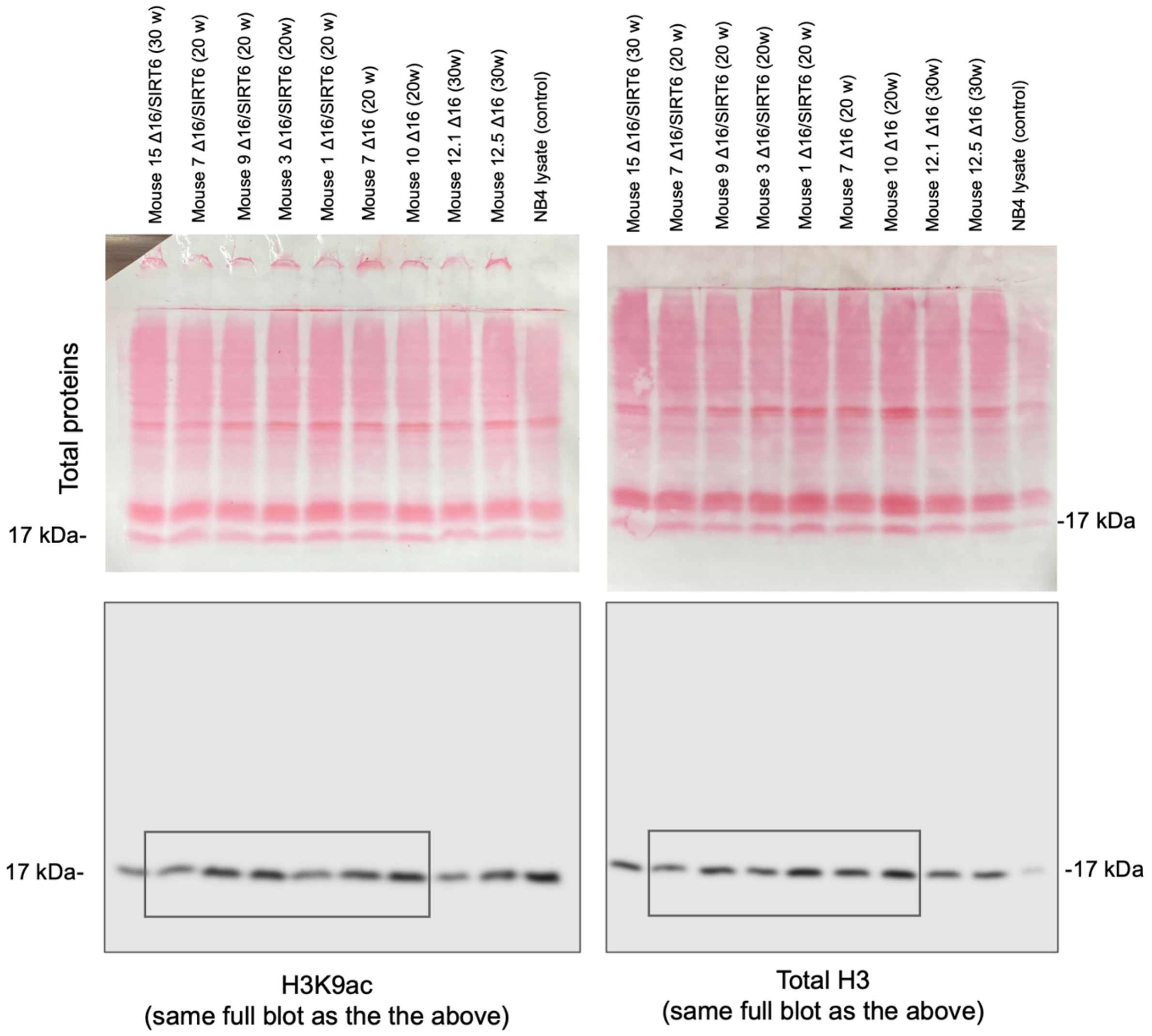

Supplementary Figure 13A

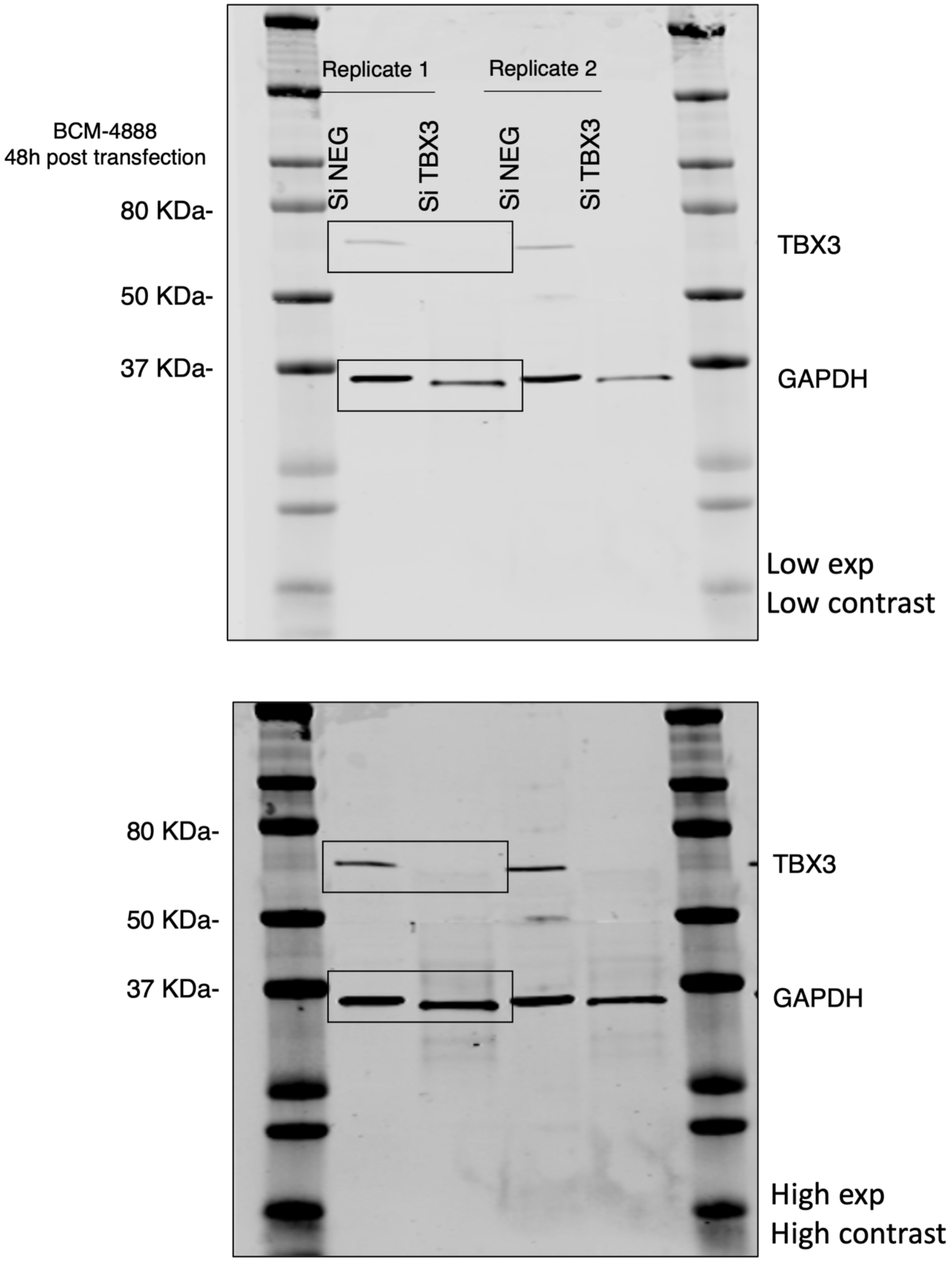

Supplement: Supplementary file 1 — Supplementary Figures. [file 41598_2023_49199_MOESM1_ESM.pdf]
